# Patient-Specific Network Connectivity Combined With a Next Generation Neural Mass Model to Test Clinical Hypothesis of Seizure Propagation

**DOI:** 10.3389/fnsys.2021.675272

**Published:** 2021-09-01

**Authors:** Moritz Gerster, Halgurd Taher, Antonín Škoch, Jaroslav Hlinka, Maxime Guye, Fabrice Bartolomei, Viktor Jirsa, Anna Zakharova, Simona Olmi

**Affiliations:** ^1^Institut für Theoretische Physik, Technische Universität Berlin, Berlin, Germany; ^2^Inria Sophia Antipolis Méditerranée Research Centre, MathNeuro Team, Valbonne, France; ^3^National Institute of Mental Health, Klecany, Czechia; ^4^MR Unit, Department of Diagnostic and Interventional Radiology, Institute for Clinical and Experimental Medicine, Prague, Czechia; ^5^Institute of Computer Science of the Czech Academy of Sciences, Prague, Czechia; ^6^Faculté de Médecine de la Timone, Centre de Résonance Magnétique et Biologique et Médicale (CRMBM, UMR CNRS-AMU 7339), Medical School of Marseille, Aix-Marseille Université, Marseille, France; ^7^Assistance Publique -Hôpitaux de Marseille, Hôpital de la Timone, Pôle d'Imagerie, Marseille, France; ^8^Assistance Publique - Hôpitaux de Marseille, Hôpital de la Timone, Service de Neurophysiologie Clinique, Marseille, France; ^9^Aix Marseille Université, Inserm, Institut de Neurosciences des Systèmes, UMRS 1106, Marseille, France; ^10^Consiglio Nazionale delle Ricerche, Istituto dei Sistemi Complessi, Sesto Fiorentino, Italy

**Keywords:** neural mass model, quadratic integrate-and-fire neuron, patient-specific brain network model, epileptic seizure-like event, topological network measure

## Abstract

Dynamics underlying epileptic seizures span multiple scales in space and time, therefore, understanding seizure mechanisms requires identifying the relations between seizure components within and across these scales, together with the analysis of their dynamical repertoire. In this view, mathematical models have been developed, ranging from single neuron to neural population. In this study, we consider a neural mass model able to exactly reproduce the dynamics of heterogeneous spiking neural networks. We combine mathematical modeling with structural information from non invasive brain imaging, thus building large-scale brain network models to explore emergent dynamics and test the clinical hypothesis. We provide a comprehensive study on the effect of external drives on neuronal networks exhibiting multistability, in order to investigate the role played by the neuroanatomical connectivity matrices in shaping the emergent dynamics. In particular, we systematically investigate the conditions under which the network displays a transition from a low activity regime to a high activity state, which we identify with a seizure-like event. This approach allows us to study the biophysical parameters and variables leading to multiple recruitment events at the network level. We further exploit topological network measures in order to explain the differences and the analogies among the subjects and their brain regions, in showing recruitment events at different parameter values. We demonstrate, along with the example of diffusion-weighted magnetic resonance imaging (dMRI) connectomes of 20 healthy subjects and 15 epileptic patients, that individual variations in structural connectivity, when linked with mathematical dynamic models, have the capacity to explain changes in spatiotemporal organization of brain dynamics, as observed in network-based brain disorders. In particular, for epileptic patients, by means of the integration of the clinical hypotheses on the epileptogenic zone (EZ), i.e., the local network where highly synchronous seizures originate, we have identified the sequence of recruitment events and discussed their links with the topological properties of the specific connectomes. The predictions made on the basis of the implemented set of exact mean-field equations turn out to be in line with the clinical pre-surgical evaluation on recruited secondary networks.

## 1. Introduction

Epilepsy is a chronic neurological disorder characterized by the occurrence and recurrence of seizures and represents the third most common neurological disorder affecting more than 50 million people worldwide (World Health Organization, 2005). Anti-epileptic drugs are the first line of treatment for epilepsy, and they provide sufficient seizure control in around two-thirds of cases (Kwan and Brodie, 2000). However, about 30–40% of epilepsy patients do not respond to drugs, a percentage that has remained relatively stable despite significant efforts to develop new anti-epileptic medication over the past decades. For drug-resistant patients, a possible treatment is the surgical resection of the brain tissue responsible for the generation of seizures.

As a standard procedure, epilepsy surgery is preceded by a qualitative assessment of different brain imaging modalities to identify the brain tissue responsible for seizure generation, i.e., the epileptogenic zone (EZ) (Rosenow and Lüders, 2001), which in general represents a localized region or a network where seizures arise, before recruiting secondary networks, called the propagation zone (PZ) (Talairach and Bancaud, 1966; Bartolomei et al., 2001; Spencer, 2002; Richardson, 2012). Outcomes are positive whenever the patient has become seizure-free after surgical operation.

Intracranial electroencephalography (iEEG) is commonly used during the presurgical assessment to find the seizure onset zone (Rosenow and Lüders, 2001; David et al., 2011; Duncan et al., 2016), the assumption being that the region where seizures emerge, is at least part of the brain tissue responsible for seizure generation. As a part of the standard presurgical evaluation with iEEG, stereotactic EEG (SEEG) is used to help correctly delineating the EZ (Bartolomei et al., 2002). SEEG employs penetrating depth electrodes that are implanted through small burr holes in the skull and are positioned using stereotactic guidance (Talairach and Bancaud, 1966), thus allowing for the measurement of neural activity in deeper structures of the brain. Alternative imaging techniques such as structural Magnetic Resonance Imaging (MRI), magneto- or electro-encephalography (M/EEG), and positron emission tomography (PET) help the clinician estimate the position of the EZ. Recently, diffusion MRI (dMRI) started being evaluated as well, thus giving the possibility to infer the connectivity between different brain regions by computing *in-vivo* fiber tract trajectories in-coherently organized brain white matter pathways (Basser et al., 2000). dMRI has revealed a quantitative decrease of regional connectivity around the EZ that is associated with a network reorganization and cognitive impairment (Leyden et al., 2015). In particular, it has revealed reduced fractional anisotropy (Ahmadi et al., 2009; Bernhardt et al., 2013) and structural alterations in the connectome of epileptic patients (Bonilha et al., 2012; Besson et al., 2014; DeSalvo et al., 2014). However, epilepsy surgery is often unsuccessful and the long-term positive outcome may be lower than 25% in extra-temporal cases (De Tisi et al., 2011; Najm et al., 2013), thus meaning that the EZ has not been correctly identified or that the EZ and the seizure onset zone may not coincide (Lopes et al., 2019).

To quantitatively examine clinical data and to determine targets for surgery, many computational models have been recently proposed (Freestone et al., 2013; Hutchings et al., 2015; Goodfellow et al., 2016; Khambhati et al., 2016; Lopes et al., 2017; Sinha et al., 2017; Karoly et al., 2018), that use MRI or iEEG data acquired during presurgical workup to infer structural or functional brain networks. Taking advantages of recent advances in the understanding of epilepsy, that indicate that seizures may arise from distributed ictogenic networks (Richardson, 2012; Bartolomei et al., 2017; Besson et al., 2017), phenomenological models of seizure transitions are used to compute the escape time, i.e., the time that each network node takes to transit from a normal state to a seizure-like state. Nodes with the lowest escape time are then considered as representative of the seizure onset zone and, therefore, candidates for surgical resection, by assuming seizure onset zone as a proxy for the EZ (Hutchings et al., 2015; Sinha et al., 2017). Alternatively, different possible surgeries are simulated *in silico* to predict surgical outcomes (Goodfellow et al., 2017; Lopes et al., 2017, 2019) by making use of synthetic networks and phenomenological network models of seizure generation. Further attention has been paid to studying how network structure and tissue heterogeneities underpin the emergence of focal and widespread seizure dynamics in synthetic networks of phase oscillators (Lopes et al., 2019, 2020).

More in general there is a vast and valuable literature on computational modeling in epilepsy, where two classes of models are used: (1) mean-field (macroscopic) models and (2) detailed (microscopic) network models. Mean-field models are often preferred over the more detailed models since they have fewer parameters and, thus, simplify the study of transitions from interictal to ictal states and the subsequent EEG analysis of data from patients with epilepsy. This is justified as the macroelectrodes used for EEG recordings represent the average local field potential arising from neuronal populations. Indeed, much effort has been made so far to explain the biophysical and dynamical nature of seizure onsets and offsets by employing neural mass models (Da Silva et al., 1974; Wendling et al., 2002; Kalitzin et al., 2010; Touboul et al., 2011; Baier et al., 2012; Goodfellow et al., 2012; Kramer et al., 2012; Jirsa et al., 2014; Karoly et al., 2018). Mechanistic interpretability of the mean-field parameters is lost, as many physiological details are absorbed in few degrees of freedom. Since the mean-field models remain relatively simple, they can also be employed to describe epileptic processes occurring in “large-scale” systems, e.g., the precise identification of brain structures that belong to the seizure-triggering zone (epileptic activity often spreads over quite extended regions and involves several cortical and sub-cortical structures). However, only recently, the propagation of epileptic seizures was started to be studied using brain network models and was limited to a small number of populations (Terry et al., 2012) or absence seizures (Taylor et al., 2013), while partial seizures have been reported to propagate at the mesoscopic scale through cortical columns (Kramer et al., 2007; Goodfellow et al., 2011) and at the macroscopic scale through large-scale networks in humans (Bartolomei et al., 2013) and animal models (Toyoda et al., 2013). All in all, even though neural mass models are in general easier to analyze numerically because relatively few variables and parameters are involved, they drastically fail to suggest molecular and cellular mechanisms of epileptogenesis.

On the other hand, detailed network models are best suited for understanding the molecular and cellular bases of epilepsy and, thus, they may be used to suggest therapeutics targeting molecular pathways (Destexhe and Sejnowski, 1995; Van Drongelen et al., 2005; Turrigiano, 2008; Cressman et al., 2009; Ullah et al., 2009). Due to the substantial complexity of neuronal structures, relatively few variables and parameters can be accessed at any time experimentally. Although biophysically explicit modeling is the primary technique to look into the role played by experimentally inaccessible variables in epilepsy, the usefulness of detailed biophysical models is limited by constraints in computational power, uncertainties in detailed knowledge of neuronal systems, and the required simplification for the numerical analysis. Therefore, an intermediate “across-scale” approach, establishing relationships between sub-cellular/cellular variables of detailed models and mean-field parameters governing macroscopic models, might be a promising strategy to cover the gaps between these two modeling approaches (Brocke et al., 2016; Lindroos et al., 2018; Schirner et al., 2018).

In view of developing a cross-scale approach, it is important to point out that a large-scale brain network models emphasize the network character of the brain and merge structural information of individual brains with mathematical modeling, thus constituting *in-silico* approaches for the exploration of causal mechanisms of brain function and clinical hypothesis testing (Proix et al., 2017, 2018; Olmi et al., 2019). In particular, in brain network models, a network region is a neural mass model of neural activity, connected to other regions *via* a connectivity matrix representing fiber tracts of the human brain. This form of virtual brain modeling (Fuchs et al., 2000; Jirsa et al., 2002, 2010) exploits the explanatory power of network connectivity imposed as a constraint upon network dynamics and has provided important insights into the mechanisms underlying the emergence of asynchronous and synchronized dynamics of wakefulness and slow-wave sleep (Goldman et al., 2020) while revealing the whole-brain mutual coupling between the neuronal and the neurotransmission systems to understand the flexibility of human brain function despite having to rely on fixed anatomical connectivity (Kringelbach et al., 2020). Recent studies have pointed out the influence of individual structural variations of the connectome upon the large-scale brain network dynamics of the models, by systematically testing the virtual brain approach along with the example of epilepsy (Proix et al., 2017, 2018; Olmi et al., 2019). The employment of patient-specific virtual brain models derived from dMRI may have a substantial impact on personalized medicine, allowing for an increase in predictivity concerning the pathophysiology of brain disorders, and their associated abnormal brain imaging patterns. More specifically a personalized brain network model derived from non-invasive structural imaging data would allow for testing of clinical hypotheses and exploration of novel therapeutic approaches.

To exploit the predictive power of personalized brain network models, we have implemented, on subject-specific connectomes, a next-generation neural mass model that, differently from the previous applied heuristic mean-field models (Proix et al., 2017, 2018; Olmi et al., 2019), is exactly derived from an infinite size network of quadratic integrate-and-fire neurons (Montbrió et al., 2015), that represent the normal form of Hodgkin's class I excitable membranes (Ermentrout and Kopell, 1986). This next generation neural mass model can describe the variation of synchrony within a neuronal population, which is believed to underlie the decrease or increase of power seen in given EEG frequency bands while allowing for a more direct comparison with the results of electrophysiological experiments like local field potential, EEG, and event-related potentials (ERPs), thanks to its ability to capture the macroscopic evolution of the mean membrane potential. Most importantly, the exact reduction dimension techniques at the basis of the next-generation neural mass model have been developed for coupled phase oscillators (Ott and Antonsen, 2008) and allow for an exact (analytical) moving upward through the scales: While keeping the influence of smaller scales on larger ones, they level out their inherent complexity. In this way it is, therefore, possible to develop an intermediate “across-scale” approach exploiting the 1:1 correspondence between the microscopic and mesoscopic level that allows for more detailed modeling parameters and for mapping the microscopic results to the relative ones in the regional mean-field parameters.

The next-generation neural mass model developed by Montbrió et al. (2015), has been recently extended to take into account time-delayed synaptic coupling (Pazó and Montbrió, 2016; Devalle et al., 2018), and when integrated with a large-scale brain network, time delays in the interaction between the different brain areas, due to the finite conduction speed along fiber tracts of different lengths (Rabuffo et al., 2020). The time delay, together with the effective stochasticity of the investigated dynamics give rise, both on structural connectivity matrices of mice and healthy subjects, to preferred spatiotemporal pattern formation (Jirsa, 2008; Petkoski and Jirsa, 2020) and short-lived neuronal cascades that form spontaneously and propagate through the network under conditions of near-criticality (Rabuffo et al., 2020). The largest neuronal cascades produce short-lived but robust co-fluctuations at pairs of regions across the brain, thus contributing to the organization of the slowly evolving spontaneous fluctuations in brain dynamics at rest. The introduction of extrinsic or endogenous noise sources in the framework of exact neural mass models is possible in terms of (pseudo)-cumulants expansion in Tyulkina et al. (2018) and Goldobin et al. (2021).

In this paper, we have built brain network models for a cohort of 20 healthy subjects and 15 epileptic patients, implementing for each brain region the neural mass model developed by Montbrió et al. (2015). As paradigms for testing the spatiotemporal organization, we have systematically simulated the individual seizure-like propagation patterns, looking for the role played by the individual structural topologies in determining the recruitment mechanisms. Specific attention has been devoted to the analogies and differences among the self-emergent dynamics in healthy and epilepsy-affected subjects. Furthermore, for epileptic patients, we have validated the model against the presurgical SEEG data and the standard-of-care clinical evaluation. More specifically the Methods section is devoted to the description of the implemented model and the applied methods. In section Healthy Subjects are reported the results specific for healthy subjects, while in section Epileptic Patients is reported a detailed analysis performed on epileptic patients. Finally, a discussion on the presented results is reported in section Discussion.

## 2. Methods

### 2.1. Network Model

The membrane potential dynamics of the i-th quadratic integrate-and-fire (QIF) neuron in a network of size *N* can be written as

(1) $$\begin{array}{l} \tau_{\text{m}}{\overset{∙}{V}}_{i}=V_{i}^{2}\left(t\right)+\eta_{i}+I_{\text{B}}+I_{\text{S}}\left(t\right)+\tau_{\text{m}}\frac{1}{N}\overset{N}{\underset{j=1}{\sum }}{\tilde{J}}_{ij}\left(t\right)S_{j}\left(t\right)\;,\\ \;i=1,\ldots ,N \end{array}$$

where τ_m_ = 20 ms is the membrane time constant and ${\tilde{J}}_{ij}\left(t\right)$ the strength of the direct synapse from neuron *j* to *i* that we assume to be constant and all identical, i.e., ${\tilde{J}}_{ij}\left(t\right)=J$. The sign of *J* determines if the neuron is excitatory (*J* > 0) or inhibitory (*J* < 0); in the following, we will consider only excitatory neurons. Moreover, η_*i*_ represents the neuronal excitability, *I*_B_ a constant background DC current (in the following we assume *I*_B_ = 0), *I*_S_(*t*) an external stimulus, and the last term on the right-hand side the synaptic current due to the recurrent connections with the pre synaptic neurons. For instantaneous post synaptic potentials (corresponding to δ-spikes), the neural activity *S*_*j*_(*t*) of neuron *j* reads as

(2) $$\begin{array}{l} S_{j}\left(t\right)=\underset{t_{j}\left(k\right)<t}{\sum }\delta \left(t-t_{j}\left(k\right)\right), \end{array}$$

where *S*_*j*_(*t*) is the spike train produced by the *j*-th neuron and *t*_*j*_(*k*) denotes the *k*-th spike time in such sequence. We have considered a fully coupled network without autapses, therefore, the post-synaptic current will be the same for each neuron.

In the absence of synaptic input, external stimuli, and *I*_B_ = 0, the QIF neuron exhibits two possible dynamics, depending on the sign of η_*i*_. For negative η_*i*_, the neuron is excitable and for any initial condition $V_{i}\left(0\right)<\sqrt{-\eta_{i}}$, it reaches asymptotically the resting value $-\sqrt{-\eta_{i}}$. On the other hand, for initial values larger than the excitability threshold, $V_{i}\left(0\right)>\sqrt{-\eta_{i}}$, the membrane potential grows unbounded and a reset mechanism has to be introduced to describe the spiking behavior of a neuron. Whenever *V*_*i*_(*t*) reaches a threshold value *V*_p_, the neuron *i* delivers a spike and its membrane potential is reset to *V*_r_, for the QIF neuron *V*_p_ = −*V*_r_ = ∞. For positive η_*i*_, the neuron is supra-threshold and it delivers a regular train of spikes with frequency $\nu_{0}=\sqrt{\eta_{i}}/\pi$.

### 2.2. Neural Mass Model

For the heterogeneous QIF network with instantaneous synapses (Equations 1, 2), an exact neural mass model has been derived by Montbrió et al. (2015). The analytic derivation is possible for QIF spiking networks using the Ott-Antonsen Ansatz (Ott and Antonsen, 2008) applicable to phase-oscillator networks, whenever the natural frequencies are distributed according to a Lorentzian distribution. In the case of the QIF network, this corresponds to a distribution of the excitabilities {η_*i*_} given by,

(3) $$\begin{array}{l} g\left(\eta \right)=\frac{1}{\pi }\frac{\Delta }{{\left(\eta -\bar{\eta }\right)}^{2}+\Delta^{2}}\;, \end{array}$$

which is centered in $\bar{\eta }$ and has half width at half maximum (HWHM) Δ (Δ = 1 throughout this study). In particular, this neural mass model allows for an exact macroscopic description of the population dynamics, in the thermodynamic limit *N* → ∞, in terms of only two collective variables, namely the mean membrane voltage potential *v*(*t*) and the instantaneous population rate *r*(*t*), as follows

(4a) $$\begin{array}{ll} \tau_{\text{m}}ṙ\left(t\right) & =\frac{\Delta }{\tau_{\text{m}}\pi }+2r\left(t\right)v\left(t\right) \end{array}$$

(4b) $$\begin{array}{ll} \tau_{\text{m}}\overset{∙}{v}\left(t\right) & =v^{2}\left(t\right)+\bar{\eta }+I_{\text{B}}+I_{\text{S}}\left(t\right)-{\left[\pi \tau_{\text{m}}r\left(t\right)\right]}^{2}+\tau_{\text{m}}\tilde{J}\left(t\right)r\left(t\right)\;; \end{array}$$

where the synaptic strength is assumed to be identical for all neurons and instantaneous synapses in absence of plasticity $\tilde{J}\left(t\right)=J$. However, by including a dynamical evolution for the synapses and, therefore, additional collective variables, this neural mass model can be extended to any generic post synaptic potential, as shown in e.g., Devalle et al. (2017) for exponential synapses or Coombes and Byrne (2019) for conductance-based synapses with α-function profile. In the following, we will consider an extension of the original model (Equations 4) to a complex topology, where multiple nodes interact with each other. By considering instantaneous post-synaptic potentials and neglecting synaptic features, we then focus on the role played by the topology in enhancing the emergence of complex dynamics.

### 2.3. Multipopulation Neural Mass Model

The neural mass model can be easily extended to account for multiple interconnected neuronal populations *N*_pop_. In the following, we consider personalized brain models derived from structural data of Magnetic Resonance Imaging (MRI), Diffusion Weighted Imaging (DWI) and Diffusion Tensor Imaging (DTI), thus implementing different structural connectivity matrices for healthy subjects and epileptic patients. For healthy subjects cortical and volumetric parcellations were performed using the Automatic Anatomical Atlas 1 (AAL1) (Tzourio-Mazoyer et al., 2002) with *N*_pop_ = 90 brain regions: each region will be described in terms of the presented neural mass model. For epileptic subjects, cortical and volumetric parcellations were performed using the Desikan-Killiany atlas with 70 cortical regions and 17 subcortical regions (Desikan et al., 2006) (one more empty region is added in the construction of the structural connectivity for symmetry). In this case, the structural connectivity matrix is composed, for each patient with epileptic, by 88 nodes equipped with the presented region-specific neural mass model capable of demonstrating epileptiform discharges.

The corresponding multi-population neural mass model can be straightforwardly written as

(5a) $$\begin{array}{ll} \tau_{\text{m}}\overset{∙}{r_{k}} & =\frac{\Delta_{k}}{\tau_{\text{m}}\pi }+2r_{k}\left(t\right)v_{k}\left(t\right)\;k=1,2,\ldots ,N_{\text{pop}} \end{array}$$

(5b) $$\begin{array}{ll} \tau_{\text{m}}{\overset{∙}{v}}_{k} & =v_{k}^{2}\left(t\right)+{\bar{\eta }}^{\left(k\right)}+I_{\text{B}}+{I_{\text{S}}}^{\left(k\right)}\left(t\right)-{\left(\pi \tau_{\text{m}}r_{k}\left(t\right)\right)}^{2}+\tau_{\text{m}}\overset{N_{\text{pop}}}{\underset{l=1}{\sum }}J_{kl}r_{l}\left(t\right), \end{array}$$

where {*J*_*kl*_} is the connectivity matrix, representing the synaptic weights among the populations. Diagonal entries *J*_*kk*_ denote intra-population and non-diagonal entries *J*_*kl*_, *k* ≠ *l* inter-population connections. In this study, we have assumed that the neurons are globally coupled both at the intra- and inter- population levels, hence removing the dependency on the neuron indices.

The connectivity matrix entries *J*_*kl*_ are determined *via* a second matrix $\left\{{\tilde{J}}_{kl}\right\}$, which represents the topology extracted from empirical DTI. The values of $\left\{{\tilde{J}}_{kl}\right\}$ are normalized in the range [0, 1] via rescaling with the maximal entry value, and have ${\tilde{J}}_{kk}=0$ on the diagonal. To account for strong intra-coupling (recurrent synapses) and intermediate inter-coupling, we choose the entries of each structural connectivity as

(6) $$\begin{array}{l} J_{kl}=\sigma \{\begin{array}{cc} 5{\tilde{\;J}}_{kl} & \text{ if }k\neq l\\ 20 & \text{ if }k=l, \end{array} \end{array}$$

where σ is a rescaling factor common to all synapses, that we assume to be constant and equal to 1, apart from few cases where we investigate the dependence on the synaptic weights. Hence, the synaptic weights for *k* ≠ *l* are in the range *J*_*kl*_ ∈ [0, 5], while the intra coupling is set to *J*_*kk*_ = 20 (apart from when specified otherwise). This choice of the rescaling factor ensures that the single brain region finds itself in a bistable regime, thus being able to switch from a low-activity to a high-activity regime. The time-dependent stimulus current $I_{S}^{\left(k\right)}$ is population-specific, and a single population at a time is generally stimulated during a numerical experiment. The applied stimulus $I_{S}^{\left(k\right)}$ consists of a rectangular pulse of amplitude *I*_*S*_ and duration *t*_*I*_; the dependence on these parameters is studied in this paper to support the generality of the results.

### 2.4. Topologies

As the first set of data, we have selected 20 diffusion-weighted MRI connectomes of healthy subjects (mean age 33 years, SD 5.7 years, 10 females, 2 left-handed) that participated in a study on schizophrenia as a control group (Melicher et al., 2015). Throughout the study we refer to the healthy subjects as H1–H20. All subjects were recruited *via* local advertisements and had none of the following conditions: Personal lifetime history of any psychiatric disorder or substance abuse established by the Mini-International Neuropsychiatric Interview (MINI) (Lecrubier et al., 1997), and any psychotic disorder in first or second-degree relatives. Further exclusion criteria included current neurological disorders, lifetime history of seizures or head injury with altered consciousness, intracranial hemorrhage, neurological sequelae, history of intellectual disability, history of substance dependence, and any contraindication for MRI scanning.

The scans were performed on a 3T Siemens scanner in the Institute of Clinical and Experimental Medicine in Prague, employing a spin-echo EPI sequence with 30 diffusion gradient directions, *TR* = 8, 300 ms, *TE* = 84 ms, 2 × 2 × 2 *mm*^3^ voxel size, and *b*-value 900 *s*/*mm*^2^. The diffusion-weighted images (DWI) were analyzed using the Tract-Based Spatial Statistics (TBSS) (Smith et al., 2006), part of FMRIB Software Library (FSL) (Smith et al., 2004). Image conversion from DICOM to NIfTI format was accomplished using dcm2nii. With FMRIB's Diffusion Toolbox (FDT), the fractional anisotropy (FA) images were created by fitting a tensor model to the raw diffusion data and then, using the Brain Extraction Tool (BET) (Smith, 2002), brain-extracted. FA identifies the degree of anisotropy of a diffusion process, and it is a measure often used in diffusion imaging where it is thought to reflect fiber density, axonal diameter, and myelination in white matter. A value of zero means that diffusion is isotropic, i.e., it is unrestricted (or equally restricted) in all directions, while a value of one means that diffusion occurs only along one axis and is fully restricted along all other directions. Subsequently, the FA images were transformed into a common space by nonlinear registration IRTK (Rueckert et al., 1999). A mean FA skeleton, representing the centers of all tracts common to the group, was obtained from the thinned mean FA image. All FA data were projected onto this skeleton. The resulting data was fed into voxel-wise cross-subject statistics. Prior to analysis in SPM, the FA maps were converted from NIfTI format to Analyze.

The brains were segregated into 90 brain areas according to the AAL1 (Tzourio-Mazoyer et al., 2002). The anatomical names of the brain areas for each index *k* are shown in the Supplementary Table 1. In each brain network, one AAL brain area corresponds to a node of the network. The weights between the nodes were estimated through the measurement of the preferred diffusion directions, given by a set of *n*_*s*_ = 5, 000 streamlines for each voxel. The streamlines are hypothesized to correlate with the white-matter tracts. The ratio of streamlines connecting area *l* and area *k* is given by the probability coefficient *p*_*lk*_. Then, the adjacency matrix *J*_*kl*_ is constructed from this probability coefficient. The DTI processing pipeline has been adopted from Cabral et al. (2013).

Besides the healthy connectomes, we selected 15 connectomes (9 females, 6 males, mean age 33.4, range 22–56) of patients with different types of partial epilepsy that underwent a presurgical evaluation. The scans were performed at the Centre de Résonance Magnétique et Biologique et Médicale (Faculté de Médecine de la Timone) in Marseille. Throughout the study, we refer to the epileptic patients as E1–E15. dMRI images were acquired on a Siemens Magnetom Verio 3T MR-scanner using a DTI-MR sequence with an angular gradient set of 64 directions, *TR* = 10, 700 ms, *TE* = 95 ms, 2 × 2 × 2 *mm*^3^ voxel size, 70 slices, and *b*-value 1, 000 *s*/*mm*^2^.

The data processing pipeline (Schirner et al., 2015; Proix et al., 2016) made use of various tools such as FreeSurfer (Fischl, 2012), FSL (Jenkinson et al., 2012), MRtrix3 (Tournier, 2010), and Remesher (Fuhrmann et al., 2010), to reconstruct the individual cortical surface and large-scale connectivity. The surface was reconstructed using 20,000 vertices. Cortical and volumetric parcellations were performed using the Desikan-Killiany atlas with 70 cortical regions and 17 subcortical regions (Desikan et al., 2006). The final atlas consists of 88 regions since one more empty region is added in the construction of the structural connectivity for symmetry. After correction of the diffusion data for eddy-currents and head motions using eddy-correct FSL functions, the fiber orientation was estimated using constrained spherical deconvolution (Tournier et al., 2007) and improved with anatomically constrained tractography (Smith et al., 2012). For tractography, 2.5 × 10^6^ fibers were used and, for correction, spherical-deconvolution informed filtering of tractograms (Smith et al., 2013) was applied. Summing track counts over each region of the parcellation yielded the adjacency matrix. In this study, the AAL2 was employed for brain segregation leading to 88 brain areas for each patient, as shown in Supplementary Table 2.

### 2.5. EEG and SEEG Data

All 15 drug-resistant patients, mentioned in the previous section, affected by different types of partial epilepsy accounting for different EZ localizations, underwent a presurgical evaluation (as shown in Supplementary Tables 3, 4). For each patient, a first non invasive evaluation procedure is foreseen, which comprises of the patient clinical record, neurological examinations, PET, and EEG along with video monitoring. Following this evaluation, potential EZs are identified by the clinicians. Further elaboration on the EZ is done in a second, invasive phase, which consists of positioning SEEG electrodes in or close to the suspected regions. These electrodes are equipped with 10–15 contacts that are 1.5 mm apart. Each contact has a length of 2 mm and measures 0.8 mm in diameter. Recordings were obtained using a 128 channel DeltamedTM system with a 256 Hz sampling rate and band-pass filtered between 0.16 and 97 Hz by a hardware filter. Precise electrode positioning was performed by either a computerized tomography or MRI scan after implanting the electrodes. All patients showed seizures in the SEEG, starting in one or several localized areas (EZ), before recruiting distant regions, identified as the PZ. It is worth noticing that, among the operated patients, four of them showed a worthwhile improvement but without resulting completely seizure-free since surgery (Engel's score III), while two resulted almost seizure-free, showing rare disabling seizures since surgery (Engel's score II), thus suggesting that the EZ was correctly identified in a subset of patients only.

Two methods were used for the identification of the PZ (as shown in Supplementary Table 4). First, the clinicians evaluated the PZs subjectively based on of the EEG and SEEG recordings gathered throughout the two-step procedure (non invasive and invasive). Second, the PZs were identified automatically based on the SEEG recordings: For each patient, all seizures were isolated in the SEEG time series. The bipolar SEEG was considered (between pairs of electrode contacts) and filtered between 1 and 50 Hz using a Butterworth band-pass filter. An area was defined as a PZ if its electrodes detected at least 30% of the maximum signal energy over all contacts, and if it was not in the EZ. In the following, we call the PZs identified by the subjective evaluation of clinicians PZ_Clin_ and the PZs identified through SEEG data PZ_SEEG_.

### 2.6. Network Measures

The topological properties of a network can be examined by using different graph measures that are provided by the general framework of graph theory. These graph metrics can be classified in terms of measures that cover three main aspects of the topology: segregation, integration, and centrality. The segregation accounts for the specialized processes that occur inside a restricted group of brain regions, usually densely connected, and it eventually reveals the presence of a dense neighborhood around a node, which results to be fundamental for the generation of clusters and cliques capable to share specialized information. Among the possible measures of segregation, we have considered the *clustering coefficient*, which gives the fraction of triangles around a node and it is equivalent to the fraction of neighbors of the node that are neighbors of each other as well. In particular, the *average clustering coefficient* *C* of a network gives the fraction of closed triplets over the number of all open and closed triplets, where a triplet consists of three nodes with either two edges (open triplet) or three edges (closed triplet). The *weighted clustering coefficient* $c_{i}^{w}$ (Barrat et al., 2004) considers the weights of its neighbors:

(7) $$\begin{array}{l} c_{i}^{w}=\frac{1}{s_{i}\left(k_{i}-1\right)}\underset{j,h}{\sum }\frac{w_{ij}+w_{ih}}{2}a_{ij}a_{ih}a_{jh}, \end{array}$$

where *s*_*i*_ is the node strength (to be defined below), *k*_*i*_ the node degree, *w*_*ij*_ the weight of the link, and *a*_*ij*_ is 1 if the link *i*→*j* exists and 0 if node *i* and *j* are not connected. The *average weighted clustering coefficient* *C*_*W*_ is the mean of all weighted clustering coefficients: $C_{W}=\frac{1}{N}\underset{i}{\sum }c_{i}$.

The measures of integration refer to the capacity of the network to rapidly combine specialized information from not nearby, distributed regions. Integration measures are based on the concept of communication paths and path lengths, which estimate the unique sequence of nodes and links that can carry the transmission flow of information between pairs of brain regions. The *shortest path* *d*_*ij*_ between two nodes is the path with the least number of links. The *average shortest path length* of node *i* of a graph *G* is the mean of all shortest paths from node *i* to all other nodes of the network: $L\left(G,i\right)=\frac{1}{N-1}\underset{j\in \mathbb{N},j\neq i}{\sum }d_{ij}$. The *average shortest path length* of all nodes is the mean of all shortest paths (Boccaletti et al., 2006): $L\left(G\right)=\frac{1}{N-1}\underset{i,j\in \mathbb{N},i\neq j}{\sum }d_{ij}$. In a weighted network, distance and weight have a reciprocal relation. If a weight between two adjacent nodes is doubled, their shortest path is cut by half: $L\left(G\right)=\frac{1}{N-1}\underset{i,j\in \mathbb{N},i\neq j}{\sum }\frac{d_{ij}}{w_{ij}}$.

Centrality refers to the importance of network nodes and edges for network functioning. The most intuitive index of centrality is the node degree, which gives the number of links connected to the node; for this measure, connection weights are ignored in calculations. In this study, we employ the network measure *node strength* *s*_*i*_, which corresponds to the weighted node degree of node *i* and equals the sum of all its weights: $s_{i}=\underset{j\in \mathbb{N}}{\sum }w_{ij}$. Accordingly, the *average node strength* *S* corresponds to the mean of all node strengths $S=\frac{1}{N}\underset{i}{\sum }s_{i}$. All finite networks have a finite number of shortest paths *d*(*i, j*) between any pair of nodes *i*, *j*. The *betweenness centrality* *c*_*B*_(*s*) of node *s* is equal to all pairs of shortest paths that pass through *s* divided by the number of all shortest paths in the network: $c_{B}\left(s\right)=\underset{i,j\in \mathbb{N}}{\sum }\frac{d\left(i,j|s\right)}{d\left(i,j\right)}$. For the *weighted betweenness centrality*, the *weighted shorted paths* are used.

### 2.7. Spectrogram Estimation

To generate the spectrograms, the *signal* package, part of the SciPy library (Virtanen et al., 2020), is used. The subroutine *stft* (short-time Fourier transform, STFT) generates Fourier transforms F[s(t)](t,f) of a signal *s*(*t*) within a running time window of length Δ*T*_win_ at time *t*. The STFT is performed using overlapping windows (95% overlap) throughout this study. The window length is set to Δ*T*_win_ = 0.2 s, leading to a sufficiently fine resolution in time and frequency. The colors in the spectrograms code the normalized power spectral density $|F\left[v_{k}\left(t\right)\right]\left(t,f\right){|}^{2}/\left(\max|F\left[v_{k}\left(t\right)\right]\left(t,f\right){|}^{2}\right)$ obtained from voltage signals *v*_*k*_ of different populations. For a better visibility, a log10 scale is used and values <10^−2^ are set to 10^−2^. Fourier transforms of the individual voltage signals *v*_*k*_ of different populations are first calculated giving rise to individual power spectral densities that are subsequently averaged over the populations to obtain the data *f*_*avg*_ shown in **Figures 2**, **9**. Finally, the spectrograms are shifted to the right by 0.1 s to preserve causality in correspondence of the stimulus onset.

## 3. Results

The epileptic attractor is commonly described in terms of a self-sustained limit cycle that comes from the destabilization of the physiological activity while multiple types of transitions allow for the accessibility of seizure activity, status epilepticus, and depolarization block, that coexist, as verified experimentally in El Houssaini et al. (2020). The single-population firing rate (Equation 4) shows, in the absence of forcing, only fixed points as attractors. As it will become clear in the following section, a stable node and a stable focus are observable, separated by a bistability region between a high- and a low-activity state, whose boundaries are the locus of a saddle-node bifurcation (for more details see Montbrió et al., 2015). In this context are not observable self-sustained oscillations but only damped oscillations at the macroscopic level that reflect the oscillatory decay to the stable fixed point. This oscillatory decay will be considered as the representative of a seizure-like event, not being able to observe a stable limit cycle to describe the emergence of a fully developed seizure, as shown in other phenomenological mathematical models (Jirsa et al., 2014; Chizhov et al., 2018), commonly used to describe a detailed taxonomy of seizures. In particular, seizure-like events will be used as a paradigm to investigate the propagation of seizure-like activity in the network. A detailed comparison with the taxonomy of seizures described by other phenomenological models (Jirsa et al., 2014; Saggio et al., 2017; Chizhov et al., 2018) and the possible extension of the single-population firing rate (Equation 4) to show self-emergent periodic and bursting dynamics at the macroscopic level is reported in section 1 in the Supplementary Material.

### 3.1. Healthy Subjects

#### 3.1.1. Phase and Bifurcation Diagrams

The analysis of the single-population firing rate Equations (4), performed in Montbrió et al. (2015), has revealed that there are three distinct regions, when considering the phase diagram of the system as a function of the external drive $\bar{\eta }$ and synaptic weight *J*, in absence of time-dependent forcing [*I*(*t*) = 0]: (1) a single stable node equilibrium corresponding to a low-activity state, (2) a single stable focus (spiral) generally corresponding to a high-activity state, and (3) a region of bistability between low and high firing rates. In particular, in the region where the stable focus is observable, the system undergoes damped oscillatory motion toward this fixed point. The presence of damped oscillations at the macroscopic level reflects the transitory synchronous firing of a fraction of the neurons in the ensemble. While this behavior is common in network models of spiking neurons, it is not captured by traditional firing-rate models (Schaffer et al., 2013; Devalle et al., 2017; Taher et al., 2020).

**Figure 1:**
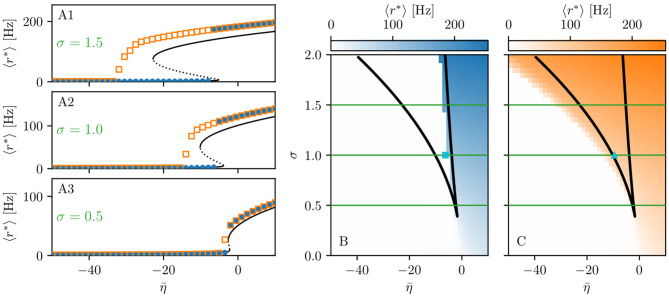
Phase and bifurcation diagrams for subject H1. **(A1–A3)** Equilibrium firing rates 〈r^*^〉 vs. $\bar{\eta }$ for the up sweep (blue dots) and down-sweep (orange squares). For each $\bar{\eta }\in \left[-50,10\right]$ in steps of $\Delta \bar{\eta }=1.5$, the system is initialized using the final state of the previous run and evolves for 2 s after which the average network firing rate in the equilibrium state is determined. Different panels correspond to different σ values: σ = 1.5 **(A1)**, σ = 1 **(A2)**, and σ = 0.5 **(A3)**. The solid (dashed) black line corresponds to the stable (unstable) equilibria in the single-node case. Maps of regimes as a function of σ and $\bar{\eta }$ showing the network average 〈r^*^〉 color-coded for up- **(B)** and down-sweep **(C)**, obtained by following the same procedure as in **(A1–A3)** for σ ∈ [0, 2] in steps of Δσ = 0.05. The black line indicates the single-node map of regimes like in Montbrió et al. (2015). In panels **(B,C)** the cyan square and triangle mark $\bar{\eta }=-6.3,-9.54$, respectively. Parameter values: *N*_pop_ = 90, τ_m_ = 20 ms, Δ = 1, *J*_*kk*_ = 20, $J_{kl}=5{\tilde{J}}_{kl},\;\forall k\neq l$.

When considering the multipopulation neural mass model (5) with homogeneously set ${\bar{\eta }}^{\left(k\right)}=\bar{\eta }$, the corresponding phase diagram (shown in Figures 1B,C) is qualitatively the same as the one shown in Figure 1 in Montbrió et al. (2015), since the same attractors are observable. In the original model, these attractors are single-population states, while they reflect multipopulation states in the present case. Single-population low-activity (LA) and high-activity (HA) states translate into network LA and HA states. In the former, all populations have low, in the latter high firing rates. We observe that the single-population bistability accurately reflects the hysteretic transition in the network when changing $\bar{\eta }$. In the following, we will address how this relation between single-node and multipopulation phase diagram occurs.

The network bifurcation diagrams shown in Figures 1A1–A3 for increasing σ values are obtained by performing an adiabatic analysis along with two different protocols such as up sweep and down-sweep. Following the up-sweep protocol, the state variables *r*_*k*_, *v*_*k*_ of the system are initialized at $\bar{\eta }=-50$ with the values *r*_*k*_ = 0, *v*_*k*_ = 0; then the excitability is increased in steps $\Delta \bar{\eta }=1.5$ until the maximal value $\bar{\eta }=10$ is reached. At each step, the initial conditions for mean firing rates and mean membrane potentials correspond to the final state obtained for the previous $\bar{\eta }$ value. Note, that the average firing rate increases for increasing $\bar{\eta }$ values, both for the single node and the network. Once the maximum $\bar{\eta }$ value is reached, the reverse procedure is performed, thus following the down-sweep protocol. This time the initial conditions correspond to the high-activity state at $\bar{\eta }=10$, while the excitability is adiabatically decreased in steps $\Delta \bar{\eta }=1.5$ until a low-activity state at $\bar{\eta }=-50$ is approached. For both protocols, the investigation of the nature of the dynamics emerging at each $\bar{\eta }$-step is done by using the same procedure: the system is simulated for a transient time *T* = 2 s until it has reached an equilibrium state. At this time, the firing rate averaged over-all populations 〈r^*^〉 is calculated and the next $\bar{\eta }$ iteration is started, using this final state as initial conditions.

The transition from LA to HA network dynamics is hysteretic: the system does not follow the same path during the up sweep and the down-sweep protocol. When the system is initialized in the low activity regime, it remains there until a critical excitability value ${\bar{\eta }}_{\text{HA}}$ is reached. For further increase of the excitability, the average firing rate exhibits a rapid jump to higher values. However, when the system is initialized in the high-activity regime, this regime survives for a large $\bar{\eta }$ interval until it collapses toward a low-activity state at $\bar{\eta }<{\bar{\eta }}_{\text{LA}}$, where ${\bar{\eta }}_{\text{LA}}<{\bar{\eta }}_{\text{HA}}$. There is a considerable difference between the critical excitability values required to lead the system to a high-activity or a low-activity regime and the difference increases for increasing coupling strength σ. While the up-sweep protocol (blue dots) is well approximated by the bifurcation diagram of the single population, represented in Figures 1A1–A3 by the black (dashed and continuous) curve, this is no more true for the down-sweep protocol, where the coupling plays a role in determining the transition at the multipopulation level (orange squares). This results in different phase diagrams for the two protocols: the maps of regimes are dominated by the low-activity (high-activity) state when following the up-sweep (down-sweep) protocol. Merging these results, we observe that the region of bistability between LA and HA network dynamics is still identifiable by the original boundaries found for the single population in Montbrió et al. (2015) (black curve in Figures 1B,C), even though, for the multipopulation system, the region is wider.

We can make further use of the single-population bifurcation diagram to understand the hysteretic transition of the multipopulation model in more detail. First of all, the weight matrix {*J*_*kl*_} has its largest components on the diagonal (*J*_*kk*_ = 20), reflecting recurrent synapses. This means that synaptic inter coupling plays a minor role, as long as the firing rates of the adjacent populations are small. During the up-sweep protocol, this condition is fulfilled, as all populations are initialized in a low activity regime. Under these conditions, the dynamics of all nodes are rendered identical and equal, approximately, to the single population dynamics. Consequently, the single-population LA branch describes the multipopulation LA behavior (in terms of 〈r^*^〉) accurately as a function of $\bar{\eta }$. Second, as soon as the single-population LA state vanishes for large enough $\bar{\eta }>{\bar{\eta }}_{\text{HA}}$, the individual nodes of the multipopulation system all transit to the HA state.

In this HA regime, deviations of the network bifurcation diagram with respect to the single-population curve are observed. The populations in the system have large firing rates, such that the inter-coupling becomes a relevant contribution to the total current on each node. This explains why the LA branch of the network is located at higher firing rates with respect to the black single-population curve: The populations in the network behave, approximately, as decoupled, irrespectively of being subject, in the HA regime, to an additional current due to the inter-coupling. This effectively shifts the single-population bifurcation diagram toward smaller $\bar{\eta }$. Moreover, this shift occurs for each population individually, depending on the matrix {*J*_*kl*_}. During the down-sweep protocol, due to the population-dependent shift, the HA population states vanish at different values of $\bar{\eta }$. Accordingly, whenever this occurs, the network average 〈r^*^〉 decreases by a small amount, such that the network LA state is reached *via* various intermediate states. We can infer, using the same type of argument, that single-population LA states disappear for increasing $\bar{\eta }$ in a region around ${\bar{\eta }}_{\text{HA}}$. They are not observed in this study, due to the nature of the up-sweep protocol and the initialization procedure of *r*_*k*_, *v*_*k*_.

From the reversed viewpoint, we can hypothesize, that stable single-population HA states may take form near ${\bar{\eta }}_{\text{LA}}$ for increasing $\bar{\eta }$ and stable LA states for decreasing $\bar{\eta }$ near ${\bar{\eta }}_{\text{HA}}$. This implies that the network possesses complex multistability between many network states in the region ${\bar{\eta }}_{\text{LA}}<\bar{\eta }<{\bar{\eta }}_{\text{HA}}$. For these states, the existence of LA and HA states of individual populations are interdependent: Whether or not any given population can be in the LA or HA state is conditioned by the LA-HA configuration of all other populations. This not only demonstrates how multistability emerges in the multipopulation system but also influences the response of the network towards transient input in such a setting. Most importantly, if such an input recruits one population into high activity, other populations might follow, leading to a cascade of recruitments.

#### 3.1.2. Seizure-Like Recruitment in Dependence of Perturbation Site and $\bar{\eta }$

**Figure 2:**
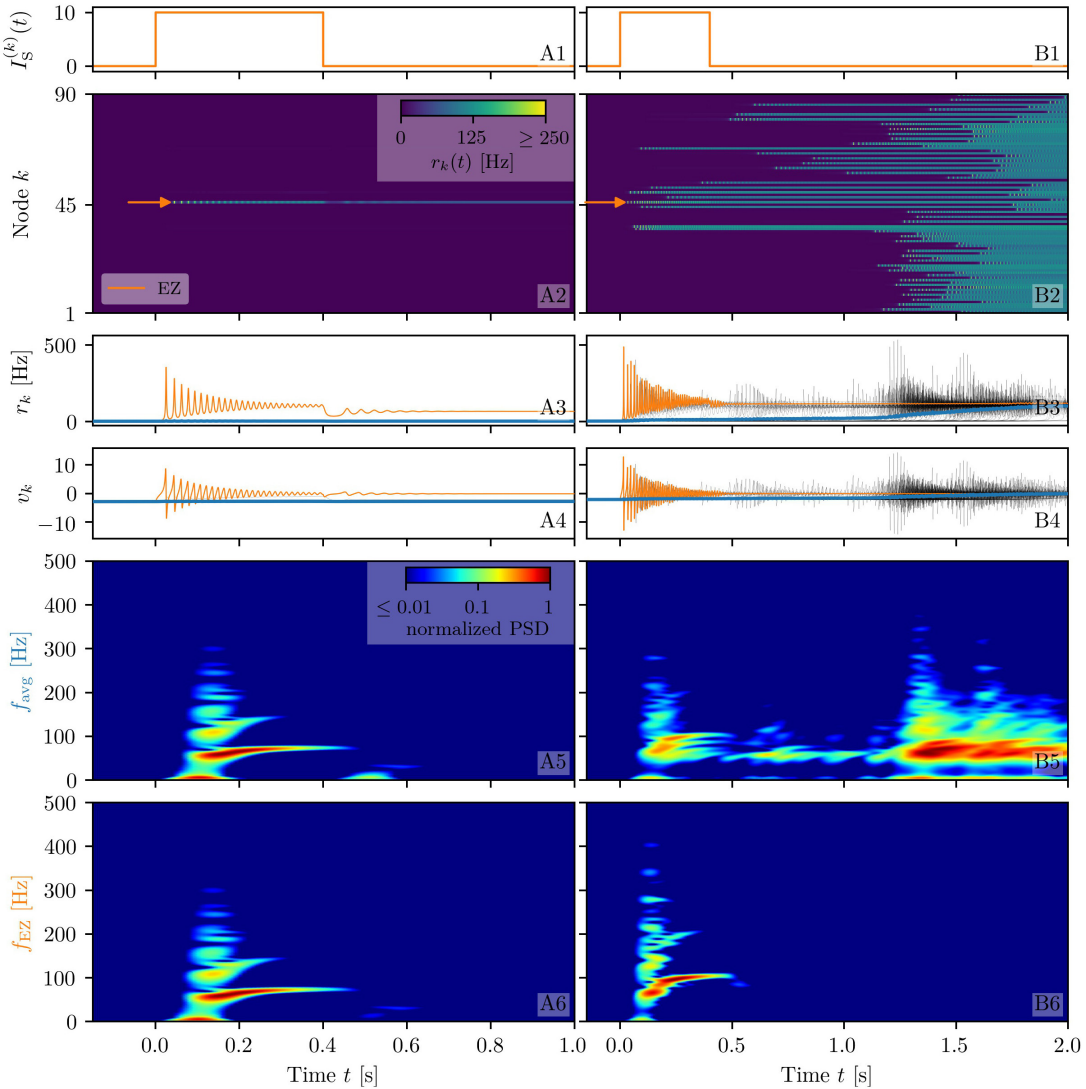
Spectrograms of mean membrane potentials for healthy subject H1. **(A1, B1)** Stimulation current $I_{S}^{\left(k\right)}$, **(A3, B3)** population firing rates *r*_*k*_ , and **(A4, B4)** mean membrane potentials *v*_*k*_ for the EZ (orange) and other populations (black). The blue curves show the network average firing rate and membrane potential. Non-stimulated node dynamics are plotted as transparent gray curves: some of the nodes adapt their voltage to the stimulation of the EZ and change during stimulation. **(A2, B2)** Space-time plots of the population firing rates *r*_*k*_, color-coding the value of the firing rate of each node, as a function of time. **(A5, B5)** Spectrogram of the network average membrane potential and **(A6, B6)** of the *v*_*k*_ of the EZ. Column A shows an asymptomatic seizure-like event for ${\bar{\eta }}^{\left(k\right)}=\bar{\eta }=-9.54$, column B shows a generalized seizure-like event for ${\bar{\eta }}^{\left(k\right)}=\bar{\eta }=-6.3$. In both cases, the EZ node 46 is stimulated. Parameter values: *N*_pop_ = 90, τ_m_ = 20 ms, Δ = 1, *J*_*kk*_ = 20, σ = 1, $J_{kl}=5{\tilde{J}}_{kl},\;\forall k\neq l$.

To analyze the response of the multipopulation system to transient current, we stimulate one population with a step function *I*_*S*_(*t*) of amplitude *I*_*S*_ = 10 and duration *t*_*I*_ = 0.4 s. By setting $\bar{\eta }=-9.54$, the system is placed in the multistable regime (cyan triangle in Figure 1C), but, due to the low $\bar{\eta }$ value, it only allows for LA-HA configurations with most of the populations in LA. The stimulation with an external current *I*_*S*_(*t*) allows the system to reach a configuration with more populations in the HA. This corresponds to choosing equivalently, in the model, a higher excitability value for the single node such that it crosses the bistability region, thus reaching the HA regime. We start by initializing all nodes in the low-activity state and stimulating a single node (Figure 2A). During the stimulation (Figure 2A1), the stable states of the network change, due to the strong additional current (Figure 2A2). More specifically, the initial equilibrium vanishes and a new focus equilibrium of the system appears as the only stable network state. This focus is characterized by an LA-HA configuration for which only the stimulated node finds itself in HA while the rest remains in the LA regime; the focus is approached *via* damped oscillations in the time interval 0 < *t* < 0.4 s (Figures 2A3,A4). Due to the multistability in absence of stimulation, an identical LA-HA configuration exists. Thus, when the current is removed, the system is able to maintain the LA-HA configuration. However, the position of the focus equilibrium is shifted in absence of the transient input and is reached again, *via* damped oscillations for *t* > 0.4 s.

When the perturbation of a single node has no consequences on the dynamics of the other populations, as shown in Figures 2A2–A4, we are in the presence of an *asymptomatic seizure-like event*, where the activity is limited to the EZ represented by the stimulated node and no propagation takes place. For higher excitability values ($\bar{\eta }=-6.3$, marked by a cyan rectangle in Figure 1B), the perturbation of a single node gives rise to different response dynamics. In this case, other brain areas are “recruited” and not only the perturbed node but also other populations reach the high-activity regime by showing damped oscillations (see Figures 2B2–B4). In terms of pathological activity, the seizure-like event originates in the EZ (as a result of the stimulation) and propagates to the PZ, identified by the other regions which rapidly propagate the oscillatory activity. The recruitment of the regions in the PZ can happen either by independent activation of the single areas or by activating multiple areas at the same time, in a *domino-like* effect (Creaser et al., 2020), until the propagation involves almost all populations (*generalized seizure-like event*).

The transition of a single population to the HA regime, upon stimulus onset, is characterized by a transient activity in the α−β band (10–14 Hz) and a sustained activity in the γ band (40–80 Hz), present throughout the stimulation, as shown in Figures 2A5,A6. In this study, the spectrograms show time-varying power spectral densities (PSD) of the mean membrane potentials averaged over the network (Figure 2A5) and for the single stimulated population (Figure 2A6). When more populations are recruited at higher excitability values, in addition to the former activity, it is possible to observe γ activity at higher frequencies (as shown in Figures 2B5,B6). High-frequency oscillations, between 80 and 500 Hz, can be recorded with EEG and reflect the seizure-generating capability of the underlying tissue, thus being used as markers of the EZ (Jacobs et al., 2012). Moreover, even for the generalized seizure-like case, the low-frequency band activity is evoked whenever a brain area gets recruited, leading to a sustained signal in the time interval 1.1s < *t* < 1.8 *s*, where a majority of the populations approaches the HA state. Similar results have been obtained for all the other investigated subjects (results not shown).

**Figure 3:**
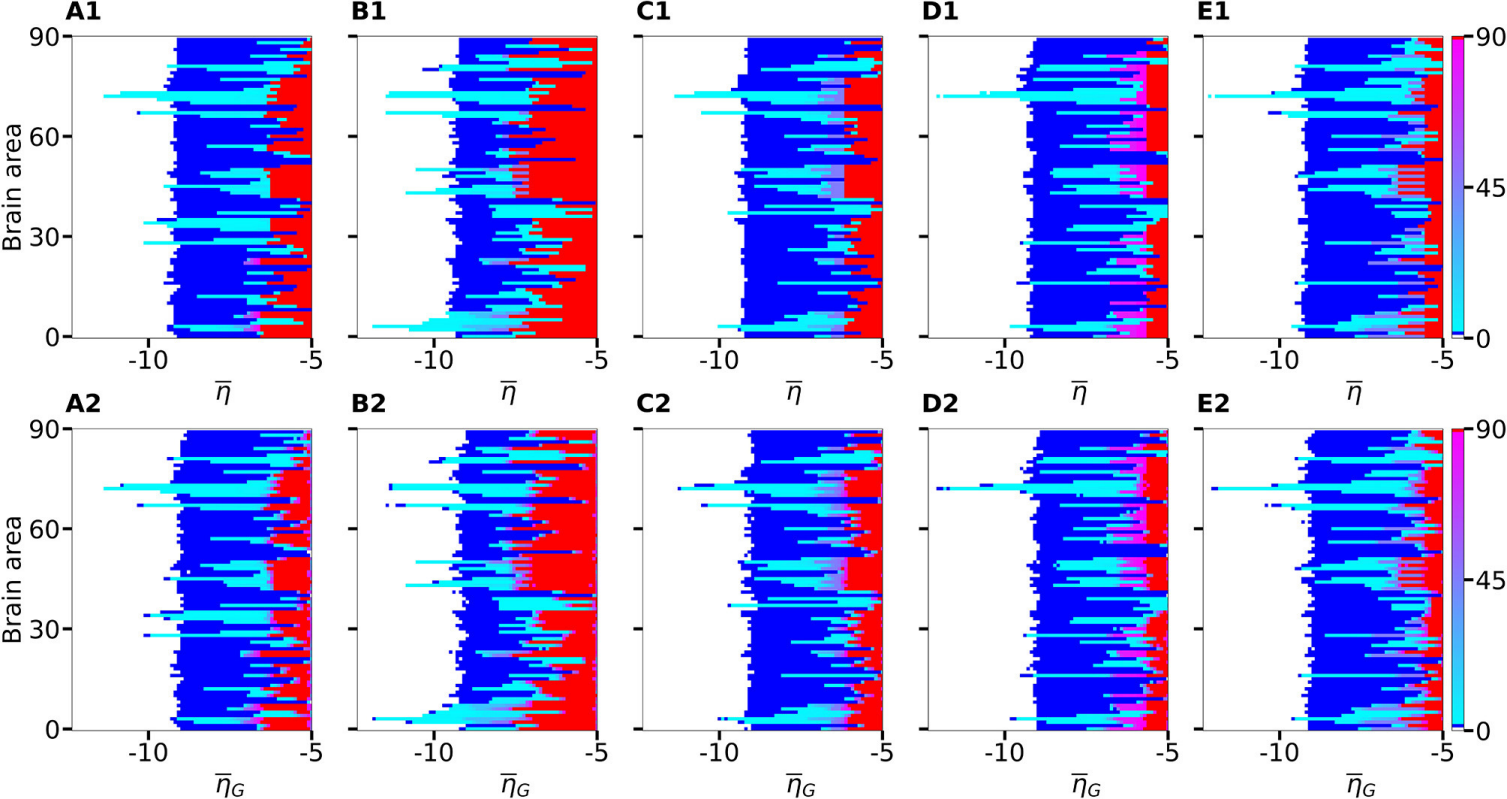
Number of recruited brain areas as a function of the excitability parameter $\bar{\eta }$ for five exemplary healthy subject connectomes **(A–E)**. Color coding is the following: blue corresponds to the asymptomatic threshold (one area in HA regime); red represents 90 areas in HA regime (generalized threshold); cyan to purple indicate intermediate recruitment values, white marks no recruitment. When performing a vertical cut, all nodes are characterized by the same $\bar{\eta }$ for panels **(A1–E1)**. On the contrary, in panels **(A2–E2)**, ${\bar{\eta }}_{\text{G}}$ represents the mean value of a Gaussian distribution with standard deviation 0.1. Therefore, when perturbing one brain area at a time, excitabilities are distributed and not uniform in the latter case; the results are averaged over 10 repetitions with different Gaussian excitability distributions. **(A–E)** correspond to subjects H1, H5, H12, H16, and H19. Parameters: *N*_pop_ = 90, Δ = 1, σ = 1, *I*_*S*_ = 10, *t*_*I*_ = 0.4 s.

In the following, we report a wide analysis of the impact of the perturbation site on the recruitment effect, for different excitability values. As before, we use a step current *I*_S_(*t*), with fixed amplitude *I*_*S*_ = 10 and duration *t*_*I*_ = 0.4 s, to excite a single population. In each run, the stimulating current targets a different brain area and the number of recruitments, i.e., the number of populations that pass from the LA state to the HA state is counted. The *N*_*pop*_ = 90 brain areas are targeted, one at a time, in 90 individual simulations. We repeat the procedure varying $\bar{\eta }$ in a range [−15, −4], with steps of $\Delta \bar{\eta }=0.1$. The results for five exemplary subjects are shown in Figures 3A1–E1).

If the perturbed area jumps back to the LA state when the stimulation is removed and no further recruitment takes place, then the total number of recruited areas is zero, the color is coded in white. If the perturbed area remains in the HA state without recruiting other areas, we are in presence of an asymptomatic seizure-like event (blue regions). For every further recruited brain area, the color code changes from cyan to purple. If all brain areas are recruited, we observe a generalized seizure-like event (coded as red). For $\bar{\eta }<-9$, most of the targeted brain areas goes back to the LA state, when the perturbation ends, while for $\bar{\eta }\approx -9$, we generally observe asymptomatic seizure-like events for all the subjects and most of the perturbation sites. For increasing $\bar{\eta }$ values, the probability for larger recruitment cascades increases, until the system exhibits generalized seizure-like events for $\bar{\eta }>-6$. However, some notable differences between brain areas and among the different subjects are observable. Brain area 72, for example, corresponding to the rh-CAU, exhibits asymptomatic seizure-like events at $\bar{\eta }>-11$ for most of the subjects, thus suggesting that the rh-CAU favors pathological behavior with respect to other brain areas. On the other hand, some brain areas are less likely to cause generalized seizure-like events, when stimulated, than others: brain area 40, for example, the rh-PHIP^1^, causes no generalized seizure-like events for any $\bar{\eta }>-5$. Note that, for very large $\bar{\eta }$ values, the system does not exhibit multistability anymore, but instead has only one stable state, namely the network HA state, corresponding to the high firing rate of all populations. Approximately, this happens for $\bar{\eta }\in \left[-5.7,-4.9\right]$, with small variations among the subjects.

The scenario remains unchanged when we take into account heterogeneous excitabilities ${\bar{\eta }}^{\left(k\right)}$, as shown in Figures 3A2–E2. In this case, ${\bar{\eta }}^{\left(k\right)}$ is drawn from a Gaussian distribution with mean ${\bar{\eta }}_{\text{G}}$, thus mimicking the variability among different brain areas present in a real brain. The populations are stimulated, as before, one at a time in individual simulation runs. However, this time the procedure is repeated for varying ${\bar{\eta }}_{\text{G}}\in \left[-15,-4\right]$, while keeping the standard deviation of the Gaussian distribution fixed at 0.1. Larger standard deviation (≥ 1) hinder the rich multistability of the network, by eliminating the bistability between LA and HA for individual populations, due to excessively small or large ${\bar{\eta }}^{\left(k\right)}$, thus impeding the analysis of the impact of the stimulation, as shown in the Supplementary Figure 1. In particular, for larger standard deviation, an increasing amount of nodes reaches the stable focus regime, thus being able to recruit other brain areas before the stimulation is applied, while nodes whose effective excitability turns out to be very small, are too far from the bistability region to be able to reach the HA regime. The results shown in Figure 3 are obtained averaging over 10 Gaussian distribution realizations of the $\bar{\eta }$ parameter; slightly more variability becomes apparent especially when considering the threshold in $\bar{\eta }$ to observe generalized seizures. Indeed, the excitability threshold to observe generalized seizures is the most drastically affected as the standard deviation increases, as shown in Supplementary Figure 1.

**Figure 4:**
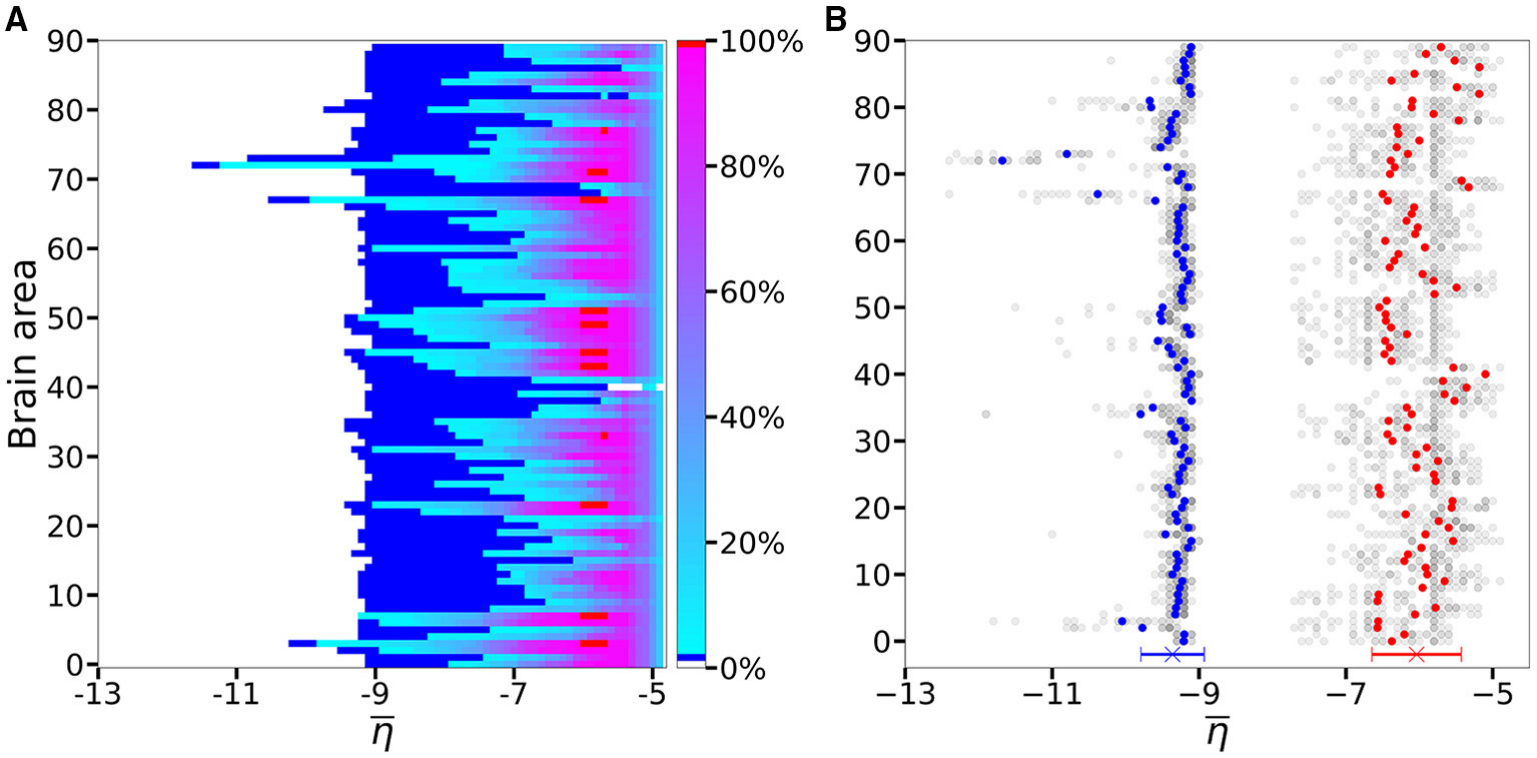
**(A)** Number of recruited brain areas as a function of the excitability parameter $\bar{\eta }$, as shown in Figures 3A1–E1, averaged across all subjects. **(B)** $\bar{\eta }$ threshold values for asymptomatic and generalized seizure-like events. Gray dots show the thresholds for each brain area and each subject. Blue and red dots show the average over ${\bar{\eta }}_{\text{asy}}^{\left(k\right)}$ and ${\bar{\eta }}_{\text{gen}}^{\left(k\right)}$ across all subjects. The blue and red cross at the bottom shows the average value and its standard deviation for both thresholds across all subjects and all areas. Parameters as in Figure 3.

An overview over all the investigated subjects is possible when looking at Figure 4A, where is reported the average, over-all subjects, of the data shown in Figures 3A1–E1 for five exemplary subjects only. The averaging operation smears out the transition contours, and while the region of generalized seizure-like events shrinks, it becomes wider in the region of accessibility of partial seizure-like events, where a small percentage of nodes (~ 20%) are recruited. In Figure 4B we report ${\bar{\eta }}_{\text{asy}}^{\left(k\right)}$ (${\bar{\eta }}_{\text{gen}}^{\left(k\right)}$), i.e., the smallest $\bar{\eta }$ value for which an asymptomatic (generalized) seizure-like event occurs when stimulating population *k*. Gray dots indicate the individual thresholds ${\bar{\eta }}_{\text{asy}}^{\left(k\right)}$ and ${\bar{\eta }}_{\text{gen}}^{\left(k\right)}$ for each of the 20 subjects and 90 brain areas; the averages over all subjects are denoted by blue and red circles, respectively, for each *k* ∈ [1, 90]. Averaging these thresholds over all subjects and brain areas yields an asymptotic threshold of ${\bar{\eta }}_{\text{asy}}=-9.36\pm 0.43$ and a generalized threshold of ${\bar{\eta }}_{\text{gen}}=-6.04\pm 0.38$. Brain areas 72, 73, 67, and 3 have lower thresholds for asymptomatic seizure-like events, areas 40, 86, and 82 have larger thresholds for generalized seizure-like events and do not fall within a standard deviation. The variability in the response among the different areas is more evident for ${\bar{\eta }}_{\text{gen}}^{\left(k\right)}$ values compared to the ${\bar{\eta }}_{\text{asy}}^{\left(k\right)}$ ones: the threshold values to obtain an asymptomatic seizure-like events are very similar among the areas and among the subjects, while the threshold values to obtain a generalized seizure-like event strongly depend on the stimulated area and on the subject.

#### 3.1.3. The Role Played by Brain Area Network Measures on Enhancing Recruitment

**Figure 5:**
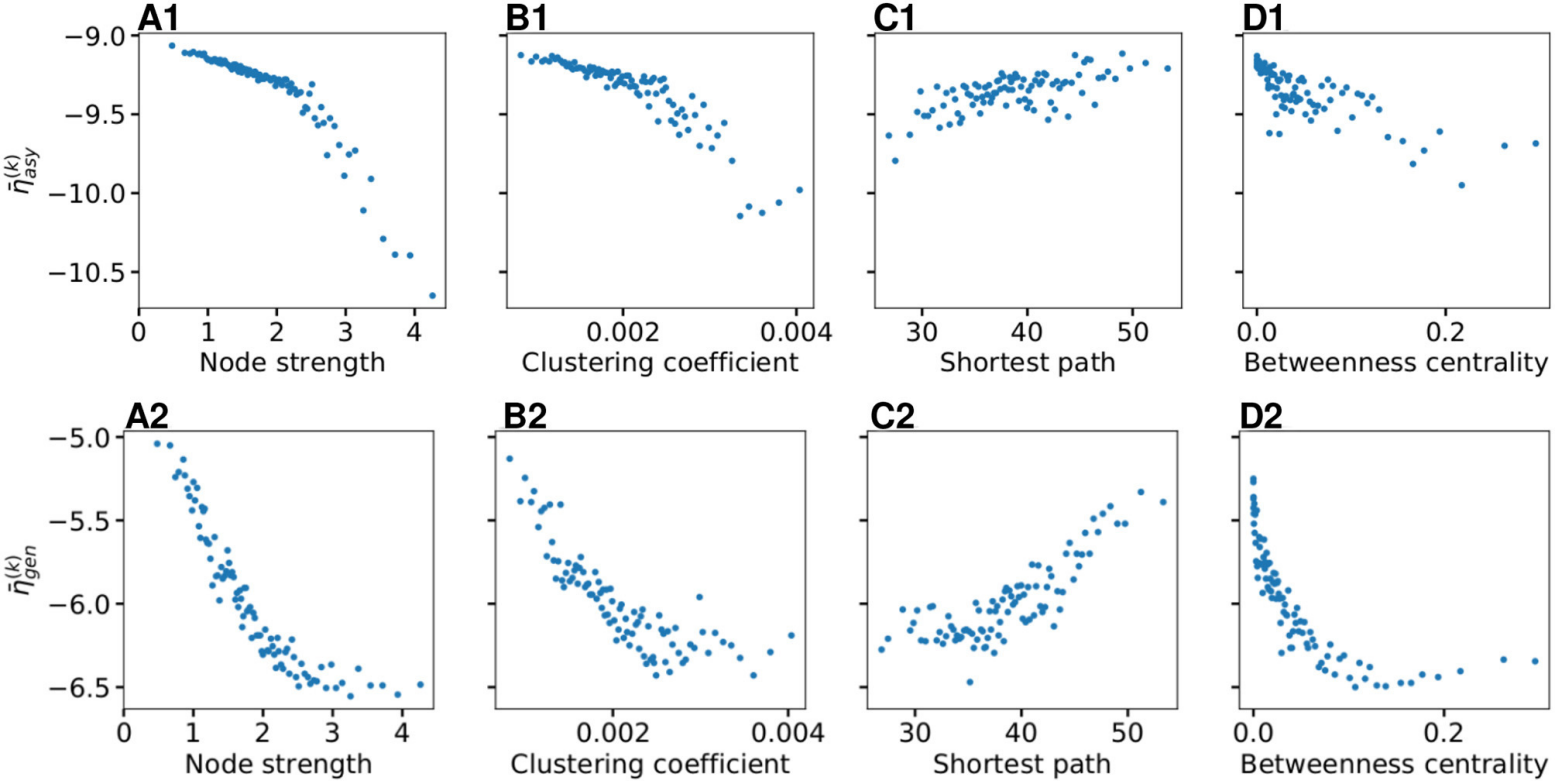
Thresholds ${\bar{\eta }}_{\text{asy}}^{\left(k\right)}$ for asymptomatic seizure-like events **(A1–D1)** and ${\bar{\eta }}_{\text{gen}}^{\left(k\right)}$ for generalized seizure-like events **(A2–D2)** as a function of node measures: **(A)** Node strength, **(B)** clustering coefficient, **(C)** average shortest path length, and **(D)** betweenness centrality. For each panel, the thresholds ${\bar{\eta }}_{\text{asy}}^{\left(k\right)},{\bar{\eta }}_{\text{gen}}^{\left(k\right)}$ are calculated for all *k* ∈ [1, 90] brain areas and averaged over all 20 subjects. Parameters as in Figure 3.

As shown in Figure 4B, ${\bar{\eta }}_{\text{asy}}^{\left(k\right)}$ does not vary significantly among the subjects and among the brain areas; it mainly occurs in the range ${\bar{\eta }}_{\text{asy}}^{\left(k\right)}\in \left[-10,-9\right]$, with just few nodes (*k* ∈[72, 73, 67, 3]) showing smaller values. Since each brain area is characterized by its network measure, the first hypothesis that we aim to test, is the role played in the identification of the threshold, by the different network measures. We will verify in the following that connection strength and shortest path length are determinants to identify the threshold ${\bar{\eta }}_{\text{gen}}^{\left(k\right)}$: Generalized seizure-like events are enhanced by nodes forming a clique that rapidly communicate through a dense subgraph. In particular, we investigate the dependency of ${\bar{\eta }}_{\text{asy}}^{\left(k\right)}$ on the node strength, clustering coefficient, shortest path length, and betweenness centrality of the corresponding brain area, as shown in Figure 5. A very strong correlation between asymptomatic threshold and node strength becomes apparent: Brain areas that are strongly connected, need smaller excitability to pass from the LA to the HA regime (Figure 5A1). The same holds for the clustering coefficient, even though the relationship is less sharp (Figure 5B1). Moreover, it is possible to observe a direct correlation between ${\bar{\eta }}_{\text{asy}}^{\left(k\right)}$ and shortest path length (i.e., shortest is the path and smallest is the threshold value), while betweenness is smaller for higher threshold values (Figures 5C1,D1, respectively).

When considering the threshold for generalized seizure-like events, we face a higher variability among different nodes (as shown in Figure 4B, ${\bar{\eta }}_{\text{gen}}^{\left(k\right)}$ varies mainly between −6.5 and −5.5). The dependency of ${\bar{\eta }}_{\text{gen}}^{\left(k\right)}$ on the node strength reveals a strong correlation: Areas with very small node strengths are characterized by large thresholds and are less likely to cause generalized seizure-like events. On the other hand, for large node strengths, ${\bar{\eta }}_{\text{gen}}^{\left(k\right)}$ saturates at a value ≈−6.5 (as shown in Figure 5A2). The clustering coefficient, shown in Figure 5B2), shows a similar relationship as the node strength, even though more scattered. This is not surprising since node strength and clustering coefficient are strongly correlated with each other (the Pearson correlation coefficient, in this case, is *r* = 0.75, as shown in Supplementary Figure 2), thus explaining the similarity between the analyses reported in Figures 5A,B. Moreover, regarding the integration measure, it turns out that the average shortest path length correlates positively with ${\bar{\eta }}_{\text{gen}}^{\left(k\right)}$ (as shown in Figure 5C2). Brain areas that are characterized, on average, by a short path to all the other areas are more likely to cause generalized seizure-like events. Finally, the betweenness centrality correlates negatively with ${\bar{\eta }}_{\text{gen}}^{\left(k\right)}$ (Figure 5D2). This means that brain areas that are crossed by many shortest path lengths (large betweenness centrality) are more likely to cause generalized seizure-like events. For increasing node strength, clustering coefficient, and betweenness centrality, we observe a saturation toward ${\bar{\eta }}_{\text{gen}}^{\left(k\right)}\approx -6.5$, that corresponds to the critical excitability value, during the up-sweep simulation, at which the system jumps to the HA network state (Figure 1A2).

**Figure 6:**
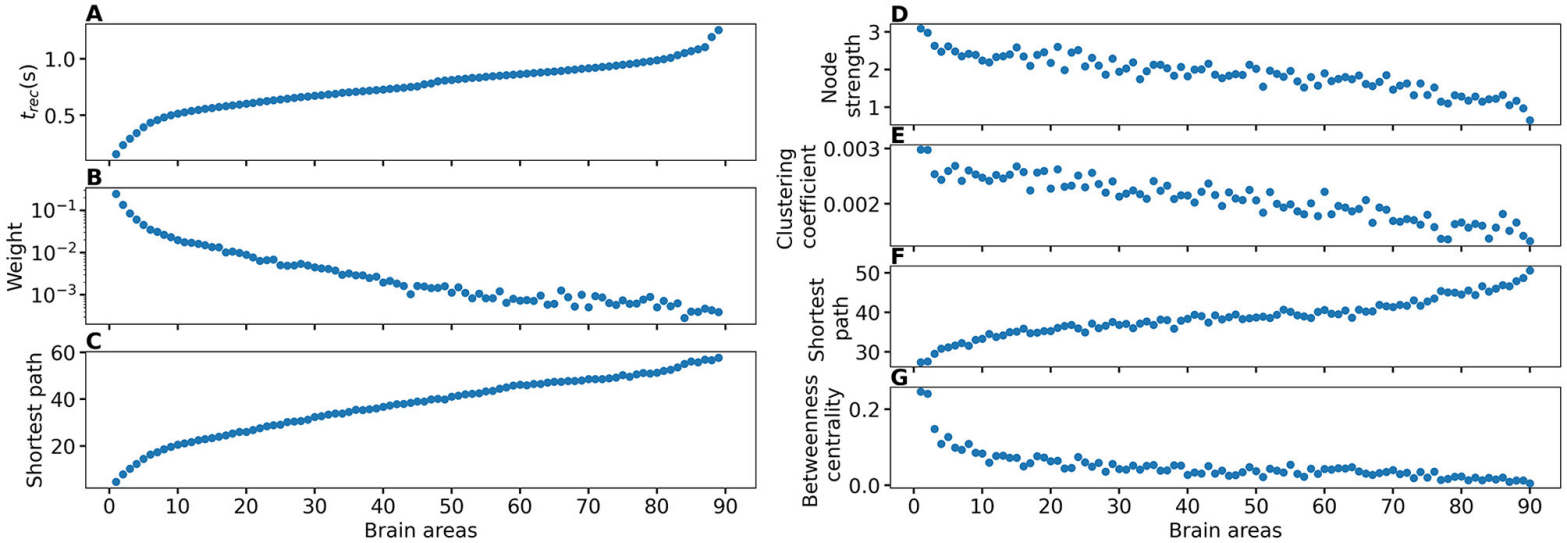
**(A)** Recruitment times reported in descending order: Brain area 1 is the brain area that is recruited first and brain area 90 is the last recruited brain area. **(B)** Connection weights between the recruited brain area and the EZ, ordered according to their recruitment time, thus following the indexing of **(A)**. **(C)** Shortest path between the recruited area and the EZ, ordered according to their recruitment time. **(D)** Connection weights between the recruited brain area and all the nodes except EZ, ordered according to their recruitment time. **(E)** Clustering coefficient between the recruited brain area and all the nodes except EZ, ordered according to their recruitment time. **(F)** Shortest path between the recruited area and all the other nodes except EZ, ordered according to their recruitment time. **(G)** Betweenness centrality between the recruited brain area and all the nodes except EZ, ordered according to their recruitment time. The excitability ${\bar{\eta }}^{\left(k\right)}$ is set to the subject-specific threshold ${\bar{\eta }}_{\text{gen}}^{\left(k\right)}$, according to Figure 3B for each subject separately. Data are averaged over all subjects and all the stimulated areas. Parameters: *N*_pop_ = 90, Δ = 1, σ = 1, *I*_*S*_ = 10, *t*_*I*_ = 0.4 s as in Figure 3.

To explore the causal mechanisms of brain dynamics and understand the sequential mechanism of node recruitment in more detail, we investigate the timing at which different brain areas are recruited. For this, the excitability parameter $\bar{\eta }$, common to all populations, is set to the threshold value ${\bar{\eta }}_{\text{gen}}^{\left(k\right)}$ of the perturbed brain area *k*, ensuring complete recruitment of all populations, when perturbing populations *k* ∈[1, 90]. The results shown in Figure 6 are obtained by averaging over *k* and the different subjects: in 90 individual simulations for each subject, a single brain area *k* = 1, …, 90 is stimulated with an external step current *I*_S_(*t*), characterized by an amplitude *I*_S_ = 10 and a duration *t*_I_ = 0.4 s. For each *k*, the recruitment time of all the other areas is registered. The stimulated brain area stands in for the EZ. The brain areas and corresponding node measures are sorted by the recruitment time in ascending order. The values for recruitment time (Figure 6A), the weight of a connection between a single area and the EZ (Figure 6B) and shortest path (Figure 6C) is finally obtained averaging over all the stimulated nodes and all the subjects (i.e., the average is performed over 1, 800 simulations across all 90 brain area perturbations times for all 20 subjects). The same averaging procedure has been employed to obtain the data shown in Figures 6D–G. However, in this case, the node measures are evaluated over all the connections of the recruited node minus the connection to the EZ. While ignoring the link to the exciting area (EZ), the overall network measure for connection weights (Figure 6D), clustering coefficient (Figure 6E), shortest path (Figure 6F), and betweenness centrality (Figure 6G) are reported.

On average, the first recruited brain area (labeled as 1) is connected to the EZ with a weight equal to 0.25 (1/4 of the maximum possible weight), and it is characterized by an average shortest path length to the EZ of <4.7. Moreover, the area is recruited within an average time of <156 ms (calculated after the onset of the external perturbation current). However, the first recruited area has, not only the strongest weight and the shortest path to the EZ but also has, in general, the largest node strength, largest clustering coefficient, shortest average path length, and largest betweenness centrality. The seizure-like event spreads rapidly along with the brain areas with strongest connection weights outgoing from the EZ; the stronger weights are associated with the shortest paths from the EZ. Overall, a region well connected is a region well recruited; this is related to the log-normal distribution of the weights (as shown in Supplementary Figure 3): few connections per node have a strong weight, thus allowing for fast recruitment. Note that the results for one exemplary subject and just one perturbed brain area per time (i.e., not averaged over all the brain areas and over all subjects) are comparable, even though the corresponding relationships are characterized by more variability (data not shown).

**Figure 7:**
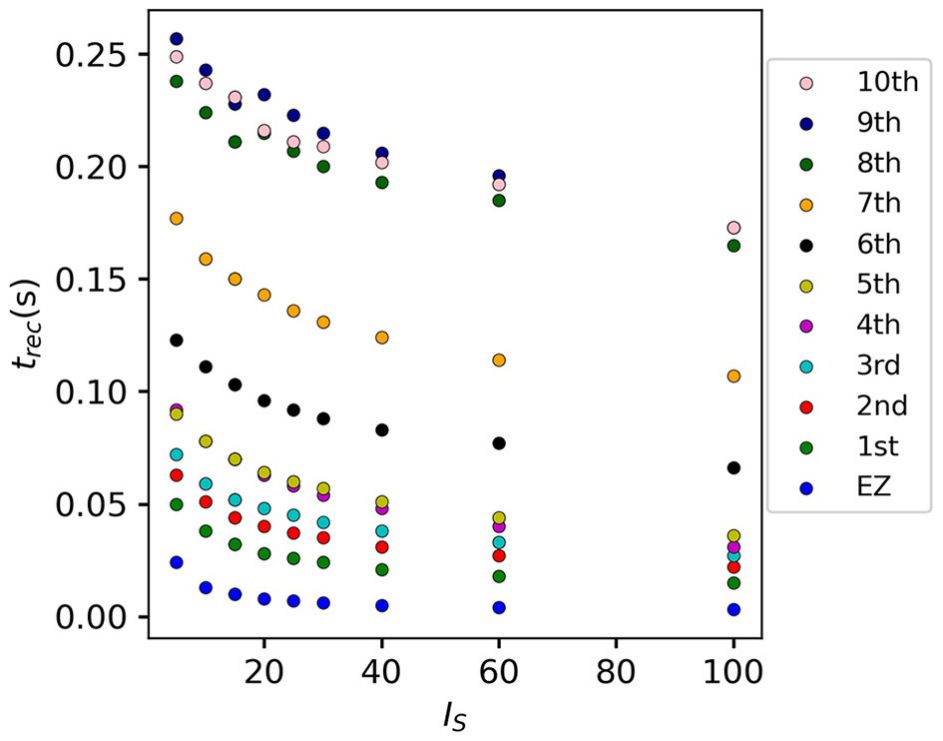
Recruitment times of the first 10 recruited areas as a function of the input current *I*_*S*_. The strength of the input current is varied between 0 and 100 on the *x*-axis. The order of the recruitment is color-coded for each current strength, and it changes slightly with different current strengths. Parameters: *N*_pop_ = 90, Δ = 1, σ = 1, *t*_*I*_ = 0.4 s, ${\bar{\eta }}^{\left(k\right)}=\bar{\eta }=-6$, stimulation site: brain area *k* = 45 of subject H1.

If we vary the amplitude *I*_S_ of the perturbation current, the recruitment time will vary accordingly, decreasing for increasing *I*_*S*_. In particular, in Figure 7 we show an exemplary case, obtained from the stimulation of one brain area (45), for a specific subject (results are similar for other trials). Irrespectively of the recruitment order, the time needed by the first 10 recruited brain areas to pass from the LA to the HA state decreases slightly for increasing amplitude. However, this decrease reaches saturation at a current value *I*_S_≈40 already. The order of recruitment varies little: we observe some exchanges between the 4-th and 5-th and between the 9-th and 10-th recruited areas. For example, for an amplitude *I*_*S*_ = 15, the 9th recruited area (dark blue circles) gets recruited earlier than the 10th area (pink dots), while, for very strong currents (*I*_*S*_ = 100), the 9th area gets recruited latest. On the other hand, we do not observe a significant change in the recruitment time and order if we increase the duration *t*_I_ of the stimulation (as shown in Supplementary Figure 4).

### 3.2. Epileptic Patients

#### 3.2.1. Phase and Bifurcation Diagrams

**Figure 8:**
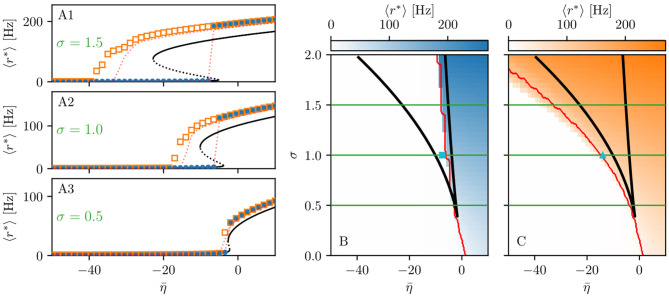
Phase and bifurcation diagrams for patient E6. **(A1–A3)** Equilibrium firing rates 〈r^*^〉 vs. $\bar{\eta }$ for the up sweep (blue dots) and down-sweep (orange squares). For each $\bar{\eta }\in \left[-50,10\right]$ in steps of $\Delta \bar{\eta }=1.5$, the system is initialized using the final state of the previous run and evolves for 2 s after which the average network firing rate in the equilibrium state is determined. Different panels correspond to different σ values: σ = 1.5 **(A1)**, σ = 1 **(A2)**, and σ = 0.5 **(A3)**. The solid (dashed) black line corresponds to the stable (unstable) equilibria in the single-node case. The dotted red line depicts the results for the healthy subject H1 reported in Figure 1. Maps of regimes as a function of σ and $\bar{\eta }$ showing the network average 〈r^*^〉 color-coded for up- **(B)** and down-sweep **(C)**, obtained by following the same procedure as in **(A1–A3)** for σ ∈[0, 2] in steps of Δσ = 0.05. The black line indicates the single-node map of regimes like in Montbrió et al. (2015). The red solid line indicates the boundaries of the map of regimes previously as shown in Figure 1 for the healthy subject H1. In **(B,C)** the cyan square and triangle mark $\bar{\eta }=-7.5,-14$, respectively. Parameter values: *N*_pop_ = 88, τ_m_ = 20 ms, Δ = 1, *J*_*kk*_ = 20, $J_{kl}=5{\tilde{J}}_{kl},\;\forall k\neq l$.

In this section, the structural connectivity matrices of epileptic patients are employed and an analysis, analogous to the one in section 3.1.1, is provided. We present the phase and bifurcation diagrams for the multipopulation neural mass model, employing the structural connectivity matrices of epileptic patients. As detailed before, the bifurcation diagrams shown in Figures 8A1–A3, for different σ values, are obtained by performing an adiabatic scan along ${\bar{\eta }}^{\left(k\right)}=\bar{\eta }$, following the up- and down-sweep protocols.

As for the healthy subjects, the transition is hysteretic with ${\bar{\eta }}_{\text{LA}}<{\bar{\eta }}_{\text{HA}}$. However, in this case, the width of the hysteretic transition is bigger, especially for larger σ values, as testified by the comparison with the dotted red curve, reported in Figures 8A1–A3, that represents the results shown in Figure 1. This increased width can be translated in terms of the extension of the multistability region in the phase diagram (as shown in Figures 8B,C), which turns out to be slightly larger than before. Also in this case, the results for a healthy subject are reported for a better comparison (continuous red curve in Figures 8B,C). The increase in size mainly occurs due to a shift of ${\bar{\eta }}_{\text{LA}}$, i.e., of the left boundary of the multistability regime. In this region, the transition from HA to LA, following the down-sweep, is more smooth and elongates toward smaller $\bar{\eta }$ values. This implies that, in this transition region, more single population HA states exist for epileptic patients than for healthy subjects. In other words, brain areas of epileptic subjects are more prone to recruitment^2^.

**Figure 9:**
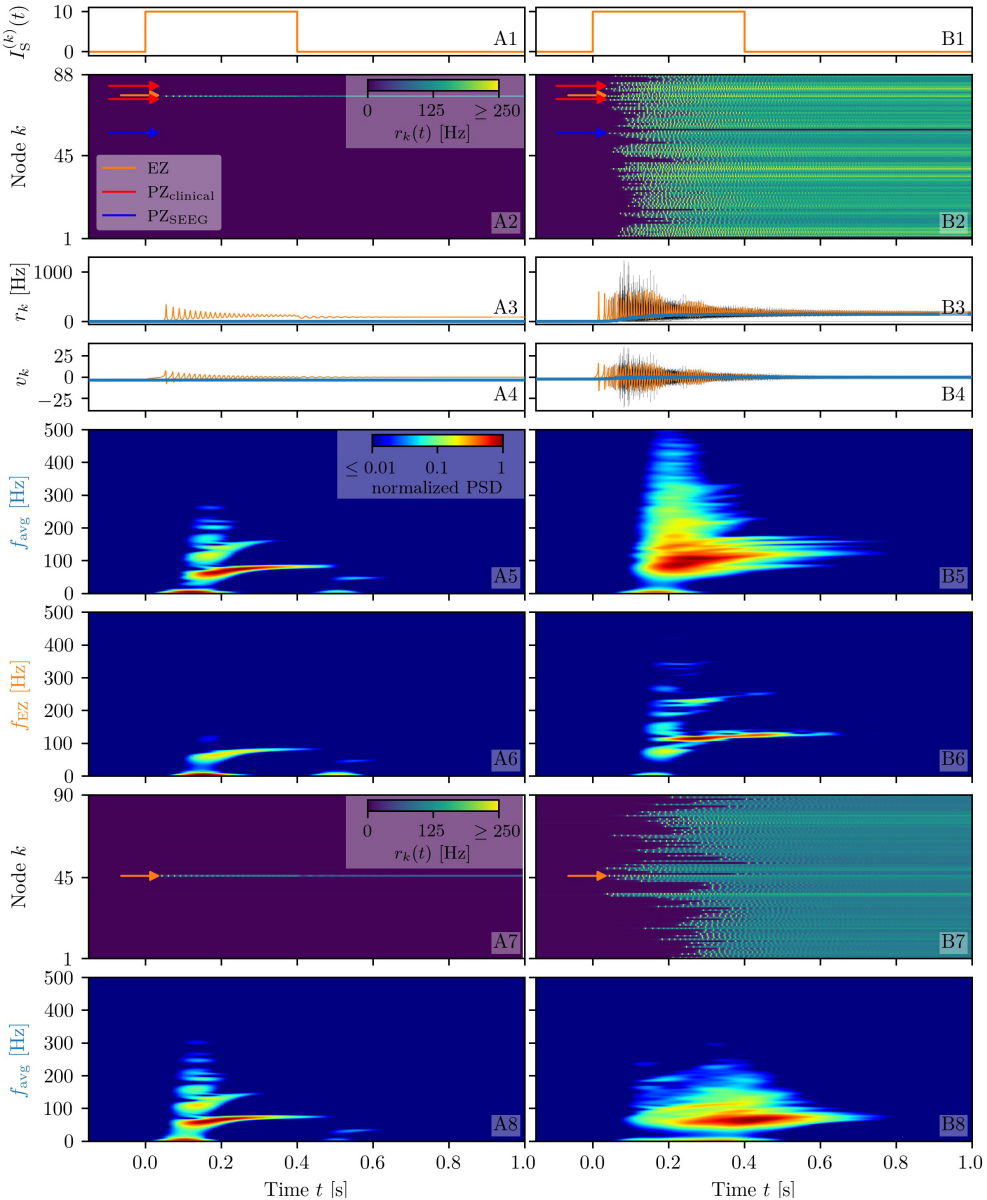
Spectrograms of mean membrane potentials for patient E6. **(A1, B1)** Stimulation current $I_{S}^{\left(k\right)}$, **(A3, B3)** population firing rates *r*_*k*_, and **(A4, B4)** mean membrane potentials *v*_*k*_ for the EZ (orange) and other populations (black). The blue curves show the network average firing rate and membrane potential. **(A2, B2)** Space-time plots of the population firing rates *r*_*k*_, color-coding the value of the firing rate of each node, as a function of time. **(A5, B5)** Spectrogram of the network average membrane potential and **(A6, B6)** of the *v*_*k*_ of the EZ. Column A shows an asymptomatic seizure-like event for $\bar{\eta }=-14$, column B shows a generalized seizure-like event for $\bar{\eta }=-7.5$. The EZ node 77 (rh-PrG) is stimulated. Parameter values: *N*_pop_ = 88, τ_m_ = 20 ms, Δ = 1, σ = 1.25, *J*_*kk*_ = 20, $J_{kl}=5{\tilde{J}}_{kl},\;\forall k\neq l$. For comparison are shown the space-time plots of the population firing rates *r*_*k*_ **(A7, B7)** and the spectrogram of the network average membrane potential **(A8, B8)** for healthy subject H2. In accordance with the above panels, column A shows an asymptomatic seizure-like event (for $\bar{\eta }=-9.20$), column B shows a generalized seizure-like event (for $\bar{\eta }=-5.3$). The EZ node 46 is stimulated. Parameter values: *N*_pop_ = 90, τ_m_ = 20 ms, Δ = 1, *J*_*kk*_ = 20, σ = 1, $J_{kl}=5{\tilde{J}}_{kl},\;\forall k\neq l$.

While the phase diagram is obtained in the absence of time-varying input, we investigate the response of the multipopulation system to transient stimulation in the following. As for the healthy subjects, a single population is excited by injecting a step current *I*_S_(*t*) of amplitude *I*_S_ = 10 and duration *t*_I_ = 0.4 s. Initially (*t* < 0), the system is in a multistable regime, starting in the low-activity network state. For small $\bar{\eta }$ values ($\bar{\eta }=-14$, identified by the triangle in Figure 8C), when a single node is stimulated, the system reacts analogously to the healthy subject case: during the stimulation, only one stable network state exists, i.e., a focus equilibrium with an LA-HA configuration for which only the stimulated node is in HA (Figure 9A2). This focus is approached *via* damped oscillations (0s < *t* < 0.4s). When the stimulation is removed, the network maintains the LA-HA configuration, but approaches the new location of the focus again *via* damped oscillations (Figures 9A3,A4). As a result, the stimulated node has large firing activity, while the remaining network is in a LA regime. For higher excitability values ($\bar{\eta }=-7.5$, identified by the square in Figure 8B), the perturbation of a single node gives rise to a cascade of recruitments, where other brain areas, initially not perturbed, reach the HA regime by showing damped oscillations (Figures 9B2–B4). With respect to the recruitment features shown in Figure 2, we observe in this study a faster emergence of the generalized seizure-like event: once a brain area is stimulated, the others react, in-substantial number, quite immediately.

Looking at the spectrograms, the transition of the stimulated population to the HA regime is characterized by a transient activity at low frequency (< 20 Hz) and a sustained activity in the γ band (50–180 Hz), observable throughout of the stimulus, as shown in Figure 9A6, where the spectrogram for the single stimulated population is reported. Regarding the spectrogram of the mean membrane potentials averaged over the network populations (Figure 9A5), it turns out that the low-frequency activity in the δ, θ bands is present, while the activity at high frequency simply reflects the activity of the stimulated area. Activity in the δ band, together with multiple types of α-like rhythms have been recently found in a network of two Jansen-Rit neural mass models, representing two cortical regions, as a result of input changes in the other region (Ahmadizadeh et al., 2018), thus confirming that the range of possible activity varies with changes in the external inputs and interconnectivity gains.

In the case of large recruitment events, at larger excitability values, it is possible to observe γ activity at higher frequencies (as shown in Figures 9B5,B6), which is enhanced with respect to the situation where an asymptomatic seizure-like event is present. Moreover, comparing the spectrograms in Figure 9 and those reported in Figure 2, we see that the activity takes place at higher frequency ranges when considering structural connectivity matrices of epileptic patients and the activity is mainly concentrated in the EZ. A further comparison is possible, looking at Figures 9A8–B8, where the spectrograms for the healthy subject H2 are reported. With respect to the case shown in Figure 2, the excitability parameter has been increased to observe a faster *domino-like* effect, on the same temporal scale as for the epileptic patients. While high-frequency oscillations (>200 Hz) are observable for the epileptic patient case, they are not detectable in Figure 9B8 for the healthy subject case. The last statement may be qualified, however, by recent studies proposing high-frequency oscillations (80–500 Hz) recorded not only at seizure onset but also between seizures (the interictal period), as a putative new marker of the epileptogenic focus (Jacobs et al., 2012). More specifically fast cortical ripples superimposed to interictal epileptiform discharges were correlated with the seizure onset zone and primary propagation area in neocortical epilepsy (Khadjevand et al., 2017). Neocortical ripples were also found to be more specifically confined to the seizure onset and propagation regions, and thus a better marker compared to interictal epileptiform discharges alone (Wang et al., 2013). High-frequency oscillations, as obtained *via* numerical experiments and shown in Figures 9B5,B6, are much more frequent in the seizure-like onset zone than outside, where they are often totally absent. The rather empty spectrograms of mean membrane potentials for patient E6 are a result of rather rapid recruitment of a majority of nodes, thus giving rise to a strong signal change, immediately upon recruitment, which suppresses the rest of the signal in the spectrogram. At the same time, the damped oscillations are all compressed within a narrow time window, and not very elongated in time, as it happens for healthy subjects (as shown in Figure 2). In other words, if the generalized seizure-like event is rapid, all the signals overlap, and this is especially clear looking at the strong low-frequency bands. A fast generalized seizure-like event, in absence of high-frequency oscillations outside the EZ, can be obtained for healthy subjects only increasing the excitability parameter: for higher $\bar{\eta }$ values, the recruitment is more sudden, as shown in Figure 9B8). A difference between the signals obtained by numerically simulating the multipopulation exact neural mass model and the high-frequency oscillations observed in human intracranial EEG studies can be found in the different oscillation amplitudes: high-frequency oscillations recorded during pre-surgical evaluation in patients, both at the seizure onset and during the interictal period, are characterized by a low amplitude (Allen et al., 1992; Traub et al., 2001; Worrell et al., 2004; Zijlmans et al., 2012), while this is not the case in this study. We can conjecture that higher amplitudes are related to the nature of the coupling, which we have chosen globally coupled and fully excitatory.

#### 3.2.2. Temporal Recruitment of Clinically and SEEG Predicted PZs

**Figure 10:**
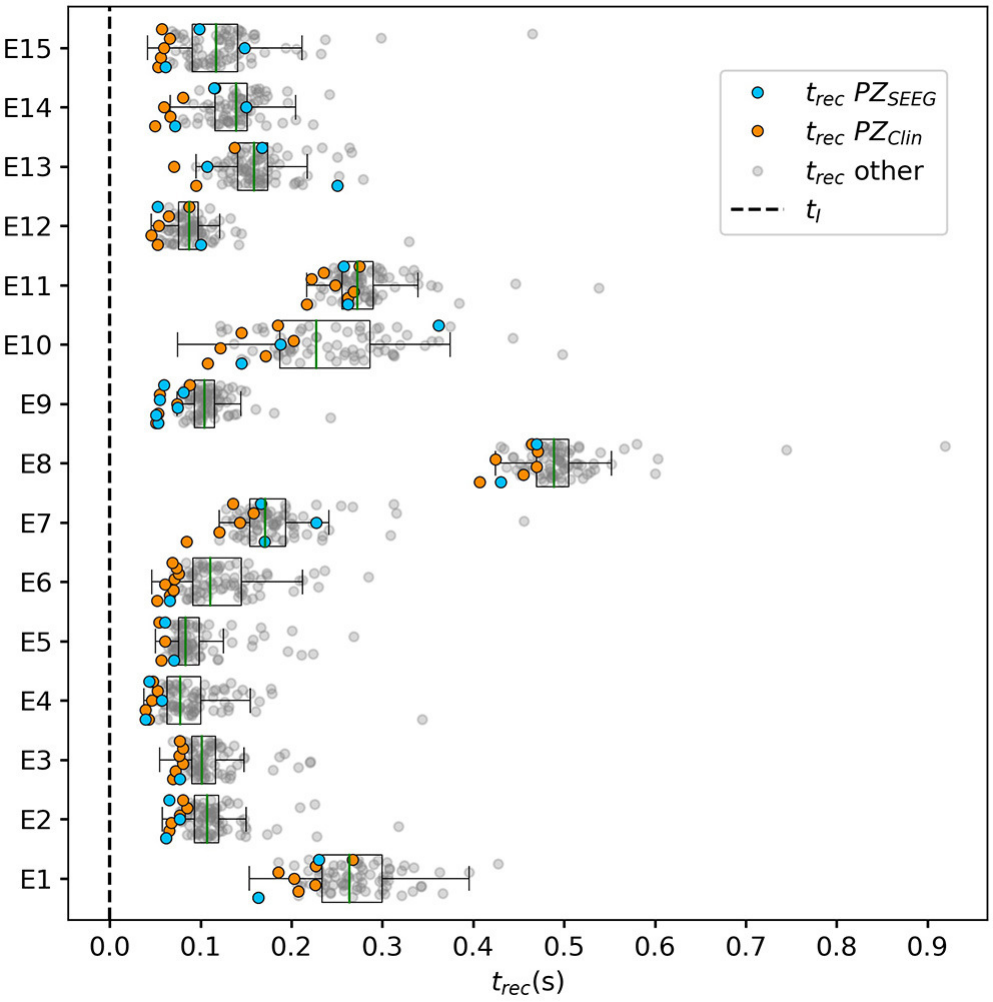
Recruitment times of all brain areas for the cohort of epileptic patients: The recruitment time, reported on the *x*-axis, identifies the time needed by a brain area to jump to the HA regime after the application of the perturbation current. The boxplots consist of the recruitment times of all brain areas for each patient. Patients are identified according to their numbers on the *y*-axis. The median is represented as a green vertical line while the boxes contain the second and third quartile of the distribution. The whiskers are chosen with a maximum length of 1.5 × IQR (interquantile range) and show the most extreme observed values that are within 1.5 × IQR from the upper or lower quartiles. The gray dots represent the recruitment times for each brain area. The orange dots show the recruitment of a brain area clinically predicted to be part of the PZ_Clin_. The blue dots represent the recruitment of a brain area that is part of the PZ according to the SEEG measurements PZ_SEEG_. Parameters: *N*_pop_ = 88, Δ = 1, σ = 1.25, *I*_*S*_ = 10, *t*_*I*_ = 0.4 s, ${\bar{\eta }}^{\left(k\right)}=\bar{\eta }=-7.5$ [except for patients E1 ($\bar{\eta }=-6$) and E11 ($\bar{\eta }=-6.5$)].

In the following, we test the clinical predictions for epileptic patients, by choosing the EZs, identified by clinical doctors *via* presurgical invasive evaluation, as perturbation sites. We investigate the recruitment times of different brain areas following such a perturbation and compare the order of recruitment with the experimental data given for each subject. A general overview of the recruitment times of all brain areas, for all patients, is shown in Figure 10. As perturbation sites, the clinical EZs are used for all patients. For patients with several nodes detected in the EZ, all areas were stimulated simultaneously. The perturbation step current (*I*_S_ = 10, *t*_I_ = 0.4 s) is applied, to each perturbation site, in correspondence with the dashed vertical black line. The parameters are identical for almost all patients and are chosen such that at least 90% of the brain areas are recruited while still allowing multistability among various LA-HA configurations, including the network LA state. For each patient (identified *via* his/her number on the y-axis), the recruitment time of each brain area is reported: the gray dots represent the time values for each brain area. Superimposed on the gray dots are orange and blue dots that identify the brain areas belonging to the PZ, according to the non-invasive (PZ_Clin_) or invasive (PZ_SEEG_) presurgical evaluation, respectively. The recruitment time-averaged over all brain areas is identified, for each patient, by a green vertical line, while the boxes contain the second and third quartile of the distribution, and the whiskers have 1.5 the length of the interquartile range (IQR) from the upper or lower quartiles. A one-sided Mann Whitney *U*-test has been performed to estimate the statistical significance of PZ_SEEG_ and PZ_clin_ recruitment times, as shown in Supplementary Figure 5. Remarkably, the propagation zones PZ_Clin_ and PZ_SEEG_ turn out to be among the first recruited brain areas for all patients in the numerical experiments. However, the temporal dynamics vary for all patients, with E8 and E1 having late recruitments. Looking at the set of the first 10 recruited brain areas for each patient (reported in detail in Supplementary Tables 5–7), we notice that most of the areas, identified by clinicians as belonging to the PZ, are actually within this set: for patients E4, E5, E6, E9, and E15, all the areas belonging to PZ_Clin_ are among the first 10 recruited areas, while the same holds for patients E2, E3, and E6 if we consider the areas identified by the SEEG analysis as belonging to the PZ_SEEG_. In general, a large number of the first 10 recruited areas, as revealed by the simulations, coincides with the areas that are supposed to be crucial in the seizure spreading according to the medical doctors (e.g., for patients E2, E3, E10, E12, E13, and E14). Moreover, the predictability of the model is higher if we compare the results with the predictions PZ_Clin_, while brain areas belonging to the PZs, are in general recruited before the median recruitment time. However, the model predictions are not good for the following cases: for patients E1, E8, E11, and E14, the areas belonging to the PZ_SEEG_ are only occasionally identified (half or less than half of the times), while for patients E1, E8, and E11, other nodes are generally recruited before those belonging to the PZ_Clin_, that are identified with a percentage <50%. In all the former bad cases, the EZ has not been correctly identified, as results from the relative surgical outcomes (as shown in Supplementary Table 3). Therefore, the incorrect identification of the origin of seizure-like events may lead to a misleading identification of the PZ: in other words, a different potential EZ will lead to a different recruitment order, possibly closer to the experimental data.

**Figure 11:**
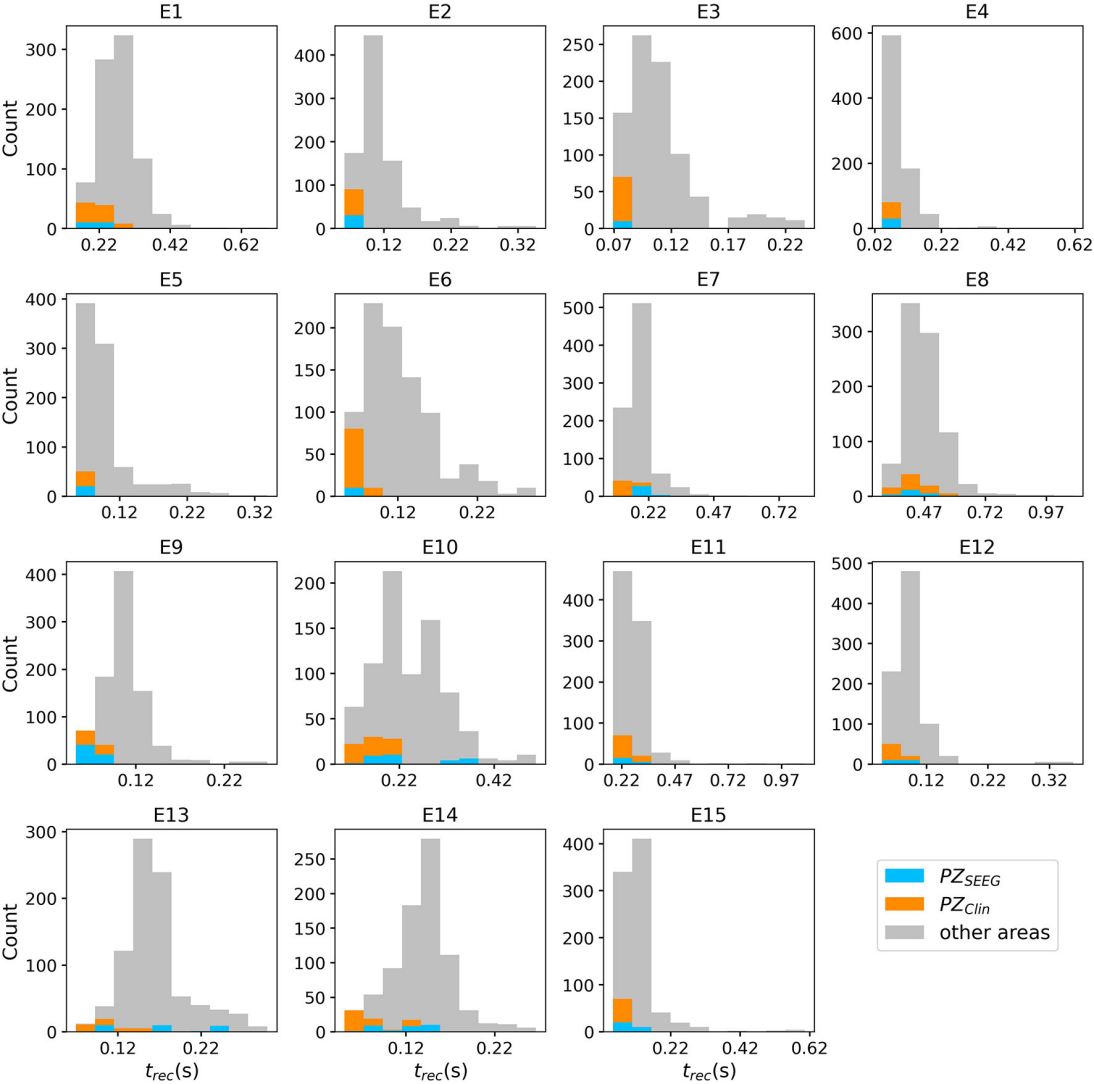
Histograms of recruitment times for all patients with epileptic. For each patient (identified by his/her number), the recruitment times of all the brain areas are collected, once the EZ is stimulated. If several areas were identified in the EZ, they are all stimulated simultaneously. The EZ is chosen according to the presurgical evaluation (as shown in Supplementary Table 4) and vary from one patient to the other. Parameters as in Figure 10 except for ${\bar{\eta }}^{\left(k\right)}=-7.5\pm 0.1$ (for E1 ${\bar{\eta }}^{\left(k\right)}=-6\pm 0.1$, for E11 ${\bar{\eta }}^{\left(k\right)}=-6.5\pm 0.1$). Results are averaged over 10 repetitions of different random Gaussian distributions.

To evaluate the dependence of the shown results on the chosen parameters, with the idea in mind of going toward a more biologically realistic framework, we have repeated the previous numerical experiment by employing a random Gaussian distribution of the excitability parameter ${\bar{\eta }}^{\left(k\right)}$ (as shown in Figure 11). The distribution is centered at ${\bar{\eta }}_{\text{G}}=-7.5$ with standard deviation 0.1 for all patients except E1 and E11. For the latter patients, we shifted the center toward larger values, to get a sufficient number of recruitments when the EZ is stimulated. In all cases, the results are averaged over 10 different random realizations of the distribution. More details on the impact of different realizations of ${\bar{\eta }}^{\left(k\right)}$ are given, for one exemplary patient, in Supplementary Figure 6. For sufficiently larger standard deviation than the one employed (≥1), a too large fraction of the populations would not be able to exhibit bistability between LA and HA, highlighting the system sensitivity to finite parameter changes. However, for the chosen distribution, the results are comparable with the ones obtained with identical ${\bar{\eta }}^{\left(k\right)}=\bar{\eta }$, shown in Figure 10. For patients E2, E3, E4, E5, E6, and E9 the predicted PZ are always the first ones to be recruited. Moreover, most of the areas are usually recruited in the first half of the recruitment process, rapidly increasing in number, once the areas in the PZ have been recruited (thus giving rise to a peak in the histogram). As a general remark, in view of the distributed nature of the excitabilities, recruitments at later times, with respect to the former case with homogeneous ${\bar{\eta }}^{\left(k\right)}=\bar{\eta }$, may now take place.

For patients with many nodes in the EZ, the recruitment process may result to be more complex, as it happens for patients E14 and E10, for which the histograms are less narrow, but instead widely distributed. However, this cannot be taken as a general rule, since comparable histograms are obtained for patients E13 (one node in the EZ) and E8 (two nodes in the EZ), while for E15 and E12 (with both four nodes in the EZ) the histograms result to be very narrow, thus implying a fast recruitment process of most of the brain areas. The differences among the histograms can be partially justified by the fact that patients have specific connectomes with individual characteristics and by the analysis that we have proposed by choosing similar $\bar{\eta }$ values for all the patients. In this way, we have preferred to have a general look at the multiple self-emergent dynamics in a group of patients, instead of fine-tuning the excitability parameter to obtain similar collective behaviors. What we observe in this study is strongly related to what we have presented in Figure 9: The recruitment speed depends on the excitability parameter and the individual network structure. Faster recruitment events may be obtained for different subjects by increasing the excitability value. In the following section, we try to understand, based on network topological measures, the origin of the discrepancies among the clinical prediction of PZs and the first recruited areas predicted by the presented model.

#### 3.2.3. Relationship Between DTI Network Structure and Temporal Seizure Recruitment

**Figure 12:**
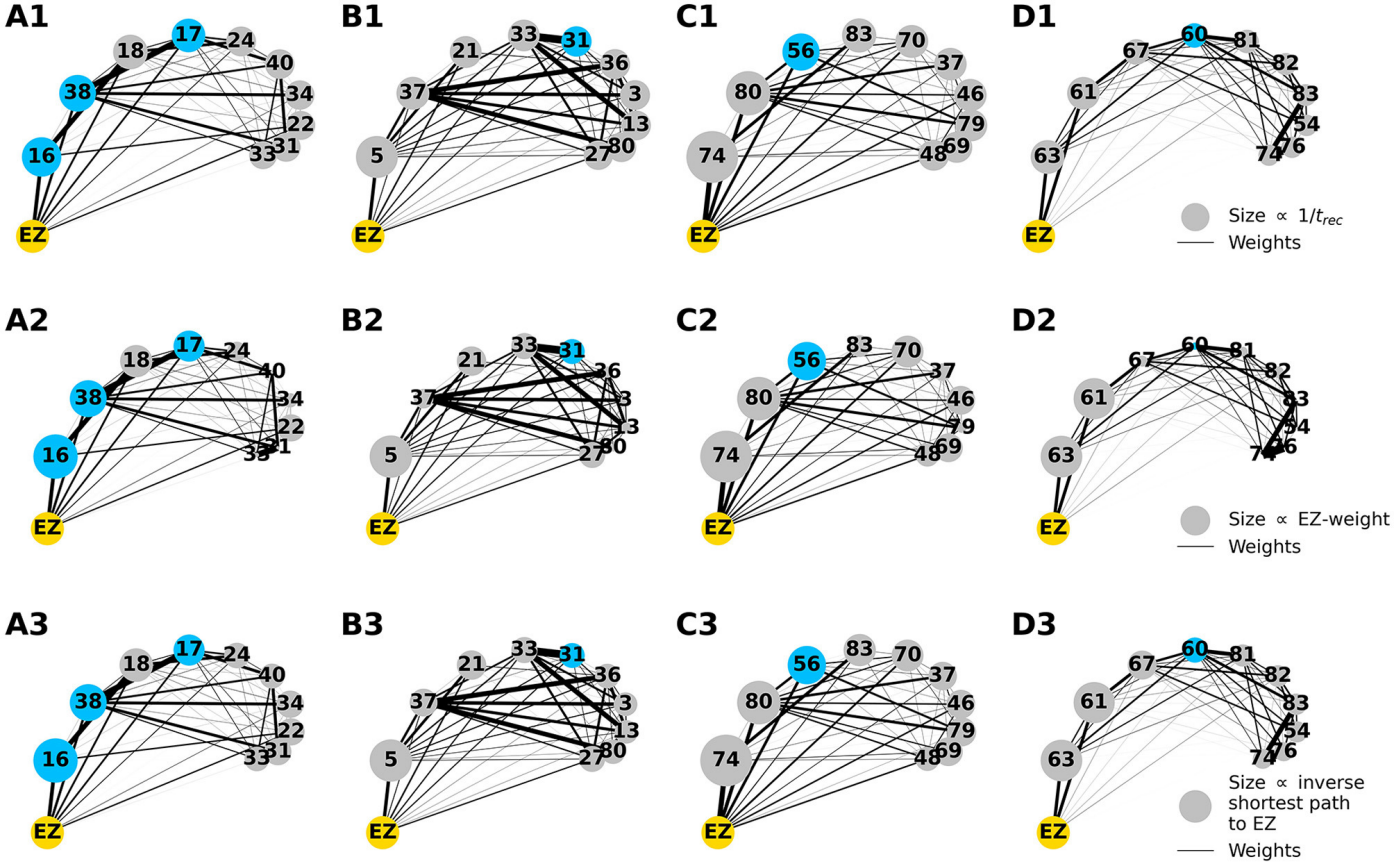
Graph plot of the first 10 recruited areas, ordered clockwise according to their recruitment times, as found *via* numerical experiments. Node circle size corresponds to the inverse recruitment time **(A1–D1)**, to the connection strength to the EZ **(A2–D2)**, and the inverse shortest path length to the EZ **(A3–D3)**. The size of the yellow EZ remains fixed. Blue dots distinguish a recruited area to belong to the PZ_SEEG_, i.e., the PZ identified according to the presurgical invasive evaluation. Results are obtained for patients E2 **(A1–A3)**, E3 **(B1–B3)**, E6 **(C1–C3)**, and E13 **(D1–D3)**. Parameters as in Figure 10.

To understand the mechanism underlying the recruitment events, we evaluate the relationship between the network structure, in terms of topological measures, and the recruitment times of the first 10 recruited brain areas, as obtained through numerical experiments. For simplicity, we consider in this study patients with only one brain area in the EZ and we report, in Figure 12, the potential EZ (yellow circle) and the first 10 recruited areas in a graph representation. The results relative to all the other patients are reported in the Supplementary Figures 7–9. The first recruited areas are ordered according to their recruitment times in clockwise order. Moreover, we indicate in blue the areas belonging to the PZ, as identified according to the presurgical invasive evaluation (PZ_SEEG_). Black lines identify the weighted connections between all areas and their thickness is proportional to their weight. The sizes of the circles representing each brain area are proportional to their inverse recruitment time (Figures 12A1–D1), to their weight connecting each area to the EZ (Figures 12A2–D2), and to their inverse shortest path length between each node and the EZ (Figures 12A3–D3), while the size of the yellow EZ circle remains fixed.

Since in (Figures 12A1–D1) the node size is proportional to the inverse recruitment time, large circles indicate early recruitment while small circles indicate late recruitments; hence, the circles become smaller clockwise. In panels (Figures 12A2–D2) the node size is proportional to the weight connecting each area to the EZ and it turns out that, for all patients, the first recruited area has the strongest connecting weight. However, after a few recruitments, this does not hold anymore. There are many examples in which areas with a strong weight to the EZ (as shown in e.g., area 46 or 48 for patient E6) are recruited much later than areas with very small weights (e.g., area 83 for FB). The seizure-like event propagates as a chain reaction and, therefore, the strongest connecting weight to the EZ is only decisive for the very first recruited area. Later, strong connections to other early recruited areas play a decisive role, as it is the case for area 83 in E6 which has a weak connection weight to the EZ. However, through its strong connection to area 74, its weighted shortest path length to the EZ is quite short, thus meaning that the weighted shortest path length to the EZ cannot be underestimated to find the recruitment order. Indeed, in (Figures 12A3–D3) one can see the good predictability of the shortest path: the node size, proportional to the inverse shortest path length to EZ, decreases in general with later recruitment. This is expected, given the fact that the average shortest path to the EZ considers all connections in the network, not just the connections subgraph outgoing the EZ. An example of the high predictability of the shortest path is given by node 38 in patient E2, which has a shorter path length to the EZ than node 18. Node 38 is recruited before node 18 irrespectively of its strong connection to node 16 and a connection strength to the EZ comparable with the one of node 38. However, it is worth noticing that, in general, the nodes that are recruited before the areas belonging to the PZ, show either stronger connecting weights, or shortest path length to EZ.

For later recruitments, the prediction becomes even more difficult because one needs to account for the temporal order of the seizing brain areas. As shown before, the area which is first recruited is the one with the strongest connection to the EZ. However, depending on the strength of the connection, the recruitment time changes and it increases for decreasing strength. In the case of patient E2, the recruitment of the second area is determined, more by the strength of the connections to the EZ (i.e., area 20) than by the connection to area 16, while for the recruitments of the third and fourth areas, the strong connections of node 18 to 16 and of node 17–38, i.e., the first and second recruited nodes, are fundamental. On the other hand, when the first recruited areas have strong connections to the EZ, for example area 74 in patient E6, the successive recruitments are strongly influenced by the first recruited area, whose outgoing graph reveals areas that are recruited with high probability. Thus, the connection to area 74 turns out to be, for the second, third, and fourth recruitment almost as important as the connection to the EZ (i.e., area 76). Finally, if we compare two late recruited areas that are characterized by the same shortest path length to the EZ but with a path to the EZ that crosses very different nodes, we observe that the area with the path going through earlier recruited nodes is recruited earlier. The longer the seizure-like event propagates, the less important the shortest path length to the EZ becomes and the more important the path lengths to other recruited nodes become. This underlines the difficulty of predicting the seizure propagation in complex networks, however, it is possible to summarize some findings that hold for almost all patients (including those shown in the Supplementary Figures 7–9): The first recruited node is, in general, the one with the strongest connection to the EZ and the shortest path; strong connections to early recruited areas are fundamental to determine the recruitment order; nodes belonging to the PZ_SEEG_, that are not identified by the simulations as first recruited nodes, show intermediate values of connection strength and shortest path, while the nodes that are recruited before are either more strongly connected the EZ or to the previously recruited nodes.

**Figure 13:**
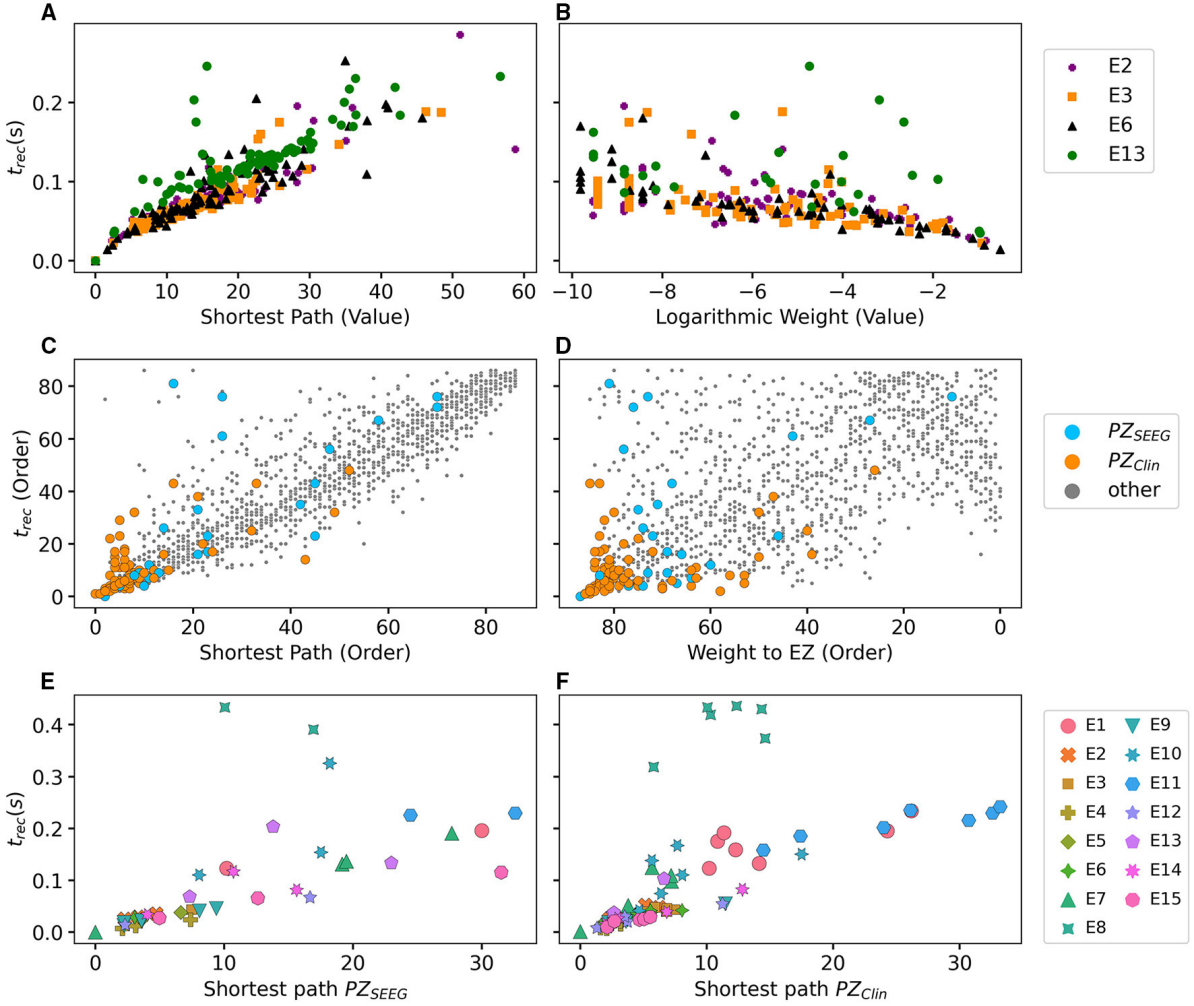
Relationship between network measure and recruitment time, as found *via* numerical experiments. **(A)** Shortest path to EZ; **(B)** Logarithmic value of the weight to the EZ for the four patients with a single-node EZ. In **(A)** all four EZs are shown at (0, 0), while in **(B)** the EZs are omitted. The recruitment time is calculated in seconds after the perturbation current has started. In **(C,D)** the recruitment time values are plotted according to their order, as a function of the shortest path to EZ **(C)** and weight to EZ **(D)** for all 15 patients. In **(D)** the *x*-axis was inverted for better comparison. **(E)** Recruitment times *t*_*rec*_ of the areas belonging to PZ_SEEG_ and **(F)** PZ_Clin_ as a function of the shortest path length to EZ, for all 15 patients. For patients with several nodes detected in the EZ, all areas were stimulated simultaneously. Parameters as in Figure 10.

To confirm the importance of the shortest path length and the strength of the connections outgoing the EZ in determining recruitment events, we report in Figure 13 the recruitment time values as a function of the shortest path and the connection weights for the patients with a single node as potential EZ (Figures 13A,B) and for all 15 epileptic patients (Figures 13C,D). While in Figure 13B, the recruitment time is plotted over the logarithm of the weight, in Figures 13C,D the values of the recruitment time, plotted as a function of the shortest path (connection weight), are ordered according to their recruitment order. In particular, the order for recruitment, shortest path, and weight to EZ is ascending from small values to large values. This means that, in Figure 13D, the areas with the strongest weights (87th, 86th, etc.) correspond to the areas that are recruited earliest (1st, 2nd, etc.). The ordering has been preferred to the specific values of the shortest path and connection weight when reporting data for all 15 patients, to obtain a better visualization. For patients E2, E3, E13, and E6, the recruitment time grows almost linearly with the shortest path, while it decreases for increasing weights. This analysis is confirmed in Supplementary Figure 10, where a regression fit is performed over the data shown in Figure 13A, thus underlying the approximately linear relationship between the shortest path length and the recruitment time for larger *t*_*rec*_. The relationship is not anymore so evident when we consider different cases of potential EZs, that is composed of more that one area. However, in this case, it is still possible to affirm than the earliest recruitments are associated with the shortest path lengths and the strongest weights, while the nodes corresponding to PZ_SEEG_ or PZ_Clin_ that, according to the simulations, were recruited late, have very long shortest path lengths to the EZs or very small weights.

In general, the recruitment mechanism is not completely defined by the shortest path length and the connection weight, therefore, it is not possible to match the pre-surgical predictions in terms of PZ_SEEG_ and PZ_Clin_ if we try to identify the nodes belonging to the PZ by calculating the first recruited nodes according to their shortest paths length or their connection weights. In particular, it turns out that the PZ_SEEG_ areas are well predicted by the investigated model if the shortest path length between the predicted PZ and the EZ is short, as shown in Figure 13E. However, for patients E8 and E10, the recruitments of the nodes belonging to PZ_SEEG_ happen much later when compared to brain areas of other patients with a similar shortest path length. Equivalently in Figure 13F it is possible to observe that, for short values of the shortest path length (<5), there is a linear correspondence between short recruitment times and PZ_Clin_ areas that are characterized by small values of the shortest path. However, the areas belonging to PZ_Clin_ are still not identifiable, in terms of topological measures, for patient E8.

**Figure 14:**
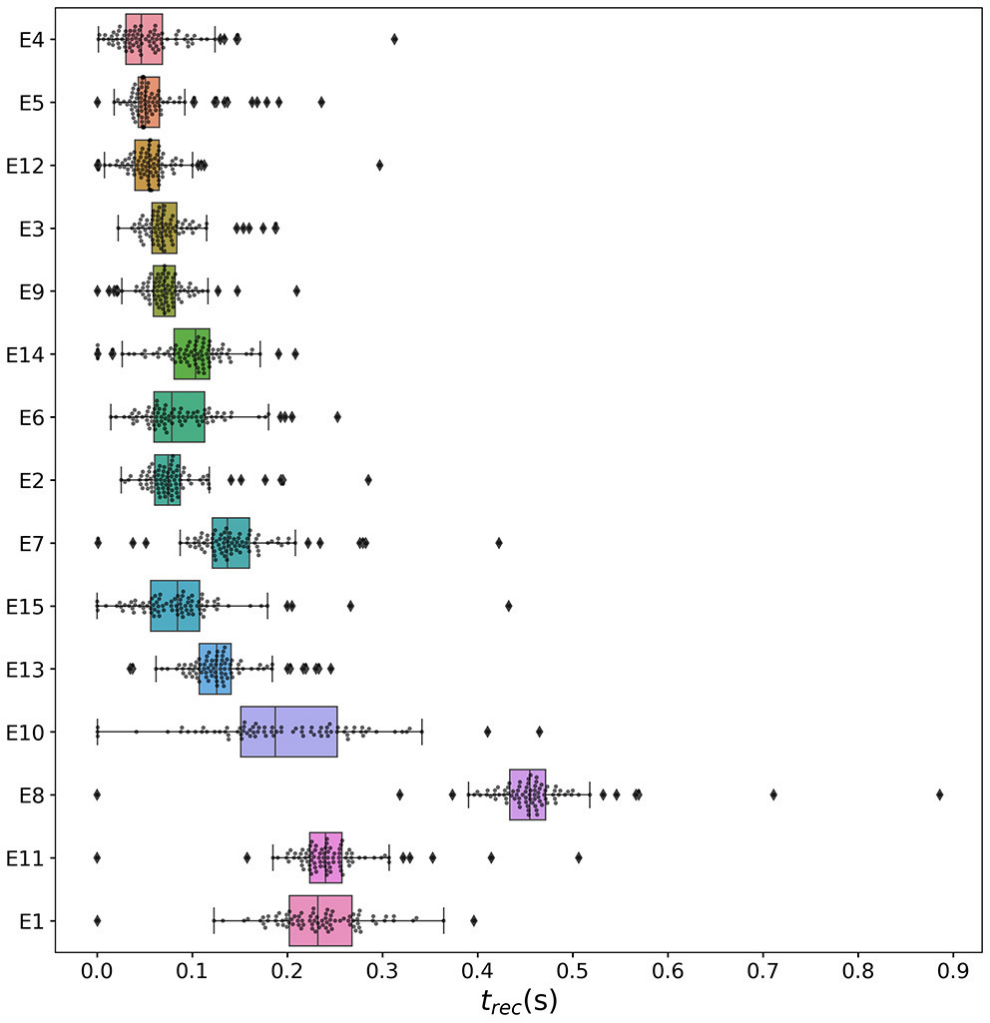
Recruitment times of all brain areas and all patients. The patients are sorted from top to bottom according to their median shortest path length, calculated by listing all the shortest path lengths of all areas to the EZ and then locating the number in the center of that distribution. Gray dots and diamonds show individual recruitments (we use two different symbols to highlight those values that are beyond the boxplot whiskers); boxes cover the 2nd and 3rd quartile and whiskers extend 1.5 times the IQR (whiskers are asymmetric, comprising the most extreme observed values that are within 1.5 × IQR from the upper or lower quartiles). Parameters as in Figure 10.

To conclude this section on the influence of single connectome topology in determining activity spreading and area recruitment, we elaborate the data reported in Figure 10 by sorting, from top to bottom, the patients according to their median shortest path length, calculated on all areas with respect to the EZ. In Figure 14 are shown the recruitment times of all brain areas for all patients. Since patients are ordered according to their median shortest path length, the brain areas of E4 have, on average, the shortest paths to the EZ and the areas of E1 the longest. In general, it is possible to detect a slight trend, for the overall recruitment events, to delay with longer average shortest path lengths. More in detail, E10 and E8 show both very long and very short recruitment times, thus confirming the results obtained in Figure 11 for Gaussian-distributed excitabilities. The scattering of the recruitment times for these patients reflects that, on average, their recruitment times are longer with respect to the other patients. However, the mean recruitment times are comparable with those of E11, E1, which show comparatively late recruitments irrespectively of the fact that are characterized by a longer median shortest path. A common characteristic that brings together patients E10, E8, E11, and E1 is the weak connection among the EZ and the first recruited area, that slows down the recruitment time (as already mentioned when discussing Figure 12), thus suggesting that is the interplay between connection strength and shortest path to determine the efficacy of seizure spreading and not the single topology measure alone.

#### 3.2.4. The Impact of the Input Current Strength on the Recruitment Time

**Figure 15:**
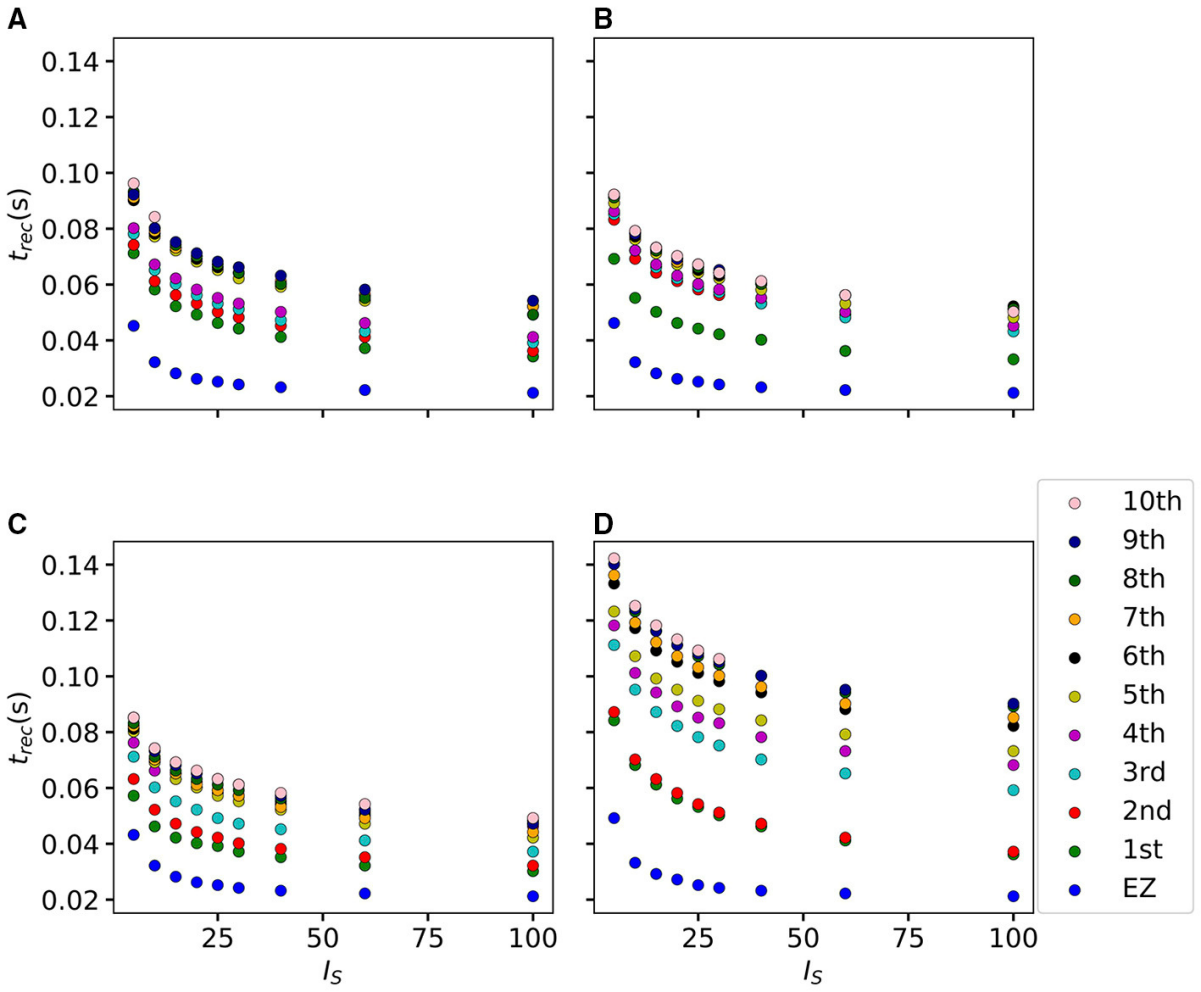
Recruitment times of the first 10 recruited areas as a function of the input current *I*_*S*_ for the epileptic patients **(A)** E2, **(B)** E3, **(C)** E6, and **(D)** E13. The strength of the input current is varied between 0 and 100 on the *x*-axis, while its duration is kept unchanged at *t*_*I*_ = 0.4 s with respect to the previous numerical experiments. The order of the recruitment is color-coded for each current strength (i.e., blue dots indicate the recruitment of the EZ, green dots indicate the first recruited area, red the second, etc.), and it holds the same for all investigated patients. Parameters as in Figure 10.

Following the same approach used to obtain the results shown in Figure 7 for a healthy subject, we present here an analysis on the impact of the stimulation strength on the recruitment mechanism. Figure 15 displays the recruitment times of the first 10 recruited areas using different amplitudes *I*_S_ of the step current *I*_S_(*t*), while fixing the duration *t*_I_ = 0.4 s. The analysis has been performed for patients E2 (Figure 15A), E3 (Figure 15B), E6 (Figure 15C), and E13 (Figure 15D), thus integrating the information on the dependency on topological measures presented in the previous section. As expected, the recruitment times decrease for larger amplitudes. However, the order of recruitment does not substantially change. This implies that, whenever we increase the amplitude, the recruitment mechanism remains unaffected: the same populations are involved in the seizure spreading and in the same order. What changes is the speed of the spreading and the time necessary to observe a generalized seizure-like event, which is smaller for stronger currents. As a general remark, the brain areas that are recruited after the first ones (i.e., the 5th, 6th,.,10th recruited areas), tend to be recruited more simultaneously for increasing *I*_S_, thus leading to possible changes in the recruitment order. This can be appreciated especially for patient E2: for an amplitude *I*_S_ = 10, for example, the 10th brain area (pink) gets recruited later than the 9th area (dark blue), while for very strong currents (*I*_S_ = 100), the dark blue area gets recruited latest whereas the pink area gets recruited earlier.

On the other hand, if we vary the step current duration *t*_I_ keeping the amplitude *I*_S_ = 15 fixed, we do not observe any change in the recruitment times of the first 10 recruited areas, analogously to the healthy subject case presented in Supplementary Figure 4. Results are shown in the Supplementary Figure 11.

## 4. Discussion

Neural mass models have been actively used since the 1970s to model the coarse-grained activity of large populations of neurons and synapses (Wilson and Cowan, 1972; Zetterberg et al., 1978). They have proven especially useful in understanding brain rhythms (Da Silva et al., 1974, 1976; Sotero et al., 2007), epileptic dynamics (Jirsa et al., 2014; Wendling et al., 2016), brain resonance phenomena (Spiegler et al., 2011), resting state (Ghosh et al., 2008; Deco et al., 2011), task activity (Huys et al., 2014; Kunze et al., 2016), and neurological and psychiatric disorders (Bhattacharya and Chowdhury, 2015) and are very popular in the neuroimaging community (Valdes-Sosa et al., 2009; Moran et al., 2013). Moreover, the desire to understand large scale brain dynamics as observed using EEG, MEG, and fMRI has prompted the increasing use of computational models (Bojak and Breakspear, 2014). Large-scale simulators such as The Virtual Brain (Sanz-Leon et al., 2015) and research infrastructures such as EBRAINS (http://ebrains.eu) make heavy use of networks of interconnected neural mass models and enable non-expert users to gain access to expert state-of-the-art brain network simulation tools.

Although motivated by neurobiological considerations, neural mass models are phenomenological in nature, and cannot hope to recreate some of the rich repertoires of responses seen in real neuronal tissue. In particular, their state variables track coarse-grained measures of the population firing rate or synaptic activity. At best they are expected to provide appropriate levels of description for many thousands of near, identical interconnected neurons with a preference to operate in synchrony, but they cannot reproduce the variation of synchrony within a neuronal population which is believed to underlie the decrease or increase of power seen in given EEG frequency bands. Importantly, unlike its phenomenological counterpart, the next-generation neural mass model we have implemented in this study, is an exact macroscopic description of an underlying microscopic spiking neurodynamics, and is a natural candidate for use in future large scale human brain simulations. In addition to this, the inability of a single neural mass model to support event-related desynchronization/synchronization (Pfurtscheller and Da Silva, 1999) or to capture the onset of synchronous oscillations in networks of inhibitory neurons (Devalle et al., 2017), reminds us that these phenomenological models could be improved upon. While building more detailed biophysically realistic models of neurons would increase the computational complexity and the difficulties to interpret the behavior of very high dimensional models in a meaningful way, the next-generation neural mass models applied in this study, are very much in the original spirit of neural mass modeling, yet importantly they can be interpreted directly in terms of an underlying spiking model. This exact derivation is possible for networks of quadratic integrate-and-fire neurons, representing the normal form of Hodgkin's class I excitable membranes (Ermentrout and Kopell, 1986), thanks to the analytic techniques developed for coupled phase oscillators (Ott and Antonsen, 2008). This new generation of neural mass models has been recently used to describe the emergence of collective oscillations in fully coupled networks (Devalle et al., 2017; Laing, 2017; Coombes and Byrne, 2019; Dumont and Gutkin, 2019) and in balanced sparse networks (di Volo and Torcini, 2018). Furthermore, it has been successfully employed to reveal the mechanisms at the basis of theta-nested gamma oscillations (Ceni et al., 2020; Segneri et al., 2020) and the coexistence of slow and fast gamma oscillations (Bi et al., 2020). Finally, it has been recently applied to modeling electrical synapses (Montbrió and Pazó, 2020), working memory (Taher et al., 2020), the influence of transcranial magnetic stimulation on brain dynamics (Byrne et al., 2020), and brain resting state activity (Rabuffo et al., 2020).

In this, we have extended the single next-generation neural mass model derived in Montbrió et al. (2015) to a network of interacting neural mass models, where the topology is determined by structural connectivity matrices of healthy and epilepsy-affected subjects. In this way, we can take into account both the macroscopic dynamics, self-emergent in the system due to the interactions among nodes, and the differences related to the patient-specific analyses. However, the single population neural mass model does not take into account neither the synaptic kinetics nor the dynamics of the synaptic field characterizing the considered synapses, which is simply modeled as the linear superposition of δ-shaped post-synaptic potentials. Moreover, when extending the (excitatory) neural mass model derived in Montbrió et al. (2015) to a multipopulation network, we have considered only excitatory coupling to build a minimal model for the investigation of topologically-induced dynamical features. Therefore, the presented neural mass model is not able to reproduce depth-EEG epileptic signals, which represents one of the best successes of heuristic neural mass models (Wendling et al., 2002).

In absence of external forcing, the phase diagram of the system as a function of the mean external drive $\bar{\eta }$ and synaptic strength σ resembles that of the single neural mass model, since the same distinct regions can be observed: (1) a single stable node corresponding to a low-activity state, (2) a single stable focus (spiral) generally corresponding to a high-activity state, and (3) a region of bistability between low and high firing rates. However, when the system is subject to a transient external current, the scenario changes and is ruled by the interactions among different nodes. In this case, for low excitability values, a single stimulated node abandons the bistable region due to the applied current and it approaches, with damped oscillations, the high-activity state, which is a stable focus. On the other hand, for sufficiently high excitabilities, the single node stimulation leads to the recruitment of other brain areas that reach, as the perturbed node, the high-activity regime by showing damped oscillations. This activity mimicks a seizure-like event and enables the modeling of propagation and recruitment: the seizure-like event originates in the EZ (as a results of the stimulation) and propagates to the PZ, identified by the other regions where fast propagates the oscillatory activity. It is distinct from an actual seizure, which would require the emergence of self-sustained activity in the high-activity state (Jirsa et al., 2014; Saggio et al., 2017, 2020).

However, transient activity, like the proposed seizure-like events, can play a potentially important role in localizing tissue involved in the generation of seizure activity, if read in the framework of stimulation of human epileptic tissue with consequent induction of rhythmic, self-terminating responses on the EEG or electrocorticogram (ECoG) (Valentin et al., 2002; Flanagan et al., 2009; Jacobs et al., 2010). From the dynamical systems perspective, one can hypothesize that complex stimulus responses are due to a space-dependent induction of self-terminating, spatio temporal transients that are caused by brief perturbations in an excitable medium (Goodfellow et al., 2012). Accordingly, considering epileptic seizure dynamics as spatio-temporal patterns (Goodfellow et al., 2011; Baier et al., 2012) shifts attention on the self-organizing capabilities of spatio temporal brain networks, thus proposing an alternative explanatory framework for epileptiform EEG to the time-dependent modulation in system parameters (Kramer et al., 2005; Breakspear and Jirsa, 2007; Kim et al., 2009; Marten et al., 2009; Lopour and Szeri, 2010).

Moreover, perturbation experiments, like the stimulation of human tissue, turns out to be fundamental in the context of functional brain mapping, as an integral part of contemporary neurosurgery (Sagar et al., 2019). Surgical planning of the resection procedure depends substantially on the delineation of abnormal tissue, e.g., epileptic foci or tumor tissue, and on the creation of a functional map of eloquent cortex in the area close to the abnormal tissue. Traditionally, different methodologies have been used to produce this functional map: electrical cortical stimulation (Hara et al., 1991; Ojemann, 1991; Uematsu et al., 1992), functional MRI (Chakraborty and McEvoy, 2008), PET (Bittar et al., 1999; Meyer et al., 2003), magnetoencephalography (Ganslandt et al., 1999), evoked potentials (Dinner et al., 1986), or passive recordings of electrocorticographic signals (Brunner et al., 2009). In particular, ECoG activity recorded from subdural electrodes, placed during surgical protocols, reflect task-related changes (Crone et al., 1998a,b, 2001; Aoki et al., 1999, 2001; Sinai et al., 2005; Leuthardt et al., 2007; Miller et al., 2007): ECoG amplitudes in specific frequency bands carry substantial information about movement or language tasks and they usually increase with the task in the gamma (>40 Hz) band. Extending the presented multipopulation model, *via* the addition of synaptic dynamics and an inhibitory pool, to reproduce task-related change in ECoG activity, would be essential to extend its predictive power.

The spectrogram analysis has revealed that the recruitment process is characterized by high frequency γ oscillations, thus reproducing the high-frequency (γ-band) EEG activity typical of electrophysiological patterns in focal seizures of human epilepsy. Many hypotheses have been formulated on the origin of this fast activity: (i) the behavior of inhibitory interneurons in hippocampal or neocortical networks in the generation of gamma frequency oscillations (Jefferys et al., 1996; Whittington et al., 2000); (ii) the nonuniform alteration of GABAergic inhibition in experimental epilepsy (reduced dendritic inhibition and increased somatic inhibition) (Cossart et al., 2001; Wendling et al., 2002); (iii) the possible depression of GABA_*A,fast*_ circuit activity by GABA_*A,slow*_ inhibitory postsynaptic currents (Banks et al., 2000; White et al., 2000); (iv) the out of phase patterns of depolarizing GABAergic post-synaptic potentials onto pyramidal cells, generated by feed-forward activation of cortical interneurons (Shamas et al., 2018). In any case, high-frequency EEG waves originating from one or several brain regions are the most characteristic electrophysiological pattern in focal seizures of human epilepsy and can be observed, in the numerical experiments, both for healthy subjects and epileptic patients, though with a distinction: for the same excitability value, the activity takes place at higher frequency ranges in epileptic patients and it is mainly concentrated in the EZ. Moreover, high-frequency γ oscillations (>200 Hz) are observable in the spectrogram of epileptic patients only. Even though it is not possible to exclude discrepancies partially imputable to the different scanning and preparation procedure of the structural connectivity matrices for the cohort of healthy and epilepsy-affected subjects, it turns out that the recruitment process is faster in epileptic patients, for which it is possible to observe generalized seizure-like events for smaller values of the excitability parameter $\bar{\eta }$. In particular, when comparing the results obtained for healthy subjects and epileptic patients, it turns out that the time necessary to recruit areas in the PZ is usually smaller for epileptic patients. However, the first recruited area is, in general, the area with the stronger connection to the EZ, independently of the considered structural connectivity matrix. The recruitment time in both cases is influenced by the strength of the external perturbation *I*_*S*_, and decreases for increasing strength, while no dependence is shown on the duration of the external perturbation.

More specifically for healthy subjects, we have investigated the dependence of the recruitment mechanism on the single subject, in terms of the position of the eventual EZ and the topological measures of the single connectome. Brain network models of healthy subjects comprise 90 nodes equipped with region-specific next-generation neural mass models and each subject is characterized by a specific structural large-scale connectivity amongst brain areas. The smallest excitability values for which an asymptomatic seizure-like event occurs (${\bar{\eta }}_{\text{asy}}^{\left(k\right)}$) do not vary significantly from one subject to the other and do not show a relevant dependence on the stimulated area, while the smallest excitability values for which a generalized seizure-like event occurs, (${\bar{\eta }}_{\text{gen}}^{\left(k\right)}$), show fluctuations in the interval (−7, −5) for all stimulated nodes and for all the subjects. Nonetheless, we have found many similarities at the level of topological measures, since there is always a strong correlation between ${\bar{\eta }}_{\text{asy}}^{\left(k\right)}$ (${\bar{\eta }}_{\text{gen}}^{\left(k\right)}$) and node strength, clustering coefficient and shortest path, thus meaning that a region well connected is a region well recruited.

For epileptic patients, we have systematically simulated the individual seizure-like propagation patterns and validated the numerical predictions of the PZ against clinical diagnosis and SEEG signals. Patient-specific brain network models of epileptic patients comprise 88 nodes equipped with region-specific next-generation neural mass models, and for this set up, we have studied the role of the large-scale connectome based on dMRI, in predicting the recruitment of distant areas through seizure-like events originating from a focal epileptogenic network. We have demonstrated that simulations and analytical solutions approximating the large-scale brain network model behavior significantly predict the PZ as determined by SEEG recordings and clinical expertise, with performances comparable to previous analyses on this set of data (Proix et al., 2017; Olmi et al., 2019), thus confirming the relevance of using a large-scale network modeling to predict seizure recruitment networks. However, some false positives are still observable, where populations not belonging to PZ_SEEG_ or PZ_Clin_ are first recruited. In these cases, the analysis on topological properties has revealed that nodes are easily recruited whenever they show strong connections to the EZ or too early recruited areas and that are closer to the EZ in terms of the shortest path length. Therefore, nodes belonging to the PZ_SEEG_ (PZ_Clin_), that are not identified by the simulations as first recruited nodes, are characterized by intermediate values of connection strength and shortest path. Predictions are particularly not good for those patients whose EZ has not been correctly identified, as results from the relative surgical outcomes reported in Supplementary Table 3. For these patients, the incorrect identification of the origin of seizure-like events may lead to a misleading identification of the PZ, since we are not able to identify, numerically, the recruitment of nodes not directly connected with the real EZ. Finally, comparing the results obtained for epileptic patients with those for healthy subjects, we infer a strong correlation between fast recruitment events and node strength, which is due to the fact that structural connectomes, both for healthy subjects and epileptic patients, are characterized by a log-normal distribution of the weights, where some connections, for each node, have a much stronger weight than the others. Moreover, the strong correlation between fast recruitment and clustering coefficient/shortest path suggests that we are in the presence of hierarchical connectivities, which are important for the spreading of activity (Kaiser et al., 2007; Luccioli et al., 2014) and the enhancement of the network susceptibility to seizure activity (Morgan and Soltesz, 2008).

Most computational models of seizure propagation focus on small continuous spatial scales (Ursino and La Cara, 2006; Kim et al., 2009; Hall and Kuhlmann, 2013) or population of neurons (Miles et al., 1988; Golomb and Amitai, 1997; Compte et al., 2003; Bazhenov et al., 2008; Chouzouris et al., 2018; Lopes et al., 2019; Gerster et al., 2020), while only small networks are commonly used to investigate the role of the topology and localization of the EZ (Terry et al., 2012). However, functional, volumetric and electrographic data suggest a broad reorganization of the networks in epileptic patients (Lieb et al., 1987, 1991; Cassidy and Gale, 1998; Rosenberg et al., 2006; Bettus et al., 2009), thus laying the foundations for a different approach based on large-scale connectomes to identify the recruitment networks. The large-scale character of partial seizure propagation in the human brain has been only recently investigated, using patient-specific dMRI data to systematically test the relevance of the large-scale network modeling, in predicting seizure recruitment networks (Proix et al., 2014, 2017, 2018; Olmi et al., 2019). In this framework of large-scale network modeling we can also place the results presented in this study, since we have confirmed the importance of patient-specific connectomes to identify the recruitment process. As shown above, the topological characteristics of connection strength and shortest path play a non-trivial role in determining the spreading of seizure-like events, together with the localization of the EZ, while the next-generation neural mass model, employed for the first time to study seizure spreading, allows us to construct patient-specific brain models *via* a multiscale approach: the variability of brain regions, as extracted from the human brain atlas, can be introduced in the mean-field parameters, thanks to the exact correspondence between microscopic and macroscopic scales guaranteed by the model itself. The possibility to exactly move through the scales has not been fully exploited in this study, since we have focused the analysis on the extension of the single neural mass model to a multipopulation model, without adding other relevant features to the original model. However, it is possible to easily introduce, in the multipopulation model, biologically relevant characteristics, keeping intact the exact correspondence between microscopic and macroscopic scales, such as short-term synaptic plasticity (Taher et al., 2020), synaptic delays (Devalle et al., 2018), electrical coupling *via* gap junctions (Montbrió and Pazó, 2020), chemical synapses (Coombes and Byrne, 2019), and extrinsinc and endogenous noise (Goldobin et al., 2021). By adding short-term synaptic plasticity we expect to be able to reproduce the emergence of self-sustained activity in the high-activity state and, therefore, to describe a fully developed seizure. The introduction of synaptic delays and noise guarantees the possibility to observe chaotic dynamics, therefore, allowing for the reproduction of more complex signals, like depth-EEG epileptic signals. Improving the predictive power of the model by the means of more biologically relevant characteristics and anatomical data (3D T1-weighted images, high angular and spatial dMRI data, ion, and energetic and neurotransmitter measurements available e.g., in the BigBrain and human brain atlas) will be the scope of further research.

## Data Availability Statement

All relevant data are within the paper and its Supplementary Material files. The simulated data supporting the conclusions of this article will be made available by the authors, without undue reservation. Numerical codes can be found on the open platform https://github.com/moritz-gerster/seizure_propagation. The healthy subject structural connectivity matrices used in this paper correspond to the first 20 subjects in a larger dataset. See https://osf.io/yw5vf/ for the data and a related paper describing the dataset in detail. Structural connectivity data for epileptic patients can be made available upon request following individual institutional requirements.

## Ethics Statement

The patients/participants provided their written informed consent to participate in research studies. For healthy subjects: The study design was approved by the local Ethics Committee of IKEM, Prague, Czech Republic. For epileptic patiens: Additional ethical review and approval was not required for this particular study in accordance with the local legislation and institutional requirements.

## Author Contributions

MGe and HT performed the simulations and data analysis, writing original software, and investigating the results. Data Curation is contributed by VJ, MGu, FB, AŠ, and JH. All the authors validated the research and participated in the drafting process. SO was responsible for conceptualization, supervision, state-of-the-art review (together with VJ), and the paper write-up.

## Conflict of Interest

The authors declare that the research was conducted in the absence of any commercial or financial relationships that could be construed as a potential conflict of interest.

## Publisher's Note

All claims expressed in this article are solely those of the authors and do not necessarily represent those of their affiliated organizations, or those of the publisher, the editors and the reviewers. Any product that may be evaluated in this article, or claim that may be made by its manufacturer, is not guaranteed or endorsed by the publisher.

## Footnotes

^1^While the actual role of the specific regions might in reality be affected by other factors, not captured by the used structural connectivity estimate and the details of the current model, this highlights the effect of network structure on propensity to seizure-like events. The (para)hippocampal region is, in fact, one of the most commonly affected by epilepsy.

^2^Please note that, irrespectively of the numerical results, any difference observed between the structural connectivity matrices obtained from the cohort of healthy subjects and epileptic patients may be (at least partially) ascribed to the different acquisition and processing procedures in the two research centers rather than due to disease-related causes.
