# Supplementary material for: Patient-Specific Network Connectivity Combined With a Next Generation Neural Mass Model to Test Clinical Hypothesis of Seizure Propagation
Source: Front Syst Neurosci. 2021 Sep 1;15:675272. doi: 10.3389/fnsys.2021.675272 (PMC8440880; doi:10.3389/fnsys.2021.675272)
Supplement: Supplementary file 1 [file Presentation_1.pdf]

Supplementary Material of the paper “Patient-specific network connectivity combined with a next generation neural mass model to test clinical hypothesis of seizure propagation”

Moritz Gerster [1], Halgurd Taher [2], Antonín Škoch [3], Jaroslav Hlinka [3,4], Maxime Guye [5,6], Fabrice Bartolomei [7], Viktor Jirsa [8], Anna Zakharova [1] and Simona Olmi [2,9,*]

[1] *Institut für Theoretische Physik, Technische Universität Berlin, Hardenbergstr. 36, 10623 Berlin*
[2] *Inria Sophia Antipolis Méditerranée Research Centre, MathNeuro Team, 2004 route des Lucioles-Boîte Postale 93 06902 Sophia Antipolis, Cedex, France*
[3] *National Institute of Mental Health, Topolová 748, 250 67 Klecany, Czech Republic*
[4] *Institute of Computer Science of the Czech Academy of Sciences, Pod Vodarenskou vezi 2, 18207 Prague 8, Czech Republic*
[5] *Faculté de Médecine de la Timone, centre de Résonance Magnétique et Biologique et Médicale (CRMBM, UMR CNRS-AMU 7339), Medical School of Marseille, Aix-Marseille Université, 13005, Marseille, France*
[6] *Assistance Publique -Hôpitaux de Marseille, Hôpital de la Timone, Pôle d'Imagerie, CHU, 13005, Marseille, France*
[7] *Assistance Publique - Hôpitaux de Marseille, Hôpital de la Timone, Service de Neurophysiologie Clinique, CHU, 13005 Marseille, France*
[8] *Aix Marseille Université, Inserm, Institut de Neurosciences des Systèmes, UMRS 1106, 13005, Marseille, France*
[9] *CNR - Consiglio Nazionale delle Ricerche - Istituto dei Sistemi Complessi, 50019, Sesto Fiorentino, Italy*

Correspondence*:
Simona Olmi
simona.olmi@fi.isc.cnr.it

1 MINIMAL BIOPHYSICAL MODELS OF SEIZURE DYNAMICS

The detection of epileptic seizures via electrophysiological recordings allowed for the establishment of a detailed taxonomy of seizures. The majority of seizures recorded in humans and experimental animal models can be described by a generic phenomenological mathematical model, the Epileptor (Jirsa et al., 2014). In this model, seizure events are driven by a slow permittivity variable and occur via saddle node and homoclinic bifurcations at seizure onset and offset, respectively. The saddle-node bifurcation at the onset of ictal discharges was chosen based on experimentally observed features, such as fixed frequency and fixed amplitude of abruptly starting oscillations, and a shift of baseline field potential. The homoclinic bifurcation

at the offset of ictal discharges, on the other hand, reproduces the logarithmic scaling of interspike intervals when approaching seizure offset. As part of the dynamic repertoire of the Epileptor, the epileptic attractor is described in terms of a self-sustained limit cycle that comes from the destabilisation of the physiological activity while multiple types of transitions allow for the accessibility of seizure activity, status epilepticus and depolarization block, that coexist, as verified experimentally in (El Houssaini et al., 2020).

The Epileptor model has been reduced to a minimal canonical mathematical representation of high codimension (up to 4) that, appropriately tuned, can display several types of fast-slow behaviors (Saggio et al., 2017). The model contains two subsystems acting at different time scales, in which the fast subsystem is unfolded in a plane showing several bifurcation paths of a high codimension singularity. The slow subsystem steers the fast one back and forth along these paths leading to fast-slow (aka bursting) behavior, mimicking epileptiform activity. The model is able to produce almost all the classes of bursting predicted for systems with a planar fast subsystem, including the Epileptor class, and has been demonstrated to be the dominant class, so-called dynamotype, in empirical epilepsy data (Saggio et al., 2020). Other dynamotypes have been also found empirically.

When performing the analysis of the single-population firing rate equations (4), it turns out that, in the absence of forcing, the only attractors are fixed points. As it becomes clear in the Section 3 of the Main Text, a stable node and a stable focus are observable, separated by a bistability region between a high- and a low-activity state, whose boundaries are the locus of a saddle-node bifurcation (for more details see (Montbrió et al., 2015)). In this context, self-sustained oscillations are not observable, but only damped oscillations at the macroscopic level that reflect the oscillatory decay to the stable fixed point. This oscillatory decay will here be considered as representative of a seizure-like event, not being able to observe a stable limit cycle to describe the emergence of a fully developed seizure as in the Epileptor. However, seizure-like events can be used as paradigm to investigate propagation of seizure-like activity in the network. Furthermore, a recently developed model of interictal and ictal discharges, called Epileptor-2 (Chizhov et al., 2018), makes links to underlying physiology and suggests how to eventually obtain all observed dynamotypes for the exact neural mass model Eqs. (4) and enable transitions towards fully developed seizure activity.

Epileptor-2 is a simple population-type model that includes four principal variables, i.e. the extracellular potassium concentration, the intracellular sodium concentration, the membrane potential and the synaptic resource diminishing due to short-term synaptic depression. A QIF neuron model, whose dynamics is ruled by an equation similar to Eq. (1), is used as an observer of the population activity. While the potassium accumulation governs the transition from the silent state to the state of ictal discharge, the sodium accumulated during the discharge, activates the sodium-potassium pump, which terminates the ictal discharge by restoring the potassium gradient, thus polarizing the neuronal membranes. This means that, in high potassium conditions, Epileptor-2 produces bursts of bursts, described as ictal-like discharges.

Therefore, the association of a slow subsystem describing ion concentration variations together with a fast subsystem, identified by Eqs. (4), should give rise to self-emergent periodic and bursting dynamics at the macroscopic level, thus allowing us to identify different combinations of onset/offset bifurcations. Whenever not sufficient, it will be possible to investigate the dynamics emergent in the exact neural mass model, provided with short-term synaptic plasticity, when subject to a global feedback acting on a slow timescale, describing ion concentration variations. The exact neural mass model, when equipped with short-term synaptic plasticity, shows a more complex dynamics that eventually results in a bifurcation diagram that provides stable limit cycles (Taher et al., 2020). However the introduction of short-term

plasticity, itself, adds complexity to the dynamics, allowing for the emergence of bursting activity (Tsodyks et al., 1998).

REFERENCES

Chizhov, A. V., Zefirov, A. V., Amakhin, D. V., Smirnova, E. Y., and Zaitsev, A. V. (2018). Minimal model of interictal and ictal discharges "epileptor-2". *PLoS computational biology* 14, e1006186

Desikan, R. S., Ségonne, F., Fischl, B., Quinn, B. T., Dickerson, B. C., Blacker, D., et al. (2006). An automated labeling system for subdividing the human cerebral cortex on mri scans into gyral based regions of interest. *Neuroimage* 31, 968–980

El Houssaini, K., Bernard, C., and Jirsa, V. K. (2020). The epileptor model: a systematic mathematical analysis linked to the dynamics of seizures, refractory status epilepticus and depolarization block. *Eneuro*

Jirsa, V. K., Stacey, W. C., Quilichini, P. P., Ivanov, A. I., and Bernard, C. (2014). On the nature of seizure dynamics. *Brain* 137, 2210–2230

Montbrió, E., Pazó, D., and Roxin, A. (2015). Macroscopic description for networks of spiking neurons. *Phys. Rev. X* 5, 021028

Saggio, M. L., Crisp, D., Scott, J. M., Karoly, P., Kuhlmann, L., Nakatani, M., et al. (2020). A taxonomy of seizure dynamotypes. *Elife* 9, e55632

Saggio, M. L., Spiegler, A., Bernard, C., and Jirsa, V. K. (2017). Fast–slow bursters in the unfolding of a high codimension singularity and the ultra-slow transitions of classes. *The Journal of Mathematical Neuroscience* 7, 1–47

Taher, H., Torcini, A., and Olmi, S. (2020). Exact neural mass model for synaptic-based working memory. *PLoS Computational Biology* 16, e1008533

Tsodyks, M., Pawelzik, K., and Markram, H. (1998). Neural networks with dynamic synapses. *Neural computation* 10, 821–835

Tzourio-Mazoyer, N., Landeau, B., Papathanassiou, D., Crivello, F., Etard, O., Delcroix, N., et al. (2002). Automated anatomical labeling of activations in SPM using a macroscopic anatomical parcellation of the MNI MRI single-subject brain. *Neuroimage* 15, 273–289

SUPPLEMENTARY TABLES AND FIGURES

Label	Region	Abbreviation	Label	Region	Abbreviation
1	Precentral Gyrus	PRE	46	Cuneus	Q
2	Precentral Gyrus	PRE	47	Lingual Gyrus	LING
3	Superior Frontal Gyrus	F1	48	Lingual Gyrus	LING
4	Superior Frontal Gyrus	F1	49	Superior Occipital Gyrus	O1
5	Superior Frontal Gyrus Orbital Part	F1O	50	Superior Occipital Gyrus	O1
6	Superior Frontal Gyrus Orbital Part	F1O	51	Middle Occipital Gyrus	O2
7	Middle Frontal Gyrus	F2	52	Middle Occipital Gyrus	O2
8	Middle Frontal Gyrus	F2	53	Inferior Occipital Gyrus	O3
9	Middle Frontal Gyrus Orbital Part	F2O	54	Inferior Occipital Gyrus	O3
10	Middle Frontal Gyrus Orbital Part	F2O	55	Fusiform Gyrus	FUSI
11	Inferior Frontal Gyrus Opercular Part	F3OP	56	Fusiform Gyrus	FUSI
12	Inferior Frontal Gyrus Opercular Part	F3OP	57	Postcentral Gyrus	POST
13	Inferior Frontal Gyrus Triangular Part	F3T	58	Postcentral Gyrus	POST
14	Inferior Frontal Gyrus Triangular Part	F3T	59	Superior Parietal Gyrus	P1
15	Inferior Frontal Gyrus Orbital Part	F3O	60	Superior Parietal Gyrus	P1
16	Inferior Frontal Gyrus Orbital Part	F3O	61	Inferior Parietal Gyrus	P2
17	Rolandic Operculum	RO	62	Inferior Parietal Gyrus	P2
18	Rolandic Operculum	RO	63	Supramarginal Gyrus	SMG
19	Supplementary Motor Area	SMA	64	Supramarginal Gyrus	SMG
20	Supplementary Motor Area	SMA	65	Angular Gyrus	AG
21	Olfactory Cortex	OC	66	Angular Gyrus	AG
22	Olfactory Cortex	OC	67	Precuneus	PQ
23	Superior Frontal Gyrus Medial	F1M	68	Precuneus	PQ
24	Superior Frontal Gyrus Medial	F1M	69	Paracentral Lobule	PCL
25	Superior Frontal Gyrus Medial Orbital	F1MO	70	Paracentral Lobule	PCL
26	Superior Frontal Gyrus Medial Orbital	F1MO	71	Caudate Nucleus	CAU
27	Gyrus Rectus	GR	72	Caudate Nucleus	CAU
28	Gyrus Rectus	GR e	73	Putamen	PUT
29	Insula	IN	74	Putamen	PUT
30	Insula	IN	75	Pallidum	PAL
31	Anterior Cingulate and paracingulate gyri	ACIN	76	Pallidum	PAL
32	Anterior Cingulate and paracingulate gyri	ACIN	77	Thalamus	THA
33	Median Cingulate and paracingulate gyri	MCIN	78	Thalamus	THA
34	Median Cingulate and paracingulate gyri	MCIN	79	Heschl Gyrus	HES
35	Posterior Cingulate Gyrus	PCIN	80	Heschl Gyrus	HES
36	Posterior Cingulate Gyrus	PCIN	81	Superior Temporal Gyrus	T1
37	Hippocampus	HIP	82	Superior Temporal Gyrus	T1
38	Hippocampus	HIP	83	Heschl Gyrus	HES
39	ParaHippocampal Gyrus	PHIP	84	Temporal Pole: superior temporal gyrus	T1P
40	ParaHippocampal Gyrus	PHIP	85	Temporal Pole: superior temporal gyrus	T1P
41	Amygdala	AMYG	86	Temporal Mid	T2
42	Amygdala	AMYG	87	Temporal Mid	T2
43	Calcarine fissure and surrounding cortex	V1	88	Temporal Pole: middle temporal gyrus	T2P
44	Calcarine fissure and surrounding cortex	V1	89	Middle Temporal Gyrus	T2
45	Cuneus	Q	90	Inferior Temporal Gyrus	T3

Table 1. Cortical and subcortical regions, according to the Automated Anatomical Labeling atlas 1(AAL1) (Tzourio-Mazoyer et al., 2002). Odd/even numbers correspond to the left/right hemisphere.

Label	Region	Abbreviation	Label	Region	Abbreviation
1	Unknown				
2	Brain-Stem				
3	Left-Cerebellum Cortex		46	Right-Cerebellum-Cortex	
4	Left-Thalamus Proper	lh-Th	47	Right-Thalamus Proper	rh-Th
5	Left-Caudate	lh-Cd	48	Right-Caudate	rh-Cd
6	Left-Putamen	lh-Pu	49	Right-Putamen	rh-Pu
7	Left-Pallidum	lh-Pal	50	Right-Pallidum	rh-Pal
8	Left-Hippocampus	lh-Hi	51	Right-Hippocampus	rh-Hi
9	Left-Amygdala	lh-Amg	52	Right-Amygdala	rh-Amg
10	Left-Accumbens-Area		53	Right-Accumbens Area	
11	Left-unknown		54	Right-unknown	
12	Left-bankssts		55	Right-bankssts	
13	Left-Caudal Anterior Cingulate	lh-CACC	56	Right-Caudal Anterior Cingulate	rh-CACC
14	Left-Caudal Middle Frontal	lh-CMFG	57	Right-Caudal Middle Frontal	rh-CMFG
15	Left-Cuneus	lh-Cun	58	Right-Cuneus	rh-Cun
16	Left-Entorhinal Cortex	lh-EntC	59	Right-Entorhinal cortex	rh-EntC
17	Left-Fusiform Gyrus	lh-FuG	60	Right-Fusiform Gyrus	rh-FuG
18	Left-Inferior Parietal Cortex	lh-IPC	61	Right-Inferior Parietal Cortex	rh-IPC
19	Left-Inferior Temporal Gyrus	lf-ITG	62	Right-Inferior Temporal Gyrus	rh-ITG
20	Left-Isthmus Cingulate Cortex	lh-ICC	63	Right-Isthmus Cingulate Cortex	rh-ICC
21	Left-Lateral Occipital Cortex	lh-LOCC	64	Right-Lateral Occipital Cortex	rh-LOCC
22	Left-Lateral Orbito Frontal Cortex	lh-LOFC	65	Right-Lateral Orbito Frontal Cortex	rh-LOFC
23	Left-Lingual Gyrus	lf-LgG	66	Right-Lingual Gyrus	rh-LgG
24	Left-Medial Orbito Frontal Cortex	lh-MOFC	67	Right-Medial Orbito Frontal Cortex	rh-MOFC
25	Left-Middle Temporal Gyrus	lh-MTG	68	Right-Middle Temporal Gyrus	rh-MTG
26	Left-Parahippocampal Gyrus	lh-PHiG	69	Right-Parahippocampal Gyrus	rh-PHiG
27	Left-Paracentral Cortex	lh-PaC	70	Right-Paracentral Cortex	rh-PaC
28	Left-Pars Opercularis	lh-Pop	71	Right-Pars Opercularis	rh-Pop
29	Left-Pars Orbitalis	lh-POr	72	Right-Pars Orbitalis	rh-POr
30	Left-Pars Triangularis	lh-PT	73	Right-Pars Triangularis	rh-PT
31	Left-Pericalcarine	lh-PC	74	Right-Pericalcarine	rh-PC
32	Left-Postcentral Gyrus	lh-PoG	75	Right-Postcentral Gyrus	rh-PoG
33	Left-Posterior Cingulate Gyrus	lh-PCG	76	Right-Posterior Cingulate Gyrus	rh-PCG
34	Left-Precentral Gyrus	lh-PrG	77	Right-Precentral Gyrus	rh-PrG
35	Left-Precuneus Cortex	lh-PCunC	78	Right-Precuneus Cortex	rh-PCunC
36	Left-Rostral Anterior Cingulate Cortex	lh-RACC	79	Right-Rostral Anterior Cingulate Cortex	rh-RACC
37	Left-Rostral Middle Frontal Gyrus	lh-RMFG	80	Right-Rostral Middle Frontal Gyrus	rh-RMFG
38	Left-Superior Frontal Gyrus	lh-SFG	81	Right-Superior Frontal Gyrus	rh-SFG
39	Left-Superior Parietal Cortex	lh-SPC	82	Right-Superior Parietal Cortex	rh-SPC
40	Left-Superior Temporal Gyrus	lh-STG	83	Right-Superior Temporal Gyrus	rh-STG
41	Left-Supramarginal Gyrus	lh-SMG	84	Right-Supramarginal Gyrus	rh-SMG
42	Left-Frontal Pole	lh-FP	85	Right-Frontal Pole	rh-FP
43	Left-Temporal Pole	lh-TmP	86	Right-Temporal Pole	rh-TmP
44	Left-Transverse Temporal Pole	lh-TTmP	87	Right-Transverse Temporal Pole	rh-TTmP
45	Left-Insula	lh-Ins	88	Right-Insula	rh-Ins

Table 2. Cortical and subcortical regions, according to the Desikan-Killiany atlas (Desikan et al., 2006).

Patient	Gender	Epilepsy duration (years)	Age at seizure onset (years)	Epilepsy type	Surgical procedure	Surgical outcome	MRI	Histopathology	Side
E1	F	14	8	Temporo -frontal	Sr	III	Anterior temporal necrosis	Gliosis	R
E2	F	14	9	Occipital	Sr	III	N	FCD type 1	L
E3	M	35	7	Insular	GK	I	N	NA	L
E4	F	18	5	SMA	Sr	I	N	FDC type 2	L
E5	F	23	7	Parietal	Sr	I	FCD SPC	FCD type 2	L
E6	F	16	7	Premotor	Th	II	N	NA	R
E7	M	45	11	Temporo -frontal	Sr	I	FCD F	FCD type 2	R
E8	M	5	28	Temporal	Sr	III	Temporopolar hypersignal	FCD type 1	R
E9	F	18	20	Occipital	N	NO	N	NA	R
E10	M	11	18	Frontal	Sr	I	Frontal necrosis (post-trauma)	Gliosis	R
E11	F	10	17	Temporal	Gk	II	Hyppocampal sclerosis	NA	R
E12	M	15	14	Temporal	N	NO	N	NA	R
E13	M	29	7	Temporal	Sr	I	Cavernoma	Cavernoma	R
E14	M	28	35	Temporal	Sr	III	N	Gliosis	L
E15	F	24	4	Occipital	N	NO	PVH	NA	R

Table 3. Clinical characteristics of the patients. N, normal; L, left; R, right; Th, thermocoagulation; Gk, Gamma knife; Sr, surgical resection; NO, not operated; PVH, periventricular nodular heterotopia; FCD, focal cortical dysplasia; SPC, superior parietal cortex; F, Frontal; NA, not available.

Patient	EZ location	PZ location SEEG	PZ clinical prediction
E1	rLOFC, rTmP	rRMFG, lRMFG	rRMFG, rMOFC, rPOr, rIns rPu, rPT, rAccumbes
E2	lLOCC	lFuG, lPC, lSPC	lFuG, lSPC, lITG, lIPC, lPC, lLgG
E3	lIns	lPoG	lPu, lLOFC, lSMG, lPrG, lPop, lPoG
E4	lPCG, lCMFG, lSFG	lPrG, lSPC, lPoG	lRMFG, lPrG, rSFG, lCACC, lPaC
E5	lPCG, lPCunC	lPoG, lIPC	lICC, lIPC, lSPC
E6	rPrG	rCMFG	rPoG, rCMFG, rPop, rSFG rTh, rPu, rPaC, rSMG
E7	rAmg, rTmP, rLOFC	rFuG, lPHiG, rITG	rTmP, rIns, rPu, rMOFC, rPOr, rRMFG
E8	rAmg, rHi	rITG, rTmP	rPHiG, rEntC, rTmP, rFuG, rPal, rTh
E9	rLgG, rPHiG	rHi, rFuG, rIPC, rLOCC, rSPC, rITG	rFuG, rHi, rPC, rLOCC, rICC
E10	rMOFC, rFP, rRMFG, rPOr	rPop, rMTG, rLOFC	rSFG, rPT, rPrG, rCd, rPop, rPu
E11	rHi, rAmg	rLOFC, rMTG	rTh, rLOFC, rRACC, rIns rCd, rPu, rMOFC
E12	rHi, rFuG, rEntC, rTmP	lFuG, rITG	rITG, rLOCC, rLgG, rPHiG, rAmg
E13	rFuG	rEntC, rIPC, rHi	rITG, rLOCC, rTmP
E14	lAmg, lHi, lEntC, lFuG lTmP, rEntC	lMTG, rMTG, lIns	lITG, lLOCC, lPHiG, lLgG lCerebellum
E15	rLgG, rLOCC, rCun, rPC	lPCunC, lCun, rPHiG	rFuG, rIPC, rITG, rMTG, rSPC

Table 4. Results of propagation zone prediction for each patient. Abbreviations are given in Supplementary Table 2.

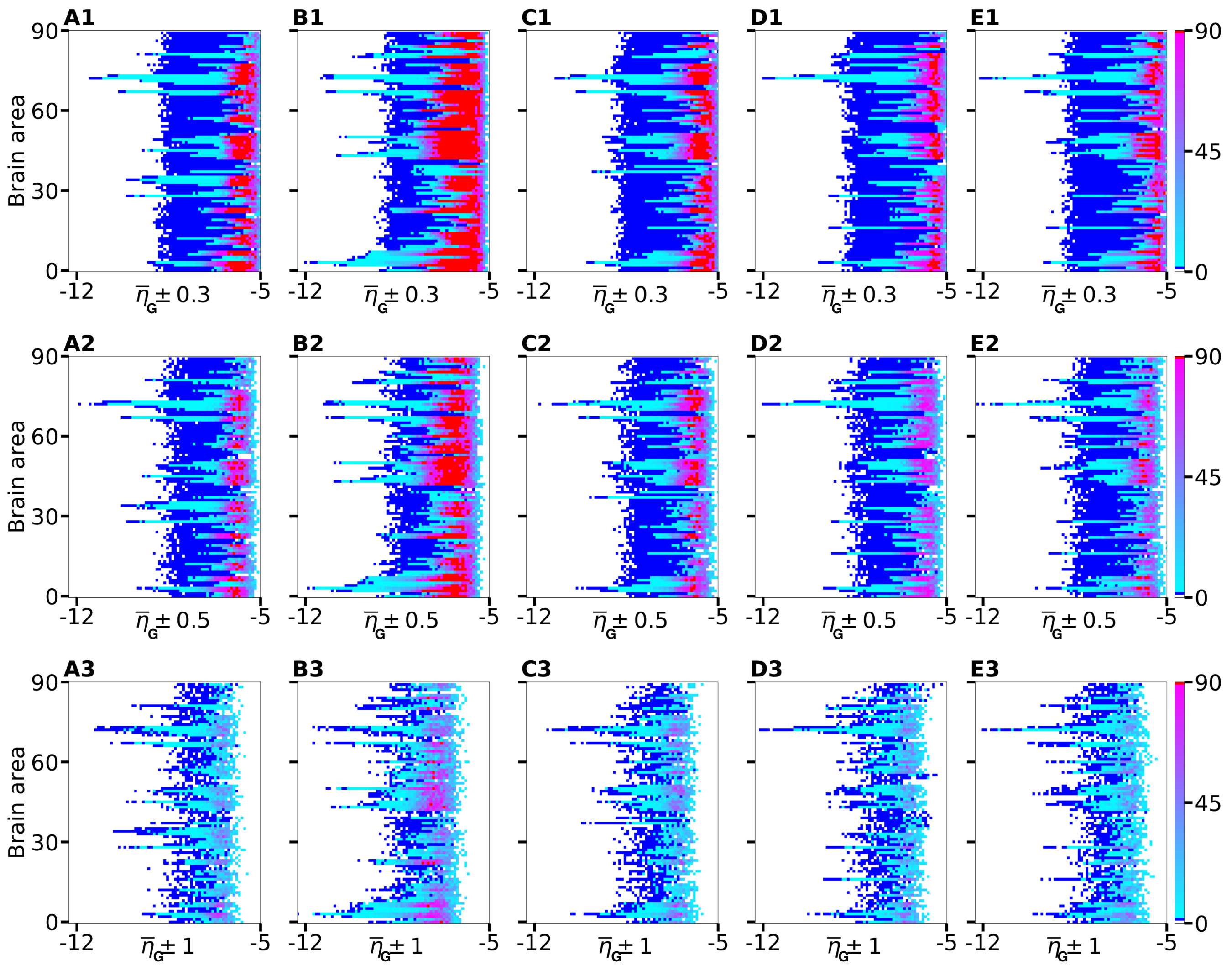

Figure 1. Number of recruited brain areas as a function of the excitability parameter $\bar{\eta}_{\mathrm{G}}$ for 5 exemplary healthy subject connectomes and increasing Gaussian standard deviation. Here is reported a numerical experiment equivalent to the one shown in Fig. 3 (Main Text), where $\bar{\eta}_{\mathrm{G}}$ represents the mean value of a Gaussian distribution with increasing standard deviations σ_{G}. (A1-E1) $\sigma_{\mathrm{G}} = 0.3$; (A2-E2) $\sigma_{\mathrm{G}} = 0.5$; (A3-E3) $\sigma_{\mathrm{G}} = 1$. Color coding is the following: blue corresponds to the asymptomatic threshold (one area in HA regime); red represents 90 areas in HA regime (generalized threshold); cyan to purple indicate intermediate recruitment values, white marks no recruitment. For very large values of $\bar{\eta}_G$ (usually $\bar{\eta}_G > -5$), the system enters the stable focus regime before the stimulation is applied. In that case $\bar{\eta}^{(k)}_{\mathrm{gen}}$ is not defined because no brain areas are recruited as a result of the applied stimulation current: for this reason white color, corresponding to no recruited areas, is visible also in the right part of the different panels. For increasing standard deviation values, the effective excitability of the more and more nodes turn out to be close to the one that allows the system to be in the stable focus regime, therefore the probability of finding nodes in the stable focus regime increases. As a result, more and more nodes enter the HA state and recruite the other areas before the stimulation current is applied. This means that for increasing standard deviation, $\bar{\eta}^{(k)}_{\mathrm{gen}}$ become less defined while $\bar{\eta}^{(k)}_{\mathrm{asy}}$ move to larger values of $\bar{\eta}_G$, still remaining better identifiable for increasing heterogeneity. As in Fig. 3, A), B), C), D), and E) correspond respectively to subjects H1, H5, H12, H16, and H19. Parameters: $N_{\mathrm{pop}} = 90$, $\Delta = 1$, $\sigma = 1$, $I_S = 10$, $t_I = 0.4$ s.

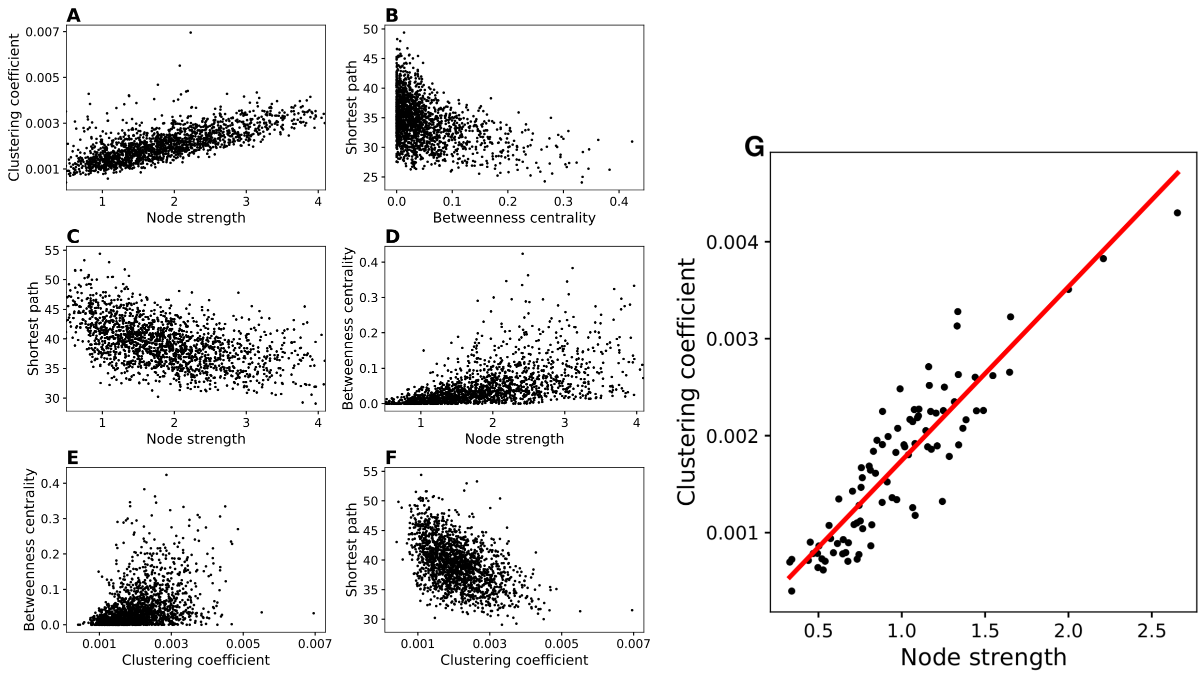

Figure 2. Network measure correlations of healthy subjects. Panels A-F are obtained plotting independently all node values for all the subjects (90*20=1800 data points). Panel G: Data are averaged over all 20 subjects. The single node values are averaged over the different subjects and afterwards, the correlation between node strength and clustering coefficient is estimated. Infinite values were excluded. The Pearson correlation of clustering coefficient and node strength of the averaged healthy DTI topology is $r = 0.9$ and much stronger compared to the average of all individual topologies $r = 0.75$ from panel A.

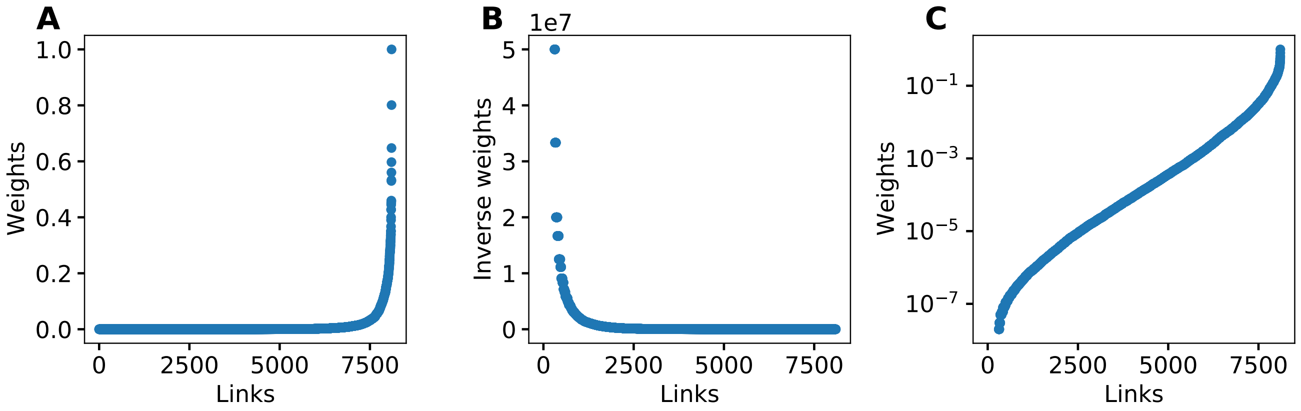

Figure 3. Weight Distribution of the DTI graphs. The weight distribution with weights on the x-axis in ascending order. a) Weight distribution, b) the inverse weight distribution, c) and the logarithmic weight distribution of the healthy averaged DTI graph. Note that c) matches the curve of recruitment times in Fig. 6 A) in the Main Text.

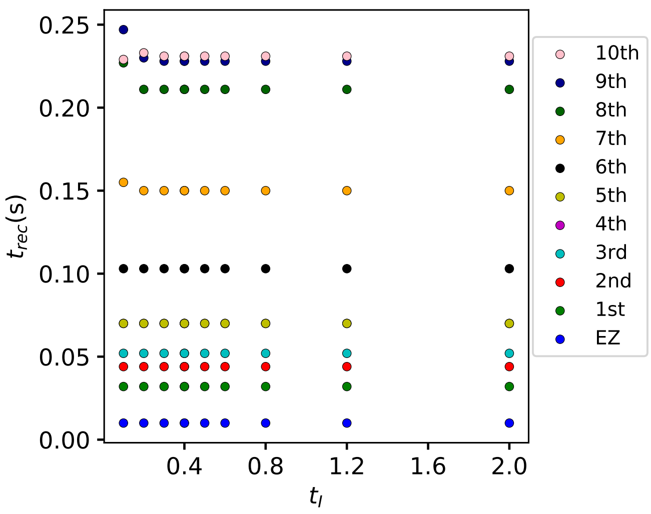

Figure 4. Input Current Duration Variation. Dependence of the recruitment time on the current duration t_I while the current strength is kept constant at $I_S = 15$. The y-axis shows the recruitment times of the first 10 recruited areas for each current strength. Blue is the EZ, green is the first recruited area, red the second, etc. The recruitment times are independent of the pulse duration. Parameters: $N_{\text{pop}} = 90$, $\sigma = 1$, $\Delta = 1$, $\bar{\eta} = -6$, $I_{\text{S}} = 15$, stimulation site: brain area $k = 45$ for the healthy H1.

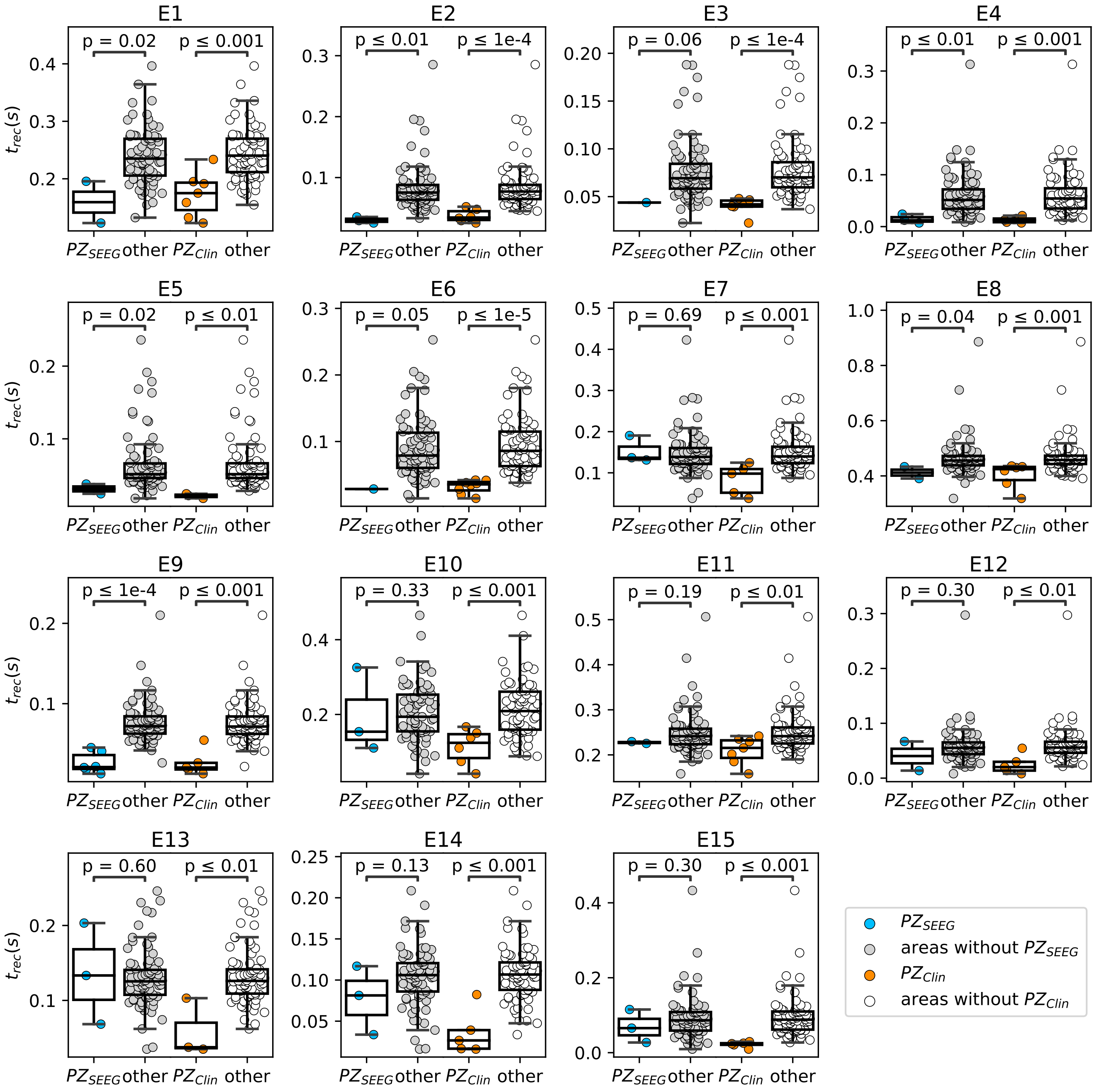

Figure 5. Statistical significance of PZ_{SEEG} and PZ_{clin} recruitment times. The figure shows the recruitment times of all brain areas on the y-axis. Blue (orange) dots correspond to PZ_{SEEG} (PZ_{clin}). The grey dots correspond to all brain areas except for the EZ and the areas identified as PZ_{SEEG}; the white dots to all brain areas except for the EZ and the areas identified as PZ_{Clin}. Note that some brain areas of the PZ are either identified as PZ_{SEEG} or PZ_{Clin} while others are identified as both PZ_{SEEG} and PZ_{Clin}. Therefore the set of grey and white dots is very similar but not identical. A one-sided Mann-Whitney U test detected significantly earlier recruitment of the PZ_{SEEG} ($p < 0.05$) for patients E1, E2, E4, E5, E8, and E9. For the PZ_{Clin}, the recruitment was significantly earlier for all patients.

Patient	Recruitment order	Recruitment time (s)	Type	Region
E1	0	0.0	EZ	rh-LOFC
E1	1	0.0005	EZ	rh-TmP
E1	2	0.123	PZ_{SEEG}, PZ_{Clin}	rh-RMFG
E1	3	0.1324	PZ_{Clin}	rh-Pu
E1	4	0.1546	other	rh-SFG
E1	5	0.1587	PZ_{Clin}	rh-Ins
E1	6	0.1718	other	rh-Pal
E1	7	0.1747	other	rh-PrG
E1	8	0.175	other	rh-MOFC
E1	9	0.1769	other	rh-Cd
E1	10	0.1797	other	lh-SFG
E1	11	0.1801	other	rh-PoG
E2	0	0.0	EZ	lh-LOCC
E2	1	0.0255	PZ_{SEEG}, PZ_{Clin}	lh-FuG
E2	2	0.0294	PZ_{SEEG}, PZ_{Clin}	lh-SPC
E2	3	0.0333	PZ_{Clin}	lh-ITG
E2	4	0.0354	PZ_{SEEG}, PZ_{Clin}	lh-IPC
E2	5	0.045	other	lh-MTG
E2	6	0.046	other	lh-SMG
E2	7	0.0466	other	lh-PCunC
E2	8	0.0479	PZ_{Clin}	lh-LgG
E2	9	0.0482	other	lh-PoG
E2	10	0.0521	other	lh-PrG
E3	0	0.0	EZ	lh-Ins
E3	1	0.0224	PZ_{Clin}	lh-Pu
E3	2	0.0369	other	lh-SFG
E3	3	0.0394	PZ_{Clin}	lh-LOFC
E3	4	0.04	PZ_{Clin}	lh-PrG
E3	5	0.0438	PZ_{SEEG}, PZ_{Clin}	lh-PoG
E3	6	0.0446	other	lh-RMFG
E3	7	0.0451	other	lh-Th
E3	8	0.0453	other	lh-CMFG
E3	9	0.0459	other	rh-SFG
E3	10	0.0465	PZ_{Clin}	lh-Pop
E4	0	0.0	EZ	lh-SFG
E4	1	0.0016	EZ	lh-CMFG
E4	2	0.0026	EZ	lh-PCG
E4	3	0.007	PZ_{SEEG}, PZ_{Clin}	lh-PrG
E4	4	0.0085	PZ_{Clin}	lh-RMFG
E4	5	0.0122	PZ_{SEEG}	lh-PoG
E4	6	0.0126	PZ_{Clin}	rh-SFG
E4	7	0.0155	PZ_{Clin}	lh-PaC
E4	8	0.0168	other	lh-Pop
E4	9	0.0168	other	lh-Pu
E4	10	0.0175	other	lh-Th
E4	11	0.0211	other	lh-SMG
E4	12	0.0212	PZ_{Clin}	lh-CACC
E5	0	0.0	EZ	lh-PCunC
E5	1	0.0006	EZ	lh-PCG
E5	2	0.0181	PZ_{Clin}	lh-SPC
E5	3	0.0219	PZ_{Clin}	lh-ICC
E5	4	0.0244	PZ_{SEEG}, PZ_{clin}	lh-IPC
E5	5	0.0284	other	lh-SFG
E5	6	0.0318	other	lh-LOCC
E5	7	0.0345	other	rh-SFG
E5	8	0.0363	other	lh-Cun
E5	9	0.0364	other	lh-SMG
E5	10	0.0366	other	lh-Th
E5	11	0.0374	other	lh-PrG

Table 5. List of the first 10 recruited brain areas for each patient. The column "Type" indicates whether the recruited area belongs to the estimated PZ via presurgical invasive (PZ_{SEEG}) or non-invasive (PZ_{Clin}) evaluation.

Patient	Recruitment order	Recruitment time (s)	Type	Region
E6	0	0.0	EZ	rh-PrG
E6	1	0.0146	PZ_{Clin}	rh-PoG
E6	2	0.02	PZ_{Clin}	rh-SFG
E6	3	0.0287	PZ_{SEEG}, PZ_{Clin}	rh-CMFG
E6	4	0.0342	PZ_{Clin}	rh-SMG
E6	5	0.0369	PZ_{Clin}	rh-Pop
E6	6	0.038	other	lh-SFG
E6	7	0.0382	PZ_{Clin}	rh-Th
E6	8	0.0396	other	rh-RMFG
E6	9	0.0417	PZ_{Clin}	rh-PaC
E6	10	0.042	PZ_{Clin}	rh-Pu
E7	0	0.0	EZ	rh-LOFC
E7	1	0.0012	EZ	rh-TmP
E7	2	0.0012	EZ	rh-Amg
E7	3	0.0379	PZ_{Clin}	rh-Ins
E7	4	0.0516	PZ_{Clin}	rh-Pu
E7	5	0.0875	other	rh-SFG
E7	6	0.0949	other	rh-PrG
E7	7	0.0954	other	rh-Pal
E7	8	0.098	PZ_{Clin}	rh-RMFG
E7	9	0.1027	other	rh-PoG
E7	10	0.1031	other	rh-CMFG
E7	11	0.1034	other	lh-SFG
E7	12	0.1067	other	rh-Th
E8	0	0.0	EZ	rh-Hi
E8	1	0.0003	EZ	rh-Amg
E8	2	0.3184	PZ_{Clin}	rh-PHiG
E8	3	0.3735	PZ_{Clin}	rh-FuG
E8	4	0.3905	PZ_{SEEG}	rh-ITG
E8	5	0.3965	other	rh-LOCC
E8	6	0.3999	other	rh-MTG
E8	7	0.4027	other	rh-LgG
E8	8	0.4097	other	rh-IPC
E8	9	0.4106	other	rh-STG
E8	10	0.4137	other	rh-PC
E8	11	0.4179	other	rh-bnks
E9	0	0.0	EZ	rh-LgG
E9	1	0.0006	EZ	rh-PHiG
E9	2	0.0128	PZ_{SEEG}, PZ_{Clin}	rh-FuG
E9	3	0.0181	PZ_{SEEG}, PZ_{Clin}	rh-Hi
E9	4	0.0205	PZ_{SEEG}, PZ_{Clin}	rh-LOCC
E9	5	0.0217	PZ_{SEEG}	rh-ITG
E9	6	0.0264	PZ_{Clin}	rh-PC
E9	7	0.0408	PZ_{SEEG}	rh-IPC
E9	8	0.0417	other	rh-MTG
E9	9	0.0453	PZ_{SEEG}	rh-SPC
E9	10	0.0483	other	rh-Th
E9	11	0.0483	other	rh-Cun
E10	0	0.0	EZ	rh-RMFG
E10	1	0.0006	EZ	rh-MOFC
E10	2	0.0008	EZ	rh-FP
E10	3	0.0008	EZ	rh-POr
E10	4	0.0414	PZ_{Clin}	rh-SFG
E10	5	0.0745	PZ_{Clin}	rh-PrG
E10	6	0.0882	other	rh-PoG
E10	7	0.0894	other	rh-CMFG
E10	8	0.0957	other	rh-SMG
E10	9	0.1035	other	rh-SPC
E10	10	0.1103	PZ_{SEEG}, PZ_{Clin}	rh-Pop
E10	11	0.1119	other	rh-IPC
E10	12	0.1155	other	rh-PaC

Table 6. Continued from Table 5.

Patient	Recruitment order	Recruitment time (s)	Type	Region
E11	0	0.0	EZ	rh-Hi
E11	1	0.0003	EZ	rh-Amg
E11	2	0.158	PZ_{Clin}	rh-Th
E11	3	0.185	PZ_{Clin}	rh-Cd
E11	4	0.19	other	rh-SFG
E11	5	0.1993	other	rh-RMFG
E11	6	0.2006	other	BS
E11	7	0.2015	PZ_{Clin}	rh-Pu
E11	8	0.2035	other	rh-Pal
E11	9	0.2072	other	rh-PrG
E11	10	0.2084	other	lh-SFG
E11	11	0.2123	other	rh-CMFG
E12	0	0.0	EZ	rh-FuG
E12	1	0.0006	EZ	rh-Hi
E12	2	0.0014	EZ	rh-EntC
E12	3	0.0016	EZ	rh-TmP
E12	4	0.008	PZ_{Clin}	rh-LOCC
E12	5	0.0137	PZ_{SEEG}, PZ_{Clin}	rh-ITG
E12	6	0.0202	PZ_{Clin}	rh-LgG
E12	7	0.0215	other	rh-MTG
E12	8	0.0243	other	rh-SPC
E12	9	0.0267	other	rh-IPC
E12	10	0.0288	other	rh-PC
E12	11	0.0298	PZ_{Clin}	rh-PHiG
E12	12	0.0324	other	rh-PCunC
E12	13	0.0325	other	rh-SMG
E13	0	0.0	EZ	rh-FuG
E13	1	0.035	PZ_{Clin}	rh-LOCC
E13	2	0.0375	PZ_{Clin}	rh-ITG
E13	3	0.062	other	rh-MTG
E13	4	0.0683	PZ_{SEEG}	rh-IPC
E13	5	0.0742	other	rh-SPC
E13	6	0.084	other	rh-STG
E13	7	0.0864	other	rh-SMG
E13	8	0.0903	other	rh-bnks
E13	9	0.0907	other	rh-PrG
E13	10	0.0922	other	rh-PoG
E14	0	0.0	EZ	lh-Hi
E14	1	0.0	EZ	lh-FuG
E14	2	0.0008	EZ	lh-EntC
E14	3	0.0008	EZ	lh-TmP
E14	4	0.0008	EZ	rh-EntC
E14	5	0.0008	EZ	lh-Amg
E14	6	0.016	PZ_{Clin}	lh-ITG
E14	7	0.0166	PZ_{Clin}	lh-PHiG
E14	8	0.0266	PZ_{Clin}	lh-LOCC
E14	9	0.0336	PZ_{SEEG}	lh-MTG
E14	10	0.0391	PZ_{Clin}	lh-LgG
E14	11	0.0474	other	lh-STG
E14	12	0.0504	other	lh-IPC
E15	0	0.0	EZ	rh-LOCC
E15	1	0.0003	EZ	rh-PC
E15	2	0.0003	EZ	rh-LgG
E15	3	0.0008	EZ	rh-Cun
E15	4	0.0095	PZ_{Clin}	rh-FuG
E15	5	0.021	PZ_{Clin}	rh-IPC
E15	6	0.024	PZ_{Clin}	rh-SPC
E15	7	0.0253	PZ_{Clin}	rh-ITG
E15	8	0.0272	PZ_{SEEG}	rh-PCunC
E15	9	0.0293	PZ_{Clin}	rh-MTG
E15	10	0.0338	other	rh-SMG
E15	11	0.0361	other	rh-bnks
E15	12	0.0374	other	rh-PoG

Table 7. Continued from Tables 5, 6.

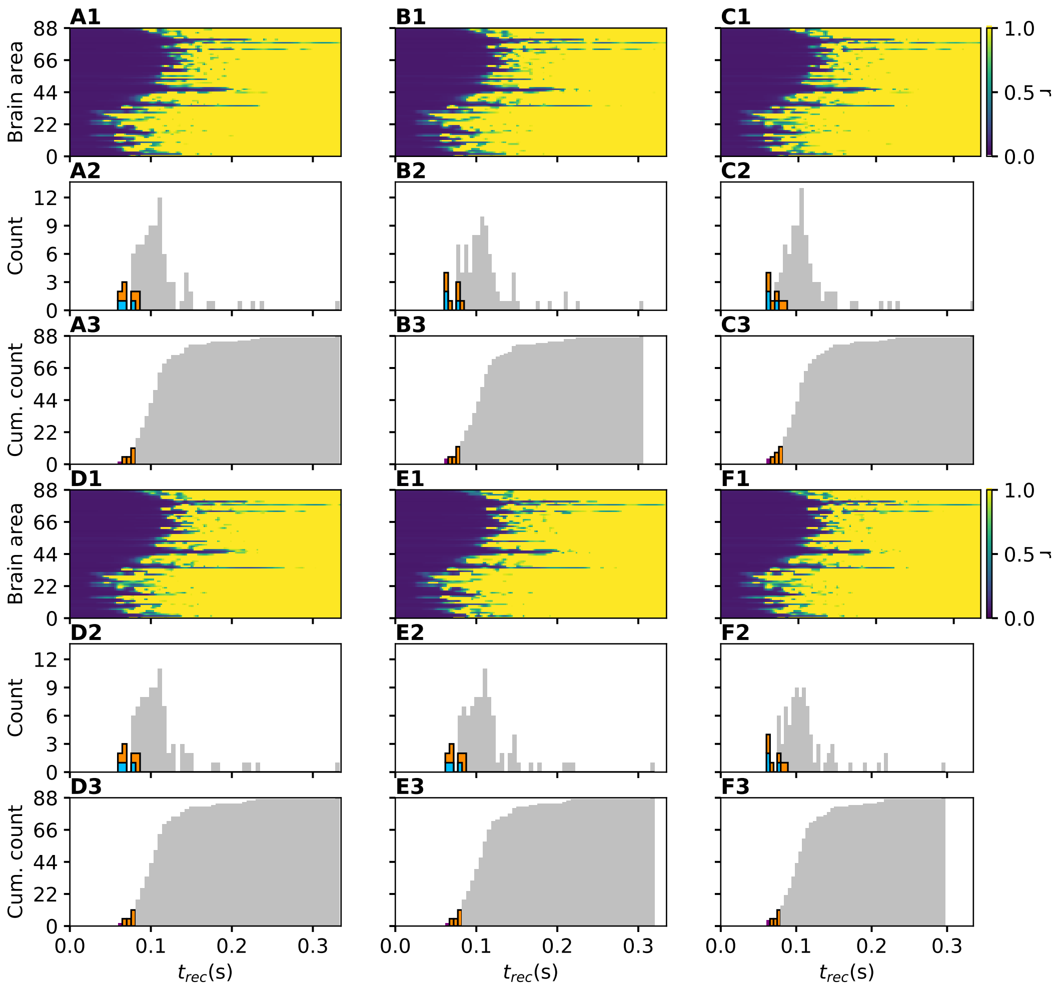

Figure 6. Recruitment times for patient E2 obtained for 6 different random Gaussian distributions of $\bar{\eta}$. (A1-F1) Spacetime plots of the average firing rates of all brain areas. (A2-F2) Histograms of the recruitment times. Orange (blue) bins identify those recruited area that belong to $\mathrm{PZ_{Clin}}$ ($\mathrm{PZ_{SEEG}}$). (A3-F3) Cumulative histograms of the recruitment times. Orange bins: first 10 recruited areas. Parameters as in Fig. 10 (Main Text). For one exemplary patient, E2, we show here in detail the impact of different realizations of $\bar{\eta}^{(k)}$, drawn from a Gaussian distribution (centred at $\bar{\eta}_G = -7.5$ with standard deviation 0.1), on the recruitment times of the brain areas. In particular we have considered it to be sufficient to present results for six out of ten realizations, due to the large similarities between the outcomes. Space-time plots of the average firing rates give an immediate visualization of the recruitment events for each brain area. We find that the pattern of recruitment does not change substantially for different realizations of the $\bar{\eta}^{(k)}$. The EZ is localized in the area lh-LOCC, that corresponds to node $k = 21$: The firing rate of this population increases immediately upon stimulation, thus giving rise to the recruitment mechanism. The brain areas in the PZ are rapidly recruited: In general the first ten areas are always recruited in less then 0.1s, followed by a continuous increase of the number of recruited nodes. Finally, it is worth noticing that the first recruited areas correspond to those predicted clinically.

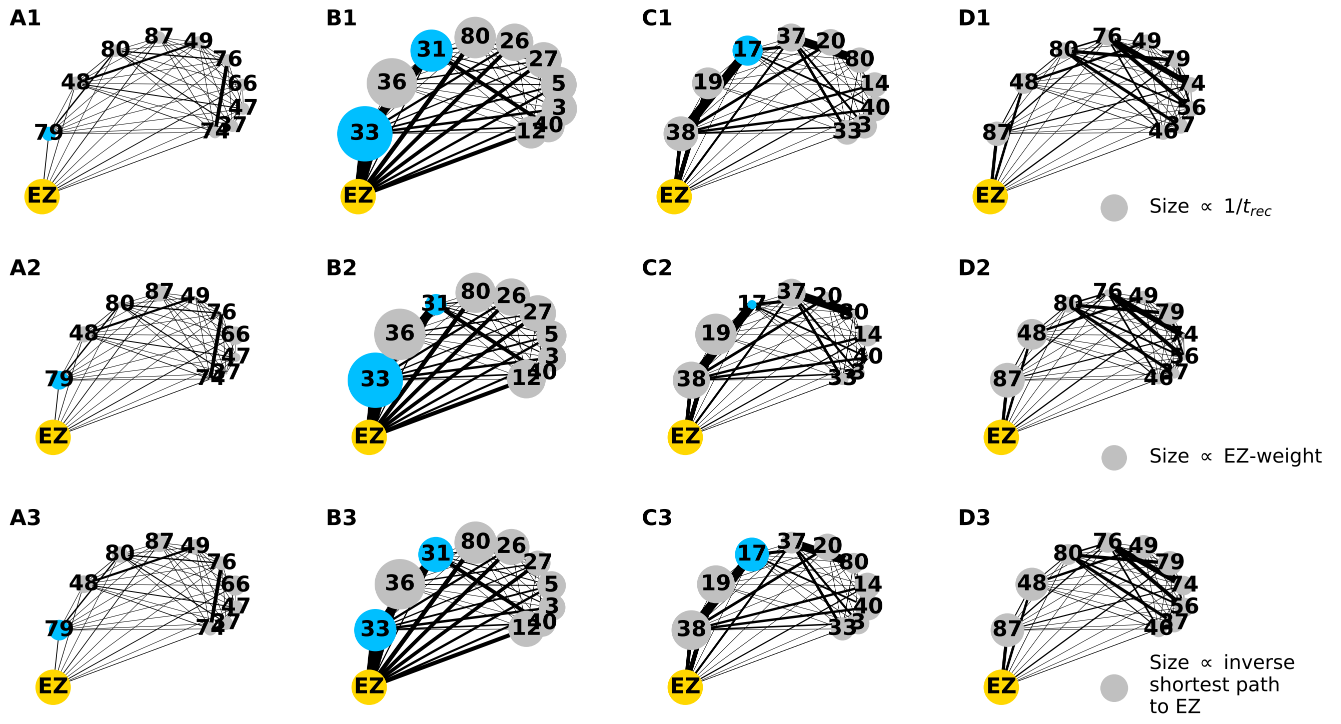

Figure 7. Graph plots for the epileptic patients with more than one area in the EZ. Same as Fig. 12 (Main Text) but for the patients with more than one area in the EZ (yellow node). All areas belonging to the EZ are merged into one for visualization clarity, keeping intact the recruitment order, the recruitment times and the connection weights to the areas in the EZ. Node circle size corresponds to the inverse recruitment time (A1-D1), to the connection strength to the EZ (A2-D2) and to the inverse shortest path length to the EZ (A3-D3). The size of the yellow EZ circle remains fixed. Blue dots distinguish recruited areas to belong to the PZ_{SEEG}, i.e. the PZ identified according to the presurgical invasive evaluation. Results are obtained for patients E1 (panels A1-A3), E4 (panels B1-B3), E5 (panels C1-C3), E7 (panels D1-D3). Note that patient E1 has very weak connections outgoing the EZ which results in very late recruitment times indicated by small circle sizes in A1. For patient E4, the first ten recruited nodes are strongly connected with the EZ. The recruitment of node 31 before node 80 (stronger connected to the EZ with respect to the previous one), is justified by the strong connection to node 36 and the comparable shortest path length. For patient E5 the shortest path length is more determinant than the connection strength to the EZ to determine the recruitment order. The sequentially recruited nodes are particularly strongly connected, thus explaining the recruitment of node 17, weakly connected to the EZ, but strongly connected to the previously recruited node. Patient E4, on the other hand, has very strong connections outgoing the EZ and very short recruitment times indicated by large circle sizes in B1. As shown in Fig. 12, the first recruited node is usually the one with the strongest connection strength to the EZ and with the shortest path to the EZ (apart for the case B3). Parameters as in Fig. 10 (Main Text).

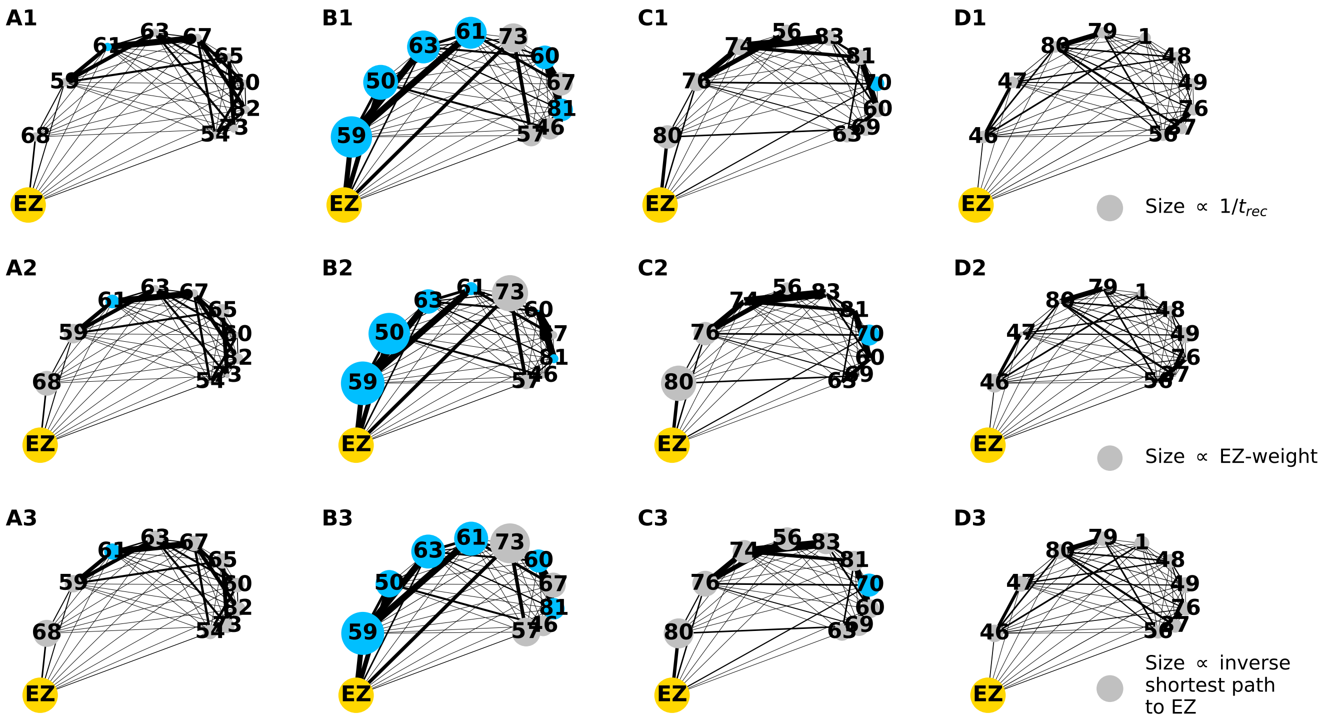

Figure 8. Graph plots for the epileptic patients with more than one area in the EZ. Same as figure 7 but for patients E8 (panels A1-A3), E9 (panels B1-B3), E10 (panels C1-C3), E11 (panels D1-D3). For patient E8, the first two recruited areas show strong connection strengths to the EZ, while the areas recruited later are all characterized by shortest path to the EZ and by strong connections between sequentially recruited nodes. Looking at the graph plots for patient E9, the first four recruited nodes are both strongly connected with the EZ and among them. Node 73, which do not result to belong to the PZ_{SEEG}, is recruited, according to our simulations, due to its topological characteristics: proximity to the EZ, in terms of shortest path length, and high coupling strength. For patient E10, the node 70 (belonging to the PZ_{SEEG}), do not result to be first recruited due to its middle values of connection strength and shortest path, while the nodes that are recruited before are either more strongly connected to the EZ or to the previously recruited node. For patient E11 the recruitment order is mostly determined, as before, by the shortest path length and by the connection strength between sequentially recruited node. As shown in Fig. 12 (Main Text), the first recruited node, for all patients, is the one with the strongest connection strength to the EZ and with the shortest path to the EZ. Parameters as in Fig. 10 (Main Text).

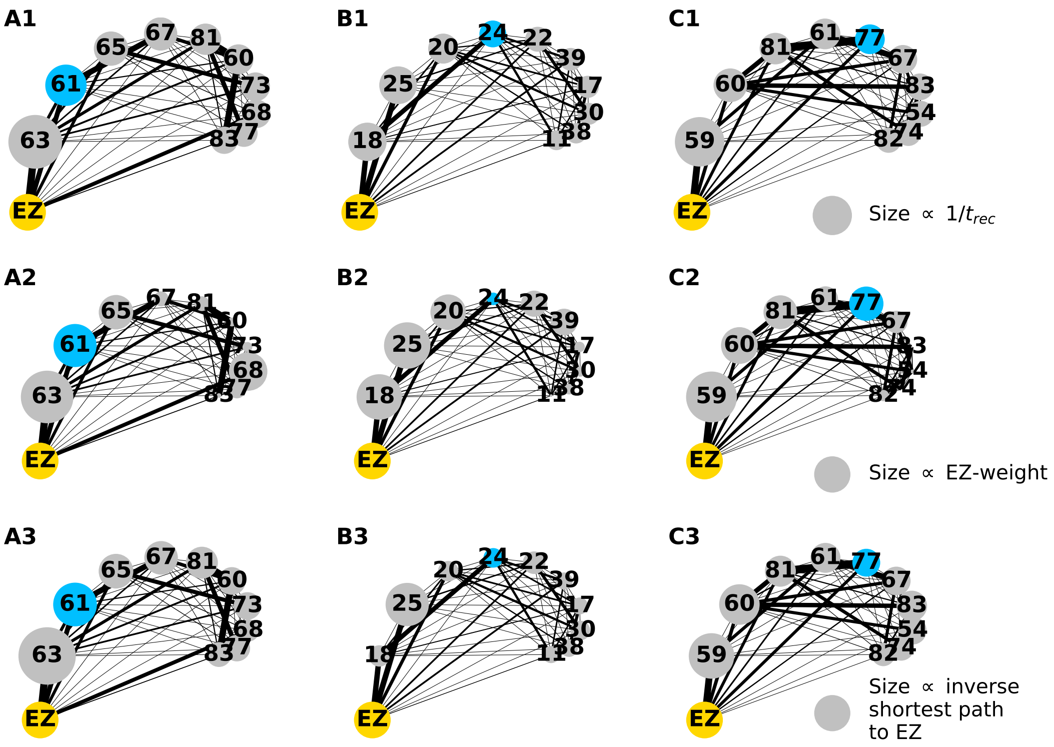

Figure 9. Graph plots for the epileptic patients with more than one area in the EZ. Same as figure 7 but for patients E12 (panels A1-A3), E14 (panels B1-B3), E15 (panels C1-C3). For patients E12 and E15 the recruitment order is mostly determined by the shortest path length to the EZ, supported by the connection strength, which results to be determinant for the first 4-5 recruited nodes. Node 77, belonging to the PZ_{SEEG} for patient E15, do not result to be first recruited due to its middle values of connection strength and shortest path, while the nodes that are recruited before are either more strongly connected to the EZ or to the previously recruited node. On the other hand, for patient E14, the connection strength turns out to be more important than the shortest path to determine the recruitment order. It is worth noticing that node 24, closer to the EZ and strongly connected to node 18, is recruited before nodes 22 and 39 that are more strongly connected to the EZ, but less close in terms of shortest path. As shown in Fig. 12 (Main Text), the first recruited node is usually the one with the strongest connection strength to the EZ and with the shortest path to the EZ (apart for the case B3). Parameters as in Fig. 10 (Main Text).

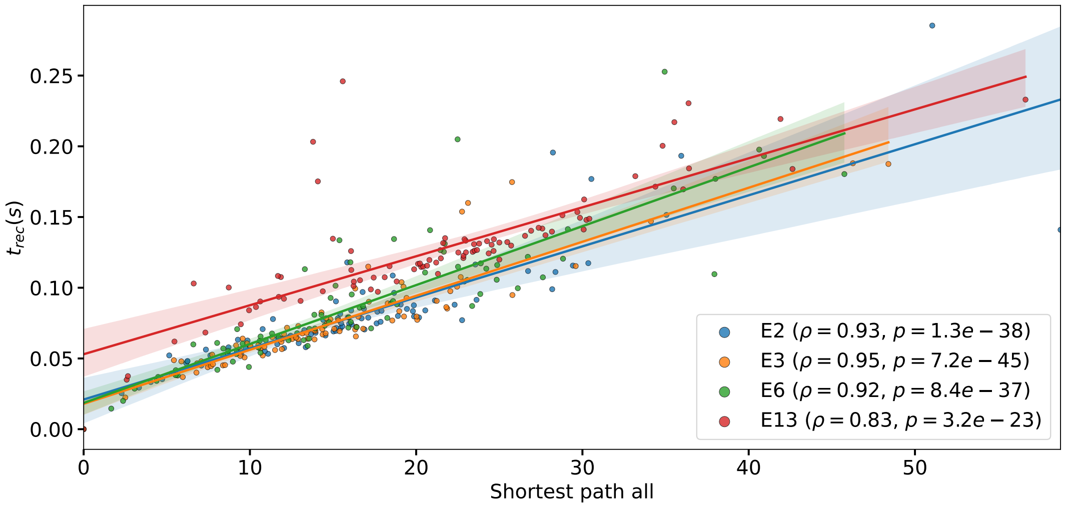

Figure 10. Recruitment time and Shortest Path. The recruitment times t_{rec} as a function of the shortest path to the EZ are shown for four patients and all brain areas. Same as Fig. 13 A (Main Text), with a regression fit that underlines the approximately linear relationship between the shortest path length and the recruitment time. Spearman correlation coefficients ρ and corresponding p-values are significant. Parameters as in Fig. 13 (Main Text).

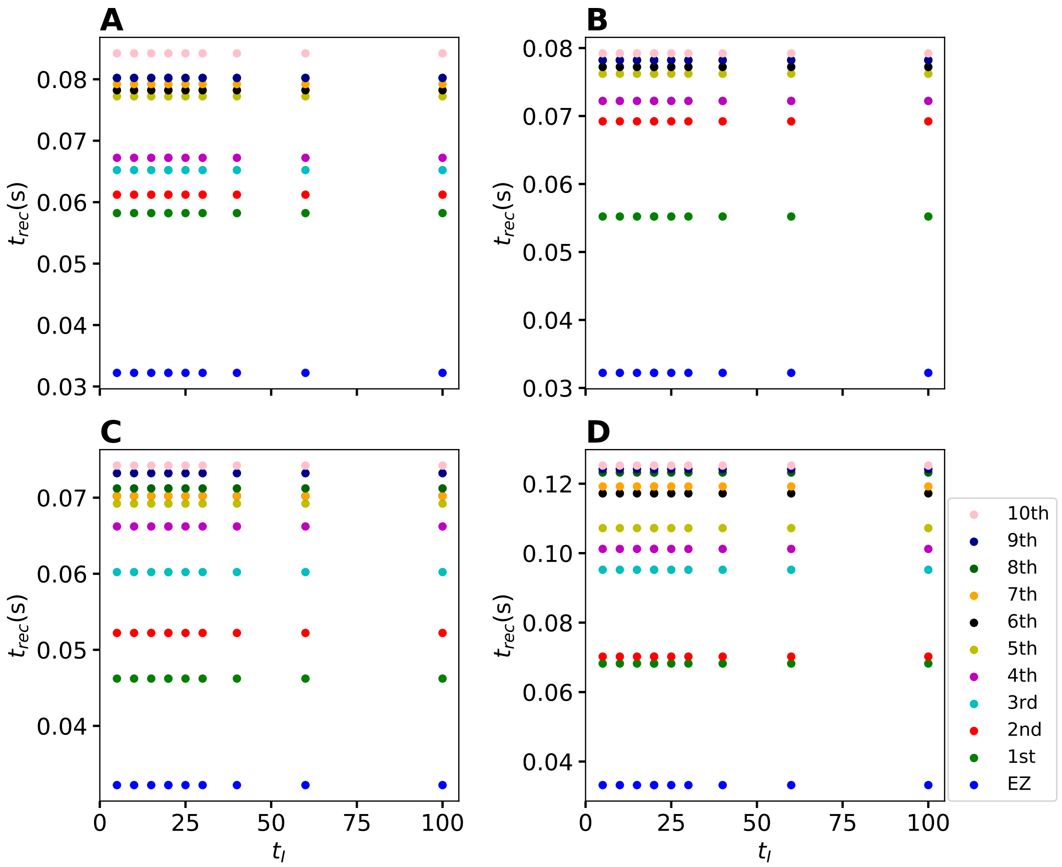

Figure 11. Input Current Duration Variation. Dependence of the recruitment time on the current duration t_I, while the current strength is kept constant at $I_S = 15$, for epileptic patients A) E2; B) E3; C) E6; D) E13. The y-axis shows the recruitment times of the first 10 recruited areas for each current strength. Blue is the EZ, green is the first recruited area, red the second, etc. The recruitment times are independent of the pulse duration. Parameters: $\sigma = 4$, $\Delta = 1$, $\bar{\eta} = -12.5$, $I_S = 15$.
